# ACBM: An Integrated Agent and Constraint Based Modeling Framework for Simulation of Microbial Communities

**DOI:** 10.1038/s41598-020-65659-w

**Published:** 2020-05-26

**Authors:** Emadoddin Karimian, Ehsan Motamedian

**Affiliations:** Department of Biotechnology, Faculty of Chemical Engineering, Tarbiat Modares University, P.O. Box, 14115-143 Tehran, Iran

**Keywords:** Biochemical networks, Multicellular systems

## Abstract

The development of new methods capable of more realistic modeling of microbial communities necessitates that their results be quantitatively comparable with experimental findings. In this research, a new integrated agent and constraint based modeling framework abbreviated ACBM has been proposed that integrates agent-based and constraint-based modeling approaches. ACBM models the cell population in three-dimensional space to predict spatial and temporal dynamics and metabolic interactions. When used to simulate the batch growth of *C. beijerinckii* and two-species communities of *F. prausnitzii* and *B. adolescent*., ACBM improved on predictions made by two previous models. Furthermore, when transcriptomic data were integrated with a metabolic model of *E. coli* to consider intracellular constraints in the metabolism, ACBM accurately predicted growth rate, half-rate constant, and concentration of biomass, glucose, and acidic products over time. The results also show that the framework was able to predict the metabolism changes in the early stationary compared to the log phase. Finally, ACBM was implemented to estimate starved cells under heterogeneous feeding and it was concluded that a percentage of cells are always subject to starvation in a bioreactor with high volume.

## Introduction

A computer model is a simplified representation of a complex system (e.g. bio-populations) that provides a basis for testing and evaluation of hypotheses. Microbial cell models can be either structured or unstructured depending on whether they take into account the details of intracellular reactions and processes or not. The unstructured models such as Monod-type equations consider the cell as a black box and relate input changes to output responses using empirical data. However, they lack any information on the intracellular state and the narrow applicability range of these models makes it difficult to extrapolate the performance of biosystems, especially under perturbed conditions^1^. The structured models consider the microbial cells as multi-component systems and they contain certain details of the intracellular processes. The kinetic models including detailed metabolic reaction kinetics are a well-known category of the structured models. However, kinetic modeling requires a large number of kinetic parameters which are mostly undetermined and they comprise non-linear equations that often require more complicated solution procedures^1^. Metabolic models are widely used structured models that can be constructed using reaction stoichiometry without considering enzyme kinetics. Assuming steady-state condition, the metabolite mass balances are applied to construct metabolic models including linear algebraic equations. Flux balance analysis (FBA) as a constraint-based metabolic modeling approach (CBM)^2^ calculates the fluxes of all reactions in a metabolic model while considering an objective function (e.g. maximization of growth rate). FBA does not generally apply any intracellular constraint and only extracellular constraints including uptake and secretion rates limit the predicted rates. Instead of incorporating the intracellular enzyme kinetics, an upper bound is determined for uptake rates of substrates commonly based on empirical data or if any, the substrate kinetics are used to calculate the extracellular rates using the substrate concentration. The limited understanding of intracellular constraints such as regulatory and signaling events and enzymatic kinetics does not allow for the accurate prediction using these models to expand model reliability over a wide range of environmental conditions. Over the past decade, the integration of omics data with metabolic models has been an important effort to incorporate the intracellular constraints within the metabolic models. In particular, several FBA-driven algorithms have been developed to integrate transcriptomics data into metabolic models^3^.

The cell models can also be categorized to segregated or unsegregated, referring to whether they consider heterogeneity in the cell population or not. The metabolic models have mostly been applied within the unsegregated modeling framework, however, some approaches have been developed to model metabolic interactions within microbial communities. The first approach constructed a generalized metabolic model by combining the reconstructions of single microbes and considered community growth as the objective function of FBA, e.g.^4^. Subsequent approaches included temporal dynamics using dynamic FBA (dFBA) to simulate microbial growth^5^. Then, COMETS was introduced^6^ that incorporates spatial dynamics by integrating dFBA with diffusion on a lattice. Physical space in 2D was discretized into boxes that contained different microbial species and extracellular metabolites. The biomass and extracellular metabolite levels were updated in each time step using dFBA for each box based on the nutrients available in the environment and on the capacity of the metabolism. COMETS estimated the upper bounds of substrate uptake rates using a kinetic equation. This method is an equation-based model that describes biological processes by formulating interactions of individual biological components using a system of equations for variables in time and space. Indeed, by simulating colony growth as a two-dimensional diffusion, COMETS represents the space of the boxes continuously by assigning each box for a population of multiple cells. So it can not model each individual cell to investigate metabolic heterogeneity within a population of cells.

In recent years, individual-based models have been applied to consider heterogeneity in the cell population. They simulate populations and communities by following individuals and their properties, and agent-based models (ABMs) are a class of computational models for individual-based modeling. ABM models space as a heterogeneous environment in which individuals are represented as agents and move. Agents obey simple rules and ABMs consisting of dynamically interacting rule-based agents can result in different sorts of complex behavior. By incorporating ABM and CBM, Biggs and Papin^7^ proposed a tool named MatNet for multiscale modeling of *P. aeruginosa* biofilm formation. The hybrid model was capable of modeling biofilm formation of a single species and qualitatively predicted the effect of oxygen limitation, nitrate addition, and gene knockout. Shashkova *et al*.^8^ used ABM and kinetic equations to model interactions between two bacterial species and between species of the gut. Their model was able to observe the emergent spatial structure and its alteration, depending on various feedback mechanisms. BacArena^9^ applied ABM and FBA to simulate multispecies communities and considered a two-dimensional grid to model a spatial environment. Temporal dynamics were modeled by including time steps, and the substrate uptake rate was constrained using the Michaelis-Menten kinetic equation. In comparison with COMETS as an equation-based and continuous method, BacArena models additional heterogeneity of cells by focusing on individuals using ABM, and this rule-based method improved the predicted doubling time for *Clostridium beijerinckii*. The predicted metabolite concentration ratio for acetate was comparable to experimental values for co-cultures of *in vitro* human intestinal microbiota while higher and lower ratios were calculated for butyrate and propionate, respectively.

**Figure 1:**
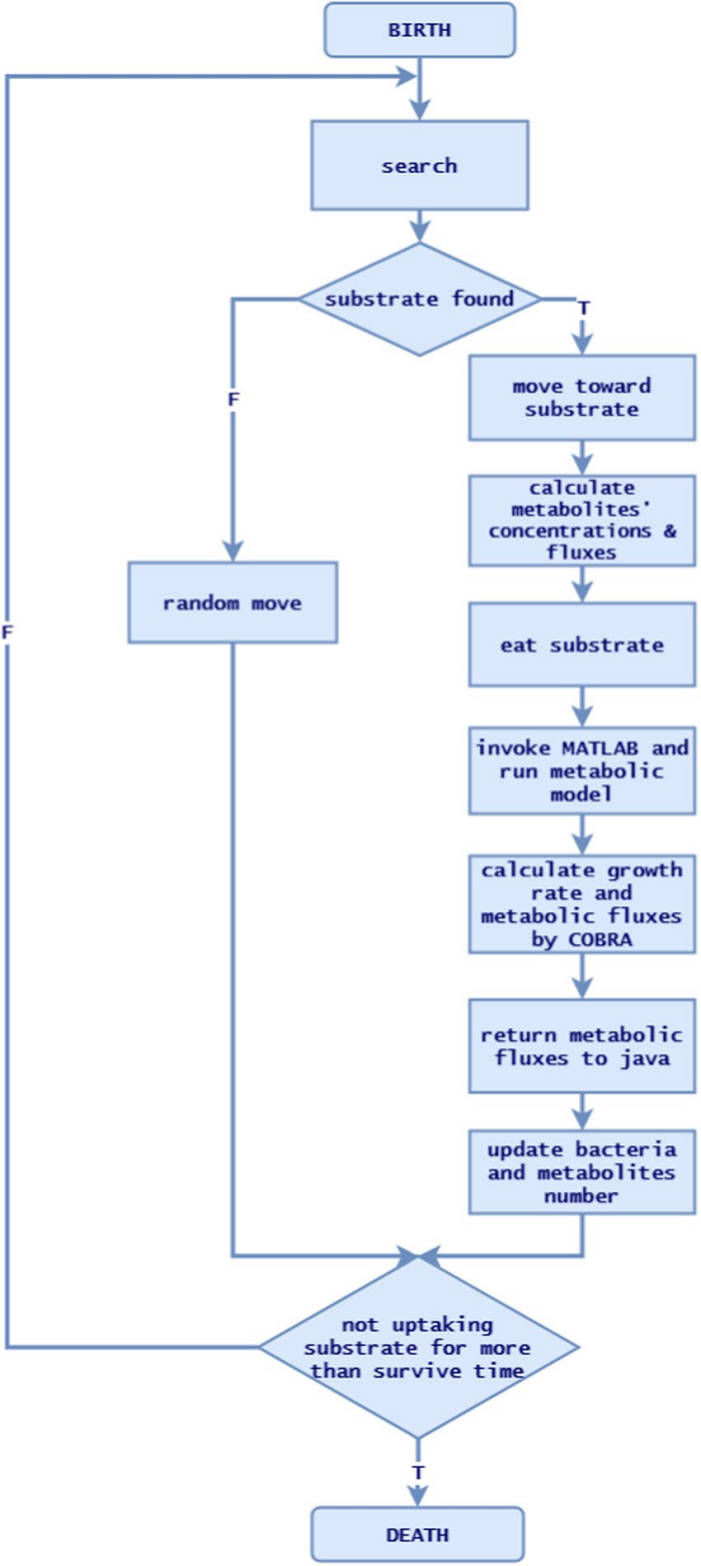
Flowchart of the cell process developed for ACBM.

The proposed methods were capable of identifying the microbial community structure by considering spatial and temporal multi-scale modeling approaches. Furthermore, BacArena and MatNet combined individual based modeling with FBA to consider the metabolic heterogeneity within a population of cells. Except for MatNet, the methods can model multi-species communities. However, it is still necessary to develop new methods that present results quantitatively comparable with experimental data of a bioprocess such as batch and fed-batch growth. In this research, a new integrated agent and constraint based modeling framework abbreviated ACBM (Fig. 1) has been proposed that integrates ABM and CBM similar to BacArena and MatNet but with a different formulation. Indeed, ACBM is a structured and segregated model that uses ABM and CBM to apply intracellular (e.g., the capacity of the metabolism) and extracellular (e.g., the nutrients available in the environment) constraints^10^ of a cell, respectively. Thus, it can properly simulate the temporal and spatial dynamics of a cell population in different processes, such as batch and fed-batch growth. Compared to its predecessors, ACBM models microbial populations in three-dimensional space and makes predictions using mechanistic processes that more closely mimic the intra- and extracellular behaviors present in living microbes. Using substrate kinetics, ACBM was applied to simulate batch growth of *C. beijerinckii* and two-species communities of *F. prausnitzii* and *B. adolescenti*. Furthermore, transcriptomic data were integrated with a metabolic model of *E. coli* to consider intracellular constraints in the metabolism. Glucose concentration is a critical parameter for both productivity and quality in a fed-batch process of recombinant protein production. So, ACBM was used to estimate starved cells in a bioreactor with high cell density.

## Results and Discussion

### Simulation of batch growth

**Figure 2:**
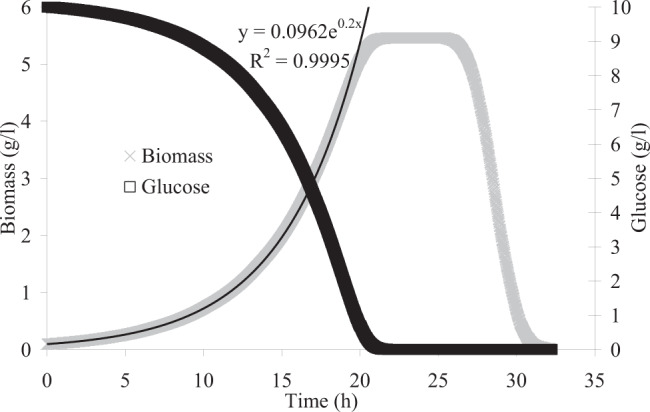
The predicted biomass and glucose concentrations by ACBM in a batch culture of *C. beijerinckii* including 10 g/l glucose.

ACBM was used to predict the growth of *C. beijerinckii* in a batch culture including 10 g/l glucose and kinetic equation proposed by Bauer *et al*.^9^ was applied to calculate glucose uptake rate using glucose concentration. Figure 2 shows that ACBM properly predicts the life cycle of cells and the phases of batch growth including accelerating, logarithmic, decelerating, stationary, and death. Given that ACBM is stochastic because of the random movement of cells and metabolites, ACBM was implemented three times to evaluate the variation of the predicted concentrations for biomass and glucose in Fig. 2. Variations in the predicted values were negligible and a maximum standard deviation of 0.031 g/l was calculated. The high concentration of either substrate or biomass is the reason for the low error. Indeed, the probability of finding substrates by cells at each time step is always constant since the concentration of either glucose or biomass or both is high.

The growth rate at the exponential phase equals 0.2 1/h, hence, ACBM predicts a doubling time of 3.47 h for *C. beijerinckii*. Two previous approaches BacArena and COMETS predicted a doubling time of 1.1 and 0.5 h, respectively, for *C. beijerinckii* that is much smaller than the experimental value of 4.3 h^11^. So, ACBM improved the predicted growth rate while it used a metabolic model and kinetic equation for substrate uptake the same as BacArena and COMETS.

However, it overpredicted the growth rate that can be because of the lack of intracellular constraints. Cells are always faced with two intracellular and extracellular constraints for growth^10^. In the stationary phase, when a cell is under starvation and could not find any substrate, the extracellular constraint of lack of substrate controls the growth. So, ACBM does not apply the metabolic model and the cell moves randomly. When the cell finds substrate, it eats metabolites and ACBM applies the metabolic model to predict the growth and secretion rates. When the substrate concentration around the cell is high, overflow can occur and by-products can be produced. However, FBA does not generally apply any intracellular constraint and only extracellular constraints including uptake and secretion rates limit the predicted growth rate^10^. Hence, when ACBM applies FBA, the glucose uptake rate is determined using the Michaelis-Menten kinetic equation. This equation predicts the substrate uptake rate by using glucose concentration and considers a maximum glucose uptake, but FBA can not predict the overflow metabolism and the condition that glucose is abundant. So, it linearly increases the growth rate with an increase in glucose uptake rate and it can be the main reason for the overprediction of growth by ACBM when FBA is used.

### Simulation of cross-feeding

**Figure 3:**
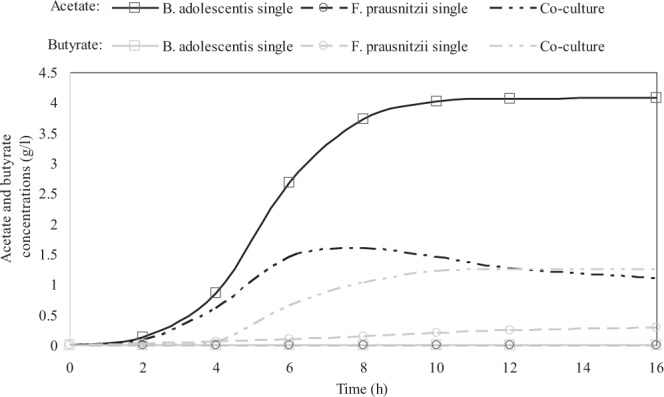
Comparison of the predicted acetate and butyrate concentrations in single and co-cultures of *B. adolescentis* and *F. prausnitzii*. Black and grey colors are for acetate and butyrate, respectively.

Cross-feeding is an important metabolic interaction mechanism especially between bacteria inhabiting the human intestine such as Bifidobacterium and Faecalibacterium genera^8^. *B. adolescentis* produces acetate and *F. prausnitzii* metabolizes this acetate to butyrate. ACBM was implemented to simulate single- and two-species communities of *F. prausnitzii* and *B. adolescentis* (Fig. 3). *F. prausnitzii* produced a little amount of butyrate (0.3 g/l) while the produced butyrate in the co-culture increased more than four times. It shows that in the co-culture, *F. prausnitzi*i has been able to synthesize butyrate by consuming acetate. The experimental results of Rios-Covian *et al*.^12^ also show the enhanced formation of butyrate by *F. prausnitzii* (two to four times) in the presence of the bifidobacteria compared to the *F. prausnitzii* monocultures. Simulation results reveal that *B. adolescentis* produces about 4 g/l acetate in the monocultures, which it reduces to 1.1 g/l in the co-culture.

### Integration of transcriptomics data

**Figure 4:**
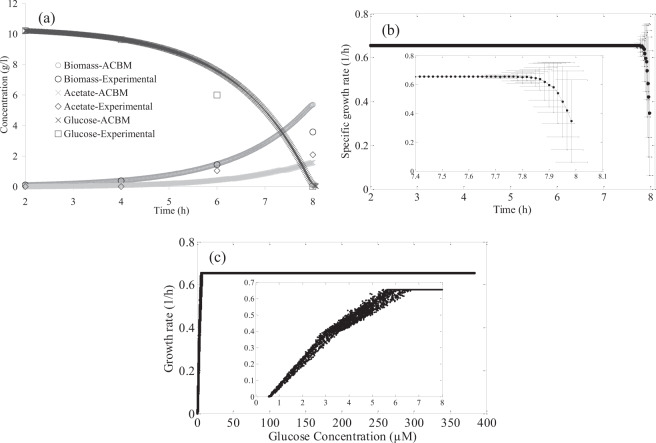
(**a**) concentrations of biomass, glucose, and acetate predicted by ACBM and measured by Rahman *et al*.^13^, (**b**) change of average specific growth rate versus time (the bars represent the distribution of specific growth rate in the community), and (**c**) growth rate versus glucose concentration predicted by ACBM.

Using the substrate kinetics or determining an upper bound for uptake rates of substrates does not implement the intracellular constraints and kinetic data of intracellular reactions are limited. So, transcriptomics data of aerobic growth of *E. coli* MG1655 were integrated with the metabolic model iJO1366 reconstructed for *E. coli* MG1655 using TRFBA to incorporate the intracellular constraints. Figure 4(a) indicates the predicted concentrations for biomass, glucose, and acetate in comparison with experimental data^13^. Glucose is depleted at about 8 h that is experimentally confirmed. The predicted biomass is fully consistent with the experimental data for 6 h, but the higher concentration of biomass is observed at 8 h. The discrepancy at the end of growth can be attributed to a variety of issues such as ignorance of the toxic effect of organic acid accumulation and pH change by ACBM. The acetate concentration is negligible during 4 h of growth and then, it increases to 1.68 and 2 g/l at the end of the exponential phase based on the predicted and measured values, respectively. A 2D view of the environment during the batch cultivation is presented in supplementary file 4 as an MP4 format video file. ACBM truly predicts that a small amount of lactate is produced and succinate is not secreted during batch growth. Production of formate, a major fermentation product of *E. coli*^14^, is also predicted.

Figure 4(b) demonstrates that the exponential phase continues up to 7.6 h at the constant growth rate of 0.65 1/h and so, the predicted doubling time (64 min) is comparable to the experimental value of 70 min^13^. Charbon *et al*.^15^ also reported a doubling time of 60 min for *E. coli* MG1655 when cells were grown in a minimal medium supplemented with glucose. At the late log phase, glucose is insufficient for all cells and exponential growth can not be sustained. Then, cells are subject to starvation, the average growth rate of the population decreases, and the variation of growth rate increases. Rahman *et al*.^13^ also mentioned that the end of the log phase is correlated with glucose depletion.

The proper prediction of fermentation products is because of TRFBA, which applies the intracellular constraints on the metabolism of the cell. When ACBM applies TRFBA and the transcriptomics data, the kinetic equation is not required and the extracellular constraint of the substrate concentration and the intracellular constraints applied by TRFBA predict glucose uptake rate and growth and product secretion rates. TRFBA limits the maximum rate of metabolic reactions based on the expression level of their corresponding genes so that an increase in substrate uptake rate can cause some reactions to reach their maximum rate and result in a compulsory change in metabolism^10^. Thus, using pathways with lower growth yield wasted glucose to the fermentation products.

Figure 4(c) shows that the growth rate versus glucose concentration takes the form of the Monod equation with half-rate constant (K_s_) of 2.72 µM. It demonstrates that glucose is the limiting substrate for growth. The K_s_ value of 3 and 6 µM were reported by Bavoil *et al*.^16^ and Nikaido and Rosenberg^17^, respectively, for wild-type *E. coli* growing on a glucose-minimal medium that is comparable with the predicted K_s_.

Average reaction fluxes predicted by ACBM for 22 enzymes of *E. coli* central metabolic pathways at exponential (6 h) and early stationary (8 h) phases of growth were compared to specific activities of these enzymes measured by Rahman *et al*.^13^. The results show that the framework was able to predict the metabolism changes in the early stationary phase compared to the log phase. Especially, ACBM properly predicted flux reduction of glycolytic pathway and activation of the glyoxylate pathway enzymes Icl and MS at the early stationary phase. Both rates and activities of reactions catalyzed by acetate kinase and lactate dehydrogenase significantly reduced and cells declined excretion of organic acids lactate and acetate at the late log phase.

Ratios of fluxes at the log and the early stationary were calculated and compared to the ratios for enzyme activities (supplementary file 3, Table S2). The Spearman correlation coefficient of 0.47 demonstrates that the relationship between flux and activity ratios is monotonic. The small P-value of 0.027 indicates that the coefficient is significantly different from zero.

### Effect of substrate starvation

**Figure 5:**
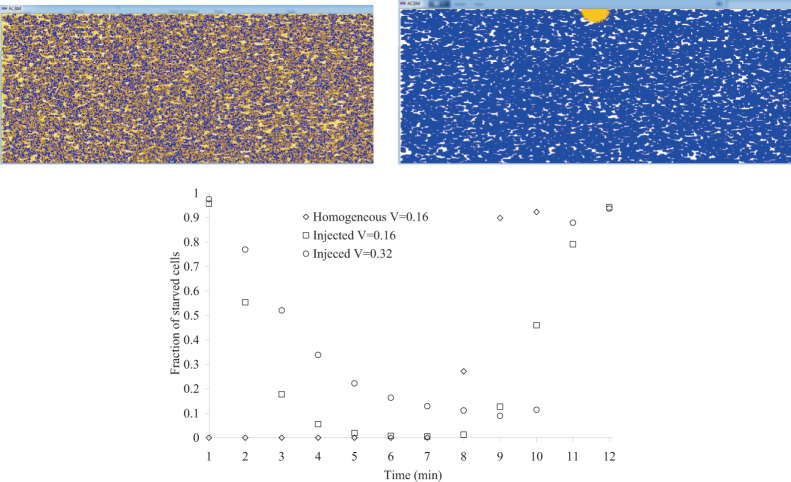
(**a**) homogeneous and (**b**) heterogeneous (from coordinate x = 500 µm, y = 0, and z = 0) feeding of 10 g/l glucose in a microbioreactor including 60 g/l biomass of *E. coli*. (**c**) percentage of starved cells over time (V: microbioreactor volume in µl).

Lack of control in substrate starvation is always risky for the production process and so ACBM was applied to estimate starved cells in a bioreactor. The fraction of starved cells overtime was determined under two homogeneous and heterogeneous feeding of 10 g/l glucose according to Fig. 5(a-b). For the simulation of fed-batch culture, heterogeneous feeding was modeled and glucose was injected from the middle point of microbioreactor including high cell density of *E. coli* (60 g/l). As observed in Fig. 5(c), for homogeneous distribution, all of the cells find glucose until depletion. However, more than 95% of cells are subject to starvation at the first minute of glucose injection and some cells are starved for up to 7 min. The twofold increase in the volume of the simulated cubic microbioreactor from 0.16 to 0.32 µl enhances not only the number but also the fraction of starved cells. Furthermore, a percentage of the cells can not find the nutrient in all of the time steps. So the injection of glucose with more concentration or more stirring speed can be effective.

Figure S1 indicates the effect of stirring speed under heterogeneous feeding on the percentage of starved cells over time. Increasing the speed from 8000 to 12000 µm/h significantly reduces the fraction of starved cells. However, Figure S1 demonstrates that the further increase in stirring speed does not result in the reduction of starved cells. Figure S2 demonstrates the effect of an increase in glucose concentration from 10 to 20 g/l. ACBM predicts that a two-fold increase in glucose concentration resulted in a slight reduction of the starved cells.

### Discussion

In this research, a segregated and structured model was introduced to simulate microbial growth. The agent-based modeling approach was used to provide a segregated model at the multicellular level and the life cycle of cells was simulated. Glucose concentration spatially and temporally differentiates the population into separate phenotypes. In the log phase, glucose is sufficient for all cells and a homogeneous growth is observed. But at the end of this phase, the spatial concentration gradient and differential substrate availability lead to the distribution of phenotypes. Some of the microbial cells are metabolically active with different glucose uptake rates and some of them are metabolically inactive and under starvation. ACBM is also a structured model with incorporating the constraint-based modeling approach and the genome-scale metabolic models. Furthermore, transcriptomic data were integrated with a metabolic model to consider the intracellular metabolic constraints in addition to the extracellular glucose availability constraint.

The improvement of predictions by the ACBM framework compared to the previous approaches can be attributed to the different formulation of ACBM. For example, the process of searching nutrients is analogous to a real biological system and using physical and morphological properties of cells for simulation improves the predictions. BacArena and COMETS model growth as a 2D diffusion while using ACBM, cells search for nutrients in a three-dimensional x, y, z environment. The appropriate prediction of concentrations for biomass, glucose and acidic products in batch culture of *E. coli* demonstrates that by combining an individual-based model with a metabolic model integrated with transcriptomic data, ACBM is capable of correlating the predicted fluxes with concentrations within a cell population.

Starvation is one of the main sources of stress that induces cell death via apoptosis and necrosis in the cell^18^. The level of substrate limitation is crucial, especially for the fed-batch process of recombinant protein production, since it may cause undesired glucose starvation and leads to reduced cell growth and productivity and altered N-glycosylation quality^19^. Lack of control in substrate starvation is always risky for the production process and so ACBM was applied to estimate starved cells in a bioreactor. The results demonstrate that ACBM is a reliable tool for studying the starvation periods in sequential substrate injections of a fed-batch culture. Considering that a proper feeding strategy prevents glucose starvation, ACBM can be used to evaluate glucose concentration, periods of substrate injection, and stirring speed for different strategies. Starvation rates of high-volume bioreactor batch cultures have been neither computationally nor experimentally observed, so our predictions present an opportunity for future experimental validation.

## Materials and methods

### Materials

The basis of framework, which was designed with the agent-based method, was constructed by object-oriented programming in Java 1.8. MATLAB 2015b and COBRA 2.05 Toolbox^20^ was used for constraint-based modeling using GLPK as a solver for linear programming (LP) problems. MATLAB Control Java library 4.1.0 connects the two programming environments. To run the framework for each species, its genome-scale metabolic model was used and biomass formation was used as the objective function to be maximized by solving the LP problem using FBA or TRFBA^2^. A list of the used metabolic models is presented in Table 1.

**Table 1:** Genome-scale metabolic models used in this research.

Species	Genome-scale metabolic model	Reference
*Clostridium beijerinckii* NCIMB 8052	*i*CM925	^11^
Bifidobacterium adolescentis L2-32	*i*Bif452	^22^
Faecalibacterium prausnitzii A2-165	*i*Fap484	^22^
*Escherichia coli* MG1655	*i*JO1366	^2^

### ACBM representation

#### Framework structure

**Figure 6:**
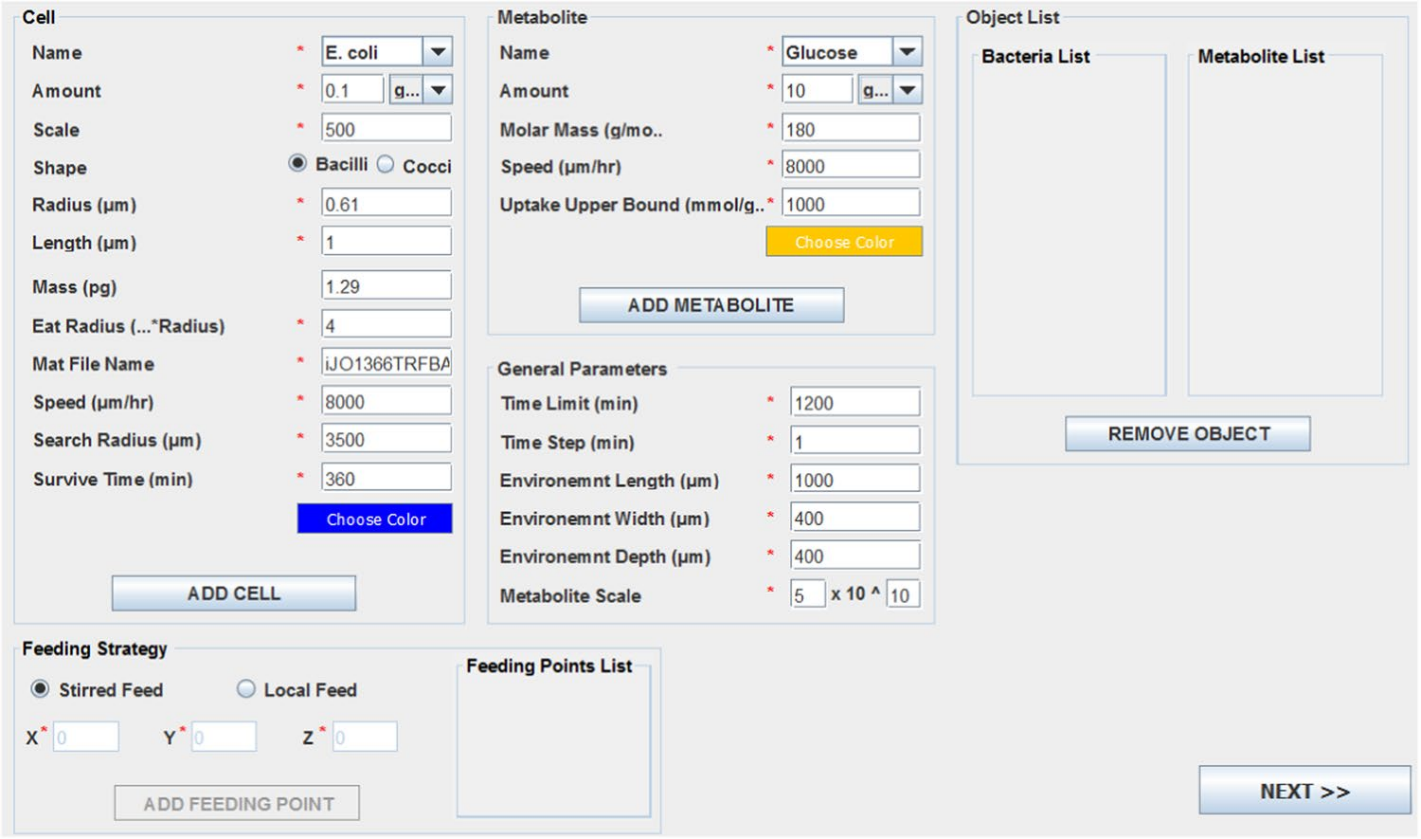
Designed GUI panel to perform ACBM.

The represented multi-scale model is agent-based in Java as the main platform for the implementation of ACBM, which calls MATLAB to apply a constraint-based metabolic model in each time step for predicting growth rate and uptake and secretion rates by each cell individual. Model agents are cells, metabolites and environment and each agent of the model is created as an object in Java. The framework can be applied for any environment and any kind and number of microorganisms and metabolites, and simulation of up to hundreds of species is possible by ACBM. Each agent in the model is equivalent to an object in Java and so, there is an object for each cell, metabolite, and environment. A graphical user interface (GUI) panel according to Fig. 6 was designed to perform ACBM in a user-friendly way. The user manual and the codes of ACBM are presented in supplementary files 1 and 2, respectively.

At start time (t = 0), one environment and a specific number of cells and metabolites according to their concentration in the environment are considered and then, in each time step, the number of each type of cells and metabolites are updated based on the rates predicted by the metabolic model.

#### Environment object

An environment object contains other objects (cells and metabolites) and is considered as a cube with basic properties length (x), width (y) and depth (z). The simulated environment can be part or all of an original environment. For example, it is not necessary to simulate the entire environment of a fully mixed bioreactor and simulation of a small cube indicates not only the changes inside the bioreactor but also reduces the CPU time. In this research, a small cube with a size of 1 × 0.4 × 0.4 mm was considered for simulation of batch growth in a stirred bioreactor that was completely mixed in the macro-scale. It should be mentioned that the system can be heterogeneous in millimeter-scale because of the stochastic movement of cells and metabolites.

#### Cell and metabolite object

Cells and metabolites move randomly in the environment. For implementing a random movement function, a random unit vector is first formed by generating two random angles (φ, θ) between 0 and 2π. Thus, the unit vector is d = (cos φ cos θ, cos φ sin θ, sin φ). Then, this vector is multiplied by speed entered in the panel to provide velocity vector (v cos φ cos θ, v cos φ sin θ, v sin φ). When agitating is not used and water is stagnant, diffusion is important. But a stirred bioreactor was simulated in this research and mass transfer by convection was dominant. So, the same speed of 8000 µm/h was considered for both objects (cell and metabolites) and this speed was sufficiently enough for the objects to move quickly while experimental values of speed are much higher. Metabolites are produced or consumed by cells and their initial number is calculated based on their initial concentration. To reduce the number of metabolite objects as well as CPU time, a certain number of each metabolite (5 × 10^5^) was considered as an object including a package of metabolites.

The cell is the main object of this modeling and all cells spend a specified time process that is presented in the next section. The specific properties of each cell type were used in the process. (Table 2). The information for some microorganisms is presented in Table S1 (supplementary file 3). The initial number of each cell type was calculated by considering its initial concentration and volume of the environment. To reduce CPU time, a specific number of cells was considered as a colony of cells that spend the cell process together. Two shapes for each cell type including bacilli or cocci can be selected in the panel. To consider space occupation by cells, the environment is divided into cube elements with an edge of 1 µm to generate meshing. Each cell occupies a number of the elements according to its size. According to the randomly generated velocity vector, a cell selects the destination elements for movement and checks whether the selected elements are filled and if not, it does a movement. In the case of filled elements, other velocity vectors are generated and applied to find an empty space.

**Table 2:** The properties used to implement the time process for each cell.

Property	Description	Unit
Radius	Average radius of one cell	µm
Length	Average length of one bacilli cell	µm
Volume	Average volume of one cell	µm^3^
Mass	Average mass of one cell	pg
Search radius	Searching radius	µm
Eat radius	Eating radius	µm
Mat file name	Name of metabolic model	—
speed	Movement speed	µm/s
Search radius	Searching radius	µm
Survive time	Time that a cell can survive without eating	min

#### Cell process

Cell actions include searching metabolite, eating metabolite, biomass and metabolite production, division, death, and movement. In each time step, cell searches for metabolites and finds the nearest one. If it could not find any substrate in the eat radius, moves randomly. Otherwise, it moves toward the metabolite package. Then, the cell eats metabolites and moves randomly and the consumed metabolites are eliminated.

At the end of each time step, the number of produced cells and metabolites and consumed substrates is calculated by calling MATLAB and applying the metabolic model. To determine the number of produced cells and metabolites, the upper bound of the substrate uptake rate should first be calculated from the number of consumed metabolites as substrate. For this purpose, it is assumed that all found substrates can be consumed and the change in substrate concentration (ΔC) is specified from the number of found substrates (n) using Eq. 1.

1 $$\Delta {\rm{C}}=\frac{{\rm{n}}}{{{\rm{n}}}_{{\rm{a}}}{\rm{V}}}$$

where n_a_ = 6.022 × 10^23^ 1/mol is the Avogadro constant that indicates the number of molecules contained in one mole of substrate and V is the eating volume (a sphere with the eating radius determined in the panel). Substrate uptake rate (*v*) is calculated using Eq. 2.

2 $${\rm{v}}=\frac{\Delta {\rm{C}}}{{\rm{x}}\Delta {\rm{t}}}$$

where x and Δt denote biomass concentration and time step, respectively. Considering a short time step, it can be assumed that the change in biomass concentration is negligible and hence, x equals the biomass concentration at the beginning of the time step (x_i_). In this research, a short time step (Δt = 1 min) was considered. To determine the biomass concentration, the average mass of one cell is multiplied to the number of cells and is divided by the eating volume. If the imported upper bound of uptake for each substrate in the GUI panel is less than the calculated rate by Eq. 2, the imported value is selected as the uptake rate. In this research, the maximum uptake rate of 1000 was written in the panel for glucose for all of the simulations. Except for *E. coli*, the maximum uptake rate was determined using the glucose concentration (ΔC) and the Michaelis–Menten kinetic equation proposed by Bauer *et al*.^9^ and then, FBA was applied. For *E. coli*, transcriptomics data of Covert *et al*.^21^ were integrated with the metabolic model using TRFBA^3^ and hence, the glucose uptake rate was automatically limited by intracellular constraints. The transcriptomics data were obtained from the exponential phase of batch growth in a bioreactor and total RNA was isolated from exponentially growing cells.

The calculated fluxes are sent from Java to MATLAB and the substrate uptake rate is bound in the metabolic model. Considering the maximization of growth rate as the objective function, the production rates of biomass and metabolites and uptake rates of substrates are calculated which are returned to Java. Then, the concentration of produced biomass and metabolites and consumed substrates is calculated in Java using Eqs. 3–5.

3 $${{\rm{x}}}_{{\rm{i}}+1}{={\rm{x}}}_{{\rm{i}}}{{\rm{e}}}^{{\rm{\mu }}\Delta {\rm{t}}}$$

4 $${C}_{i+1,p}={C}_{i,p}+{v}_{p}\times {x}_{i}\times \Delta t$$

5 $${C}_{i+1,s}={C}_{i,s}-{v}_{s}\times {x}_{i}\times \Delta t$$

where indices i and i + 1 demonstrate values at the beginning and end of a time step, respectively, and indices p and s are abbreviations of product and substrate, respectively. µ, *v*_p_, *v*_s_ are specific growth, product formation, and substrate uptake rates, respectively, predicted by FBA or TRFBA. Mass of produced biomass and products and consumed substrates by each cell is calculated and then, number of produced cells and metabolites and consumed substrates is determined and new objects are added to the environment and consumed objects are removed. When the total biomass of an individual reaches twice the average mass of a cell, a new cell object is born and placed at an empty space. The time steps continue until all cells die. If a cell can not find the substrate within the survive time, it dies. The flowchart of the cell process is presented in Fig. 1.

## Supplementary information

Supplementary file 1.

Supplementary file 2.

Supplementary file 3.

Supplementary file 4.

## Data Availability

All relevant data are within the paper.
