# Supplementary material for: ACBM: An Integrated Agent and Constraint Based Modeling Framework for Simulation of Microbial Communities
Source: Sci Rep. 2020 May 26;10:8695. doi: 10.1038/s41598-020-65659-w (PMC7250870; doi:10.1038/s41598-020-65659-w)
Supplement: Supplementary file 1 [file 41598_2020_65659_MOESM1_ESM.pdf]

ACBM: An Integrated Agent and Constraint Based Modeling Framework for Simulation of Microbial Communities

Emadoddin Karimian and Ehsan Motamedian

Supplementary file 1

ACBM: AN INTEGRATED AGENT AND CONSTRAINT BASED MODELING FRAMEWORK FOR SIMULATION OF MICROBIAL COMMUNITIES

User Manual

September 2019

1. ABOUT

ACBM is a multi-scale modeling framework for simulation of microbial growth in communities. It integrates agent-based and constraint-based modeling approaches. ACBM can be used for all microbial communities containing any type and any number of cells and metabolites.

The basis of the framework is designed using the agent-based method and is implemented by object-oriented programming in Java 1.8. MATLAB 2015b and COBRA 2.05 Toolbox is used for constraint-based modeling and the solver is GLPK. Matlab Control Java library connects the two programming environments (the library is included in the supplementary file 2).

2. REQUIREMENTS

To run the model you need to have these applications installed:

1. Java 1.8 or upper for installation go to https://www.oracle.com/technetwork/java/javase/downloads/index.html
2. Matlab
3. COBRA Toolbox for Matlab for installation go to https://opencobra.github.io/cobratoolbox/stable/installation.html
4. GLPK solver

3. RUNNING SIMULATION

To run ACBM for your desired system follow these steps.

1. Download supplementary file 2 (supplementary file 2-ACBM1.4.rar) and extract it
2. Download metabolic models of each type of bacteria existing in your microbial system
3. If metabolic model files are in XML or excel format convert them to MAT files (more explanations in section 3.1)
4. Place MAT files in ACBM folder
5. Execute ACBM simply with opening "ACBM.jar" existing in ACBM folder
6. Enter each cell properties and then click "ADD" to add them to your microbial system (more explanations in section 3.2)
7. Enter each metabolite properties and then click "ADD" to add them to your microbial system (more explanations in section 3.3)
8. Enter general parameters of simulation (more explanations in section 3.4)

9. Determine feeding points (more explanations in section 3.5).
10. After adding all objects and entering general parameters, click “NEXT”
11. Enter information of exchange reactions for each metabolite in each bacteria metabolic model (more explanations in section 3.6)
12. Click “RUN”

3.1. How to convert xml and excel metabolic model files to mat file

1. Read xml or excel file with Matlab using this command: readCbModel('xml_file_name', 'model')
2. Save it as mat file using this command:
   save(‘mat_file_name’, ‘model’)

3.2. Adding Cells

Adding cells in ACBM is quite simple as mentioned in step 6. You have to enter specific properties of that cell in the first window of the designed GUI panel (Figure S1) after opening ACBM and then click “ADD”. These properties include the following items:

1. Name
2. Amount: initial amount (you can choose its unit to be g/l or count)
3. Scale: a specific number of cells considered as a colony of cells that spend the cell process together
4. Shape: the shape of the cell (Bacilli or Cocci)
5. Radius (µm): radius of one cell
6. Length (µm): length of one cell (not needed if the cell is a coccus)
7. Mass (pg): volume of one cell (It is for more precise calculations and you can leave it blank to be calculated automatically using volume estimated by radius and length, considering density number of 1.1 g/l (Martínez-Salas et al, 1981))
8. Eat Radius: cell eats nutrients which is available within this radius. The number you enter will be multiplied to the radius of the cell (length in case of bacilli). For example, if you enter 2.5 and radius of bacteria is 0.6, eat radius will be 2.5×0.6 = 1.5 µm)
9. MAT File Name: metabolic model file name you placed in ACBM folder
10. Speed (µm/hours): cell moves at this speed

11. Search Radius (µm): cell can sense and find nutrients within this radius
12. Survive Time (min): average lifetime of a cell that survives without nutrients. The cell will die if this time pass and could not find nutrient to eat.
13. Color: color to show this type of cells in simulation.

For example, the information for *E. coli* is presented in Figure S1.

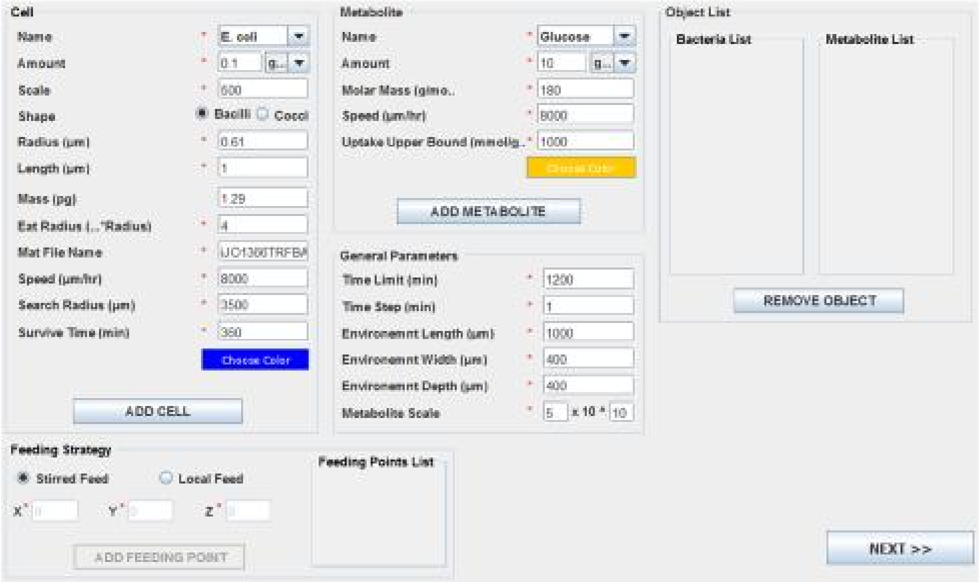

Figure S1. Adding information on cells and metabolites to the panel of ACBM

3.3. Adding metabolites

Enter properties needed, then click add. These properties include the following items:

1. Name
2. Amount: initial amount (you can choose its unit to be g/l or count)
3. Molar mass (g/mol)
4. Speed (µm/hours): metabolite moves at this speed
5. Uptake Upper Bound (mmol/gDCW h): maximum metabolic flux possible for cells when uptaking this type of metabolite
6. Color: color to show this type of metabolites in simulation

For example, the information for glucose is presented in Figure S1.

3.4. Entering General Parameters

Some other parameters are needed to run simulations, that do not depend on the type of cells and metabolites. These parameters include following items:

1. Time Limit (min): simulations continue for this period
2. Time Step (min)
3. Environment dimensions (µm) including length, width, and depth in µm
14. Metabolite Scale: a certain number of a metabolite considered as an object including a package of metabolite.

The default values are presented in Figure S1.

3.5. Determining feeding points

For simulation of heterogeneous feeding, substrates were injected into the environment to study the substrate starvation. So two modes including stirred and local feeding were considered. In the stirred mode, the substrate is completely stirred in the environment. In the local model, the substrate is injected from one or more point(s). In the case of local feeding, you have to enter coordinates of points and add them (see Figure S2). Note that these coordinates must be within environment dimensions.

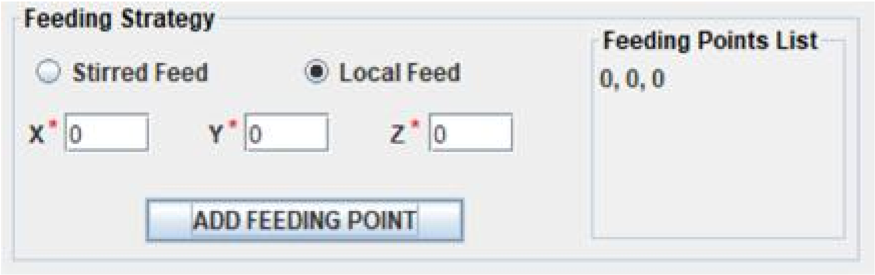

Figure S2. Substrate injection from coordinate (0,0,0).

3.6. Entering data of exchange reactions

Since metabolic models use dissimilar names and directions for uptake reactions of metabolites, entering the data of exchange reactions including their name and direction is required. After adding cell and metabolites to the panel and clicking “NEXT”, a window will appear that contains a table with rows of cells and columns of metabolites. The names and directions should be found from the metabolic model of each cell.

For reaction direction, “1”, “-1” or “N” should be entered. “1” is written if the substrate enters the extracellular environment by the forward direction of exchange reaction. “-1” is written if the substrate enters from the environment to cytoplasm by the forward direction of exchange reaction. Note: if a metabolic model does not have exchange reaction of a substrate, enter “N” in the name cell and “0” in the direction cell.

The data of exchange reactions for metabolic models of *F. prausnitzii* and *B. adolescentis* is presented in Figure S3.

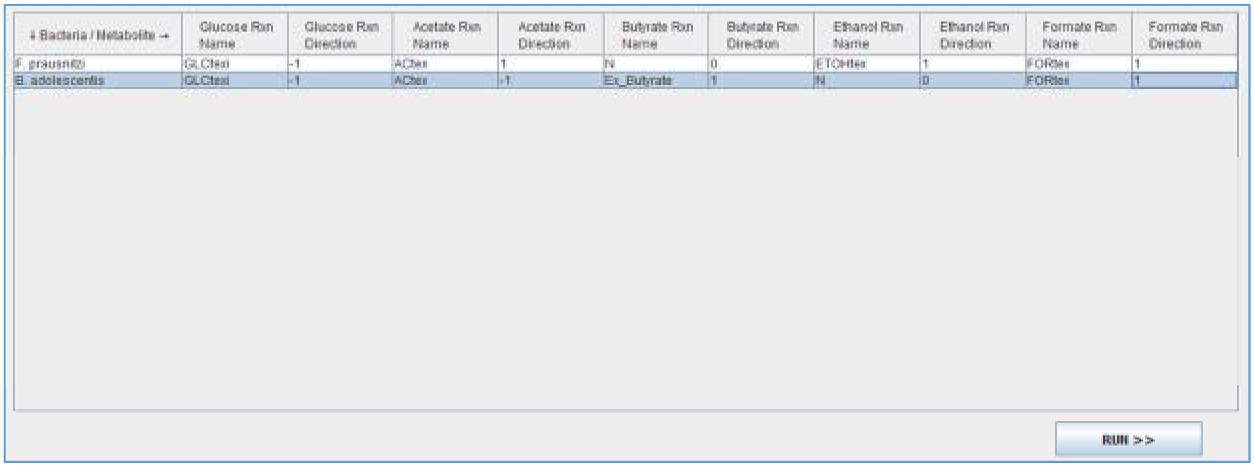

Figure S3. Entering data of exchange reactions for metabolic models of *F. prausnitzii* and *B. adolescentis.*

4. OUTPUT

During the implementation of ACBM, a window appears and shows the visual results which represent the simulated environment in 2D view (Figure S4). Furthermore, time (min) and concentration (g/l) of cells and metabolites is presented in right-hand side of the window.

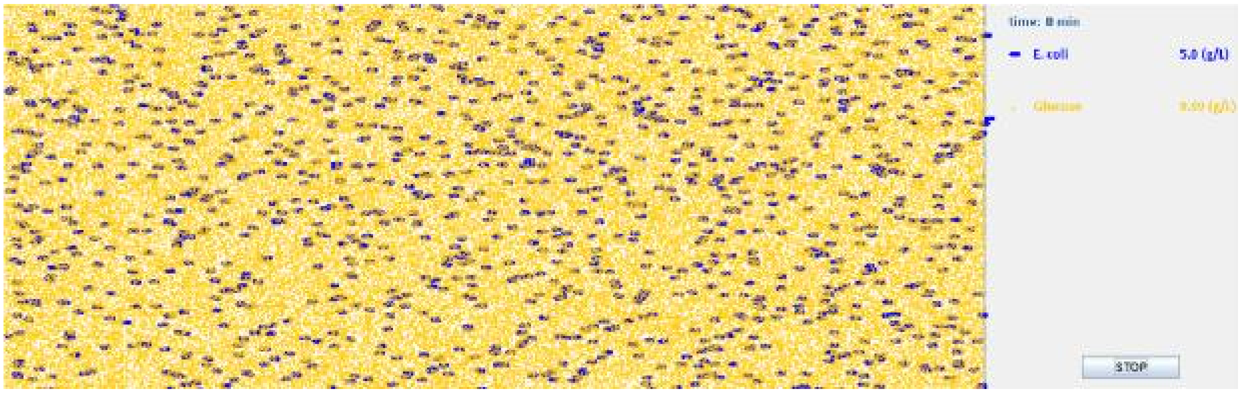

Figure S4. A 2D view of the environment during batch growth of *E. coli*. Time (min) and concentration (g/l) of *E. coli* and glucose are presented in the right-hand side of the window.

After implementation, an output file (output.txt) is generated and can be found in current directory. It shows concentrations and number of metabolites and cells of each type and number of died cells and cells that could not find substrate in time step (Figure S5).

```
Time E. coli_Count E. coli_Conc Glucose_Count Glucose_Conc E. coli_Died bacteria_couldn't_eat
0 12 0.1 76469 10.01 0 0
1 12 0.1 76469 10.01 0 0
2 12 0.14 76279 9.98 0 0
3 12 0.17 76150 9.96 0 0
4 19 0.2 76045 9.95 0 0
5 24 0.24 75870 9.93 0 0
6 24 0.29 75652 9.9 0 0
7 27 0.33 75471 9.88 0 0
8 35 0.38 75268 9.85 0 0
9 40 0.45 74999 9.81 0 0
10 47 0.51 74737 9.78 0 0
```

Figure 5. a sample of output file (column one: Time (h), column two: number of cell individuals, column three: biomass concentration (g/l), column four: number of glucose packages, column five: glucose concentration (g/l), column six: number of died cells in the current time step, column seven: number of cells could not find substrate in the time step.

5. CONTACT

IF you have any questions, contact Ehsan Motamedian at motamedian@modares.ac.ir

Reference

Martínez-Salas E, Martín JA, Vicente M (1981) Relationship of *Escherichia coli* density to growth rate and cell age. *Journal of Bacteriology* 147: 97-100
