# Supplementary material for: ACBM: An Integrated Agent and Constraint Based Modeling Framework for Simulation of Microbial Communities
Source: Sci Rep. 2020 May 26;10:8695. doi: 10.1038/s41598-020-65659-w (PMC7250870; doi:10.1038/s41598-020-65659-w)
Supplement: Supplementary file 2 [file 41598_2020_65659_MOESM2_ESM.zip › ACBM1.4/lib/commons-cli-1.3/apidocs/allclasses-frame.html]

All Classes (Apache Commons CLI 1.3 API)


# All Classes

- AlreadySelectedException
- AmbiguousOptionException
- BasicParser
- CommandLine
- CommandLineParser
- DefaultParser
- GnuParser
- HelpFormatter
- MissingArgumentException
- MissingOptionException
- Option
- Option.Builder
- OptionBuilder
- OptionGroup
- Options
- ParseException
- Parser
- PatternOptionBuilder
- PosixParser
- TypeHandler
- UnrecognizedOptionException
