# Supplementary material for: ACBM: An Integrated Agent and Constraint Based Modeling Framework for Simulation of Microbial Communities
Source: Sci Rep. 2020 May 26;10:8695. doi: 10.1038/s41598-020-65659-w (PMC7250870; doi:10.1038/s41598-020-65659-w)
Supplement: Supplementary file 2 [file 41598_2020_65659_MOESM2_ESM.zip › ACBM1.4/lib/commons-cli-1.3/apidocs/constant-values.html]

Constant Field Values (Apache Commons CLI 1.3 API)


JavaScript is disabled on your browser.


Skip navigation links


- Package
- Class
- Use
- Tree
- Deprecated
- Index
- Help

- Prev
- Next

- Frames
- No Frames

- All Classes

# Constant Field Values

## Contents

- org.apache.\*

## org.apache.\*

- org.apache.commons.cli.HelpFormatter

  | Modifier and Type | Constant Field | Value |
  |  |  |  |
  | --- | --- | --- |
  | `public static final String` | `DEFAULT_ARG_NAME` | `"arg"` |
  | `public static final int` | `DEFAULT_DESC_PAD` | `3` |
  | `public static final int` | `DEFAULT_LEFT_PAD` | `1` |
  | `public static final String` | `DEFAULT_LONG_OPT_PREFIX` | `"--"` |
  | `public static final String` | `DEFAULT_LONG_OPT_SEPARATOR` | `" "` |
  | `public static final String` | `DEFAULT_OPT_PREFIX` | `"-"` |
  | `public static final String` | `DEFAULT_SYNTAX_PREFIX` | `"usage: "` |
  | `public static final int` | `DEFAULT_WIDTH` | `74` |
- org.apache.commons.cli.Option

  | Modifier and Type | Constant Field | Value |
  |  |  |  |
  | --- | --- | --- |
  | `public static final int` | `UNINITIALIZED` | `-1` |
  | `public static final int` | `UNLIMITED_VALUES` | `-2` |

Skip navigation links


- Package
- Class
- Use
- Tree
- Deprecated
- Index
- Help

- Prev
- Next

- Frames
- No Frames

- All Classes

Copyright © 2002–2015 The Apache Software Foundation. All rights reserved.
