# Supplementary material for: ACBM: An Integrated Agent and Constraint Based Modeling Framework for Simulation of Microbial Communities
Source: Sci Rep. 2020 May 26;10:8695. doi: 10.1038/s41598-020-65659-w (PMC7250870; doi:10.1038/s41598-020-65659-w)
Supplement: Supplementary file 2 [file 41598_2020_65659_MOESM2_ESM.zip › ACBM1.4/lib/commons-cli-1.3/apidocs/deprecated-list.html]

Deprecated List (Apache Commons CLI 1.3 API)


JavaScript is disabled on your browser.


Skip navigation links


- Package
- Class
- Use
- Tree
- Deprecated
- Index
- Help

- Prev
- Next

- Frames
- No Frames

- All Classes

# Deprecated API

## Contents

- Deprecated Classes
- Deprecated Fields
- Deprecated Methods

- Deprecated Classes

  | Class and Description |
  |  |
  | --- |
  | org.apache.commons.cli.BasicParser since 1.3, use the `DefaultParser` instead |
  | org.apache.commons.cli.GnuParser since 1.3, use the `DefaultParser` instead |
  | org.apache.commons.cli.OptionBuilder since 1.3, use `Option.builder(String)` instead |
  | org.apache.commons.cli.Parser since 1.3, the two-pass parsing with the flatten method is not enough flexible to handle complex cases |
  | org.apache.commons.cli.PosixParser since 1.3, use the `DefaultParser` instead |


- Deprecated Fields

  | Field and Description |
  |  |
  | --- |
  | org.apache.commons.cli.HelpFormatter.defaultArgName Scope will be made private for next major version - use get/setArgName methods instead. |
  | org.apache.commons.cli.HelpFormatter.defaultDescPad Scope will be made private for next major version - use get/setDescPadding methods instead. |
  | org.apache.commons.cli.HelpFormatter.defaultLeftPad Scope will be made private for next major version - use get/setLeftPadding methods instead. |
  | org.apache.commons.cli.HelpFormatter.defaultLongOptPrefix Scope will be made private for next major version - use get/setLongOptPrefix methods instead. |
  | org.apache.commons.cli.HelpFormatter.defaultNewLine Scope will be made private for next major version - use get/setNewLine methods instead. |
  | org.apache.commons.cli.HelpFormatter.defaultOptPrefix Scope will be made private for next major version - use get/setOptPrefix methods instead. |
  | org.apache.commons.cli.HelpFormatter.defaultSyntaxPrefix Scope will be made private for next major version - use get/setSyntaxPrefix methods instead. |
  | org.apache.commons.cli.HelpFormatter.defaultWidth Scope will be made private for next major version - use get/setWidth methods instead. |


- Deprecated Methods

  | Method and Description |
  |  |
  | --- |
  | org.apache.commons.cli.Option.addValue(String) |
  | org.apache.commons.cli.CommandLine.getOptionObject(String) due to System.err message. Instead use getParsedOptionValue(String) |
  | org.apache.commons.cli.Option.setType(Object) since 1.3, use `Option.setType(Class)` instead |
  | org.apache.commons.cli.OptionBuilder.withType(Object) since 1.3, use `OptionBuilder.withType(Class)` instead |

Skip navigation links


- Package
- Class
- Use
- Tree
- Deprecated
- Index
- Help

- Prev
- Next

- Frames
- No Frames

- All Classes

Copyright © 2002–2015 The Apache Software Foundation. All rights reserved.
