# Supplementary material for: ACBM: An Integrated Agent and Constraint Based Modeling Framework for Simulation of Microbial Communities
Source: Sci Rep. 2020 May 26;10:8695. doi: 10.1038/s41598-020-65659-w (PMC7250870; doi:10.1038/s41598-020-65659-w)
Supplement: Supplementary file 2 [file 41598_2020_65659_MOESM2_ESM.zip › ACBM1.4/lib/commons-cli-1.3/apidocs/index-all.html]

Index (Apache Commons CLI 1.3 API)


JavaScript is disabled on your browser.


Skip navigation links


- Package
- Class
- Use
- Tree
- Deprecated
- Index
- Help

- Prev
- Next

- Frames
- No Frames

- All Classes

A B C D E F G H I L M N O P R S T U V W 


## A

addArg(String) - Method in class org.apache.commons.cli.CommandLine
:   Add left-over unrecognized option/argument.

addOption(Option) - Method in class org.apache.commons.cli.CommandLine
:   Add an option to the command line.

addOption(Option) - Method in class org.apache.commons.cli.OptionGroup
:   Add the specified `Option` to this group.

addOption(String, String) - Method in class org.apache.commons.cli.Options
:   Add an option that only contains a short name.

addOption(String, boolean, String) - Method in class org.apache.commons.cli.Options
:   Add an option that only contains a short-name.

addOption(String, String, boolean, String) - Method in class org.apache.commons.cli.Options
:   Add an option that contains a short-name and a long-name.

addOption(Option) - Method in class org.apache.commons.cli.Options
:   Adds an option instance

addOptionGroup(OptionGroup) - Method in class org.apache.commons.cli.Options
:   Add the specified option group.

addValue(String) - Method in class org.apache.commons.cli.Option
:   Deprecated.

AlreadySelectedException - Exception in org.apache.commons.cli
:   Thrown when more than one option in an option group
    has been provided.

AlreadySelectedException(String) - Constructor for exception org.apache.commons.cli.AlreadySelectedException
:   Construct a new `AlreadySelectedException`
    with the specified detail message.

AlreadySelectedException(OptionGroup, Option) - Constructor for exception org.apache.commons.cli.AlreadySelectedException
:   Construct a new `AlreadySelectedException`
    for the specified option group.

AmbiguousOptionException - Exception in org.apache.commons.cli
:   Exception thrown when an option can't be identified from a partial name.

AmbiguousOptionException(String, Collection<String>) - Constructor for exception org.apache.commons.cli.AmbiguousOptionException
:   Constructs a new AmbiguousOptionException.

argName(String) - Method in class org.apache.commons.cli.Option.Builder
:   Sets the display name for the argument value.


## B

BasicParser - Class in org.apache.commons.cli
:   Deprecated.

    since 1.3, use the `DefaultParser` instead

BasicParser() - Constructor for class org.apache.commons.cli.BasicParser
:   Deprecated.

build() - Method in class org.apache.commons.cli.Option.Builder
:   Constructs an Option with the values declared by this `Option.Builder`.

builder() - Static method in class org.apache.commons.cli.Option
:   Returns a `Option.Builder` to create an `Option` using descriptive
    methods.

builder(String) - Static method in class org.apache.commons.cli.Option
:   Returns a `Option.Builder` to create an `Option` using descriptive
    methods.

burstToken(String, boolean) - Method in class org.apache.commons.cli.PosixParser
:   Deprecated.

    Breaks `token` into its constituent parts
    using the following algorithm.


## C

checkRequiredOptions() - Method in class org.apache.commons.cli.Parser
:   Deprecated.

    Throws a `MissingOptionException` if all of the required options
    are not present.

CLASS\_VALUE - Static variable in class org.apache.commons.cli.PatternOptionBuilder
:   Class class

clone() - Method in class org.apache.commons.cli.Option
:   A rather odd clone method - due to incorrect code in 1.0 it is public
    and in 1.1 rather than throwing a CloneNotSupportedException it throws
    a RuntimeException so as to maintain backwards compat at the API level.

cmd - Variable in class org.apache.commons.cli.DefaultParser
:   The command-line instance.

cmd - Variable in class org.apache.commons.cli.Parser
:   Deprecated.

    commandline instance

CommandLine - Class in org.apache.commons.cli
:   Represents list of arguments parsed against a `Options` descriptor.

CommandLine() - Constructor for class org.apache.commons.cli.CommandLine
:   Creates a command line.

CommandLineParser - Interface in org.apache.commons.cli
:   A class that implements the `CommandLineParser` interface
    can parse a String array according to the `Options` specified
    and return a `CommandLine`.

create(char) - Static method in class org.apache.commons.cli.OptionBuilder
:   Deprecated.

    Create an Option using the current settings and with
    the specified Option `char`.

create() - Static method in class org.apache.commons.cli.OptionBuilder
:   Deprecated.

    Create an Option using the current settings

create(String) - Static method in class org.apache.commons.cli.OptionBuilder
:   Deprecated.

    Create an Option using the current settings and with
    the specified Option `char`.

createClass(String) - Static method in class org.apache.commons.cli.TypeHandler
:   Returns the class whose name is `classname`.

createDate(String) - Static method in class org.apache.commons.cli.TypeHandler
:   Returns the date represented by `str`.

createFile(String) - Static method in class org.apache.commons.cli.TypeHandler
:   Returns the File represented by `str`.

createFiles(String) - Static method in class org.apache.commons.cli.TypeHandler
:   Returns the File[] represented by `str`.

createNumber(String) - Static method in class org.apache.commons.cli.TypeHandler
:   Create a number from a String.

createObject(String) - Static method in class org.apache.commons.cli.TypeHandler
:   Create an Object from the classname and empty constructor.

createPadding(int) - Method in class org.apache.commons.cli.HelpFormatter
:   Return a String of padding of length `len`.

createURL(String) - Static method in class org.apache.commons.cli.TypeHandler
:   Returns the URL represented by `str`.

createValue(String, Object) - Static method in class org.apache.commons.cli.TypeHandler
:   Returns the `Object` of type `obj`
    with the value of `str`.

createValue(String, Class<?>) - Static method in class org.apache.commons.cli.TypeHandler
:   Returns the `Object` of type `clazz`
    with the value of `str`.

currentOption - Variable in class org.apache.commons.cli.DefaultParser
:   The last option parsed.

currentToken - Variable in class org.apache.commons.cli.DefaultParser
:   The token currently processed.


## D

DATE\_VALUE - Static variable in class org.apache.commons.cli.PatternOptionBuilder
:   Date class

DEFAULT\_ARG\_NAME - Static variable in class org.apache.commons.cli.HelpFormatter
:   default name for an argument

DEFAULT\_DESC\_PAD - Static variable in class org.apache.commons.cli.HelpFormatter
:   number of space characters to be prefixed to each description line

DEFAULT\_LEFT\_PAD - Static variable in class org.apache.commons.cli.HelpFormatter
:   default padding to the left of each line

DEFAULT\_LONG\_OPT\_PREFIX - Static variable in class org.apache.commons.cli.HelpFormatter
:   default prefix for long Option

DEFAULT\_LONG\_OPT\_SEPARATOR - Static variable in class org.apache.commons.cli.HelpFormatter
:   default separator displayed between a long Option and its value

DEFAULT\_OPT\_PREFIX - Static variable in class org.apache.commons.cli.HelpFormatter
:   default prefix for shortOpts

DEFAULT\_SYNTAX\_PREFIX - Static variable in class org.apache.commons.cli.HelpFormatter
:   the string to display at the beginning of the usage statement

DEFAULT\_WIDTH - Static variable in class org.apache.commons.cli.HelpFormatter
:   default number of characters per line

defaultArgName - Variable in class org.apache.commons.cli.HelpFormatter
:   Deprecated.

    Scope will be made private for next major version
    - use get/setArgName methods instead.

defaultDescPad - Variable in class org.apache.commons.cli.HelpFormatter
:   Deprecated.

    Scope will be made private for next major version
    - use get/setDescPadding methods instead.

defaultLeftPad - Variable in class org.apache.commons.cli.HelpFormatter
:   Deprecated.

    Scope will be made private for next major version
    - use get/setLeftPadding methods instead.

defaultLongOptPrefix - Variable in class org.apache.commons.cli.HelpFormatter
:   Deprecated.

    Scope will be made private for next major version
    - use get/setLongOptPrefix methods instead.

defaultNewLine - Variable in class org.apache.commons.cli.HelpFormatter
:   Deprecated.

    Scope will be made private for next major version
    - use get/setNewLine methods instead.

defaultOptPrefix - Variable in class org.apache.commons.cli.HelpFormatter
:   Deprecated.

    Scope will be made private for next major version
    - use get/setOptPrefix methods instead.

DefaultParser - Class in org.apache.commons.cli
:   Default parser.

DefaultParser() - Constructor for class org.apache.commons.cli.DefaultParser


defaultSyntaxPrefix - Variable in class org.apache.commons.cli.HelpFormatter
:   Deprecated.

    Scope will be made private for next major version
    - use get/setSyntaxPrefix methods instead.

defaultWidth - Variable in class org.apache.commons.cli.HelpFormatter
:   Deprecated.

    Scope will be made private for next major version
    - use get/setWidth methods instead.

desc(String) - Method in class org.apache.commons.cli.Option.Builder
:   Sets the description for this option.


## E

equals(Object) - Method in class org.apache.commons.cli.Option


EXISTING\_FILE\_VALUE - Static variable in class org.apache.commons.cli.PatternOptionBuilder
:   FileInputStream class

expectedOpts - Variable in class org.apache.commons.cli.DefaultParser
:   The required options and groups expected to be found when parsing the command line.


## F

FILE\_VALUE - Static variable in class org.apache.commons.cli.PatternOptionBuilder
:   File class

FILES\_VALUE - Static variable in class org.apache.commons.cli.PatternOptionBuilder
:   File array class

findWrapPos(String, int, int) - Method in class org.apache.commons.cli.HelpFormatter
:   Finds the next text wrap position after `startPos` for the
    text in `text` with the column width `width`.

flatten(Options, String[], boolean) - Method in class org.apache.commons.cli.BasicParser
:   Deprecated.

    A simple implementation of `Parser`'s abstract
    `flatten` method.

flatten(Options, String[], boolean) - Method in class org.apache.commons.cli.GnuParser
:   Deprecated.

    This flatten method does so using the following rules:
    If an `Option` exists for the first character of
    the `arguments` entry **AND** an `Option`
    does not exist for the whole `argument` then
    add the first character as an option to the processed tokens
    list e.g.

flatten(Options, String[], boolean) - Method in class org.apache.commons.cli.Parser
:   Deprecated.

    Subclasses must implement this method to reduce
    the `arguments` that have been passed to the parse method.

flatten(Options, String[], boolean) - Method in class org.apache.commons.cli.PosixParser
:   Deprecated.

    An implementation of `Parser`'s abstract
    `flatten` method.


## G

getArgList() - Method in class org.apache.commons.cli.CommandLine
:   Retrieve any left-over non-recognized options and arguments

getArgName() - Method in class org.apache.commons.cli.HelpFormatter
:   Returns the 'argName'.

getArgName() - Method in class org.apache.commons.cli.Option
:   Gets the display name for the argument value.

getArgs() - Method in class org.apache.commons.cli.CommandLine
:   Retrieve any left-over non-recognized options and arguments

getArgs() - Method in class org.apache.commons.cli.Option
:   Returns the number of argument values this Option can take.

getDescPadding() - Method in class org.apache.commons.cli.HelpFormatter
:   Returns the 'descPadding'.

getDescription() - Method in class org.apache.commons.cli.Option
:   Retrieve the self-documenting description of this Option

getId() - Method in class org.apache.commons.cli.Option
:   Returns the id of this Option.

getLeftPadding() - Method in class org.apache.commons.cli.HelpFormatter
:   Returns the 'leftPadding'.

getLongOpt() - Method in class org.apache.commons.cli.Option
:   Retrieve the long name of this Option.

getLongOptPrefix() - Method in class org.apache.commons.cli.HelpFormatter
:   Returns the 'longOptPrefix'.

getLongOptSeparator() - Method in class org.apache.commons.cli.HelpFormatter
:   Returns the separator displayed between a long option and its value.

getMatchingOptions() - Method in exception org.apache.commons.cli.AmbiguousOptionException
:   Returns the options matching the partial name.

getMatchingOptions(String) - Method in class org.apache.commons.cli.Options
:   Returns the options with a long name starting with the name specified.

getMissingOptions() - Method in exception org.apache.commons.cli.MissingOptionException
:   Returns the list of options or option groups missing in the command line parsed.

getNames() - Method in class org.apache.commons.cli.OptionGroup


getNewLine() - Method in class org.apache.commons.cli.HelpFormatter
:   Returns the 'newLine'.

getOpt() - Method in class org.apache.commons.cli.Option
:   Retrieve the name of this Option.

getOption() - Method in exception org.apache.commons.cli.AlreadySelectedException
:   Returns the option that was added to the group and triggered the exception.

getOption() - Method in exception org.apache.commons.cli.MissingArgumentException
:   Return the option requiring an argument that wasn't provided
    on the command line.

getOption(String) - Method in class org.apache.commons.cli.Options
:   Retrieve the `Option` matching the long or short name specified.

getOption() - Method in exception org.apache.commons.cli.UnrecognizedOptionException
:   Returns the unrecognized option.

getOptionComparator() - Method in class org.apache.commons.cli.HelpFormatter
:   Comparator used to sort the options when they output in help text.

getOptionGroup() - Method in exception org.apache.commons.cli.AlreadySelectedException
:   Returns the option group where another option has been selected.

getOptionGroup(Option) - Method in class org.apache.commons.cli.Options
:   Returns the OptionGroup the `opt` belongs to.

getOptionObject(String) - Method in class org.apache.commons.cli.CommandLine
:   Deprecated.

    due to System.err message. Instead use getParsedOptionValue(String)

getOptionObject(char) - Method in class org.apache.commons.cli.CommandLine
:   Return the `Object` type of this `Option`.

getOptionProperties(String) - Method in class org.apache.commons.cli.CommandLine
:   Retrieve the map of values associated to the option.

getOptions() - Method in class org.apache.commons.cli.CommandLine
:   Returns an array of the processed `Option`s.

getOptions() - Method in class org.apache.commons.cli.OptionGroup


getOptions() - Method in class org.apache.commons.cli.Options
:   Retrieve a read-only list of options in this set

getOptions() - Method in class org.apache.commons.cli.Parser
:   Deprecated.

getOptionValue(String) - Method in class org.apache.commons.cli.CommandLine
:   Retrieve the first argument, if any, of this option.

getOptionValue(char) - Method in class org.apache.commons.cli.CommandLine
:   Retrieve the first argument, if any, of this option.

getOptionValue(String, String) - Method in class org.apache.commons.cli.CommandLine
:   Retrieve the first argument, if any, of an option.

getOptionValue(char, String) - Method in class org.apache.commons.cli.CommandLine
:   Retrieve the argument, if any, of an option.

getOptionValues(String) - Method in class org.apache.commons.cli.CommandLine
:   Retrieves the array of values, if any, of an option.

getOptionValues(char) - Method in class org.apache.commons.cli.CommandLine
:   Retrieves the array of values, if any, of an option.

getOptPrefix() - Method in class org.apache.commons.cli.HelpFormatter
:   Returns the 'optPrefix'.

getParsedOptionValue(String) - Method in class org.apache.commons.cli.CommandLine
:   Return a version of this `Option` converted to a particular type.

getRequiredOptions() - Method in class org.apache.commons.cli.Options
:   Returns the required options.

getRequiredOptions() - Method in class org.apache.commons.cli.Parser
:   Deprecated.

getSelected() - Method in class org.apache.commons.cli.OptionGroup


getSyntaxPrefix() - Method in class org.apache.commons.cli.HelpFormatter
:   Returns the 'syntaxPrefix'.

getType() - Method in class org.apache.commons.cli.Option
:   Retrieve the type of this Option.

getValue() - Method in class org.apache.commons.cli.Option
:   Returns the specified value of this Option or
    `null` if there is no value.

getValue(int) - Method in class org.apache.commons.cli.Option
:   Returns the specified value of this Option or
    `null` if there is no value.

getValue(String) - Method in class org.apache.commons.cli.Option
:   Returns the value/first value of this Option or the
    `defaultValue` if there is no value.

getValueClass(char) - Static method in class org.apache.commons.cli.PatternOptionBuilder
:   Retrieve the class that `ch` represents.

getValues() - Method in class org.apache.commons.cli.Option
:   Return the values of this Option as a String array
    or null if there are no values

getValueSeparator() - Method in class org.apache.commons.cli.Option
:   Returns the value separator character.

getValuesList() - Method in class org.apache.commons.cli.Option


getWidth() - Method in class org.apache.commons.cli.HelpFormatter
:   Returns the 'width'.

GnuParser - Class in org.apache.commons.cli
:   Deprecated.

    since 1.3, use the `DefaultParser` instead

GnuParser() - Constructor for class org.apache.commons.cli.GnuParser
:   Deprecated.


## H

handleConcatenatedOptions(String) - Method in class org.apache.commons.cli.DefaultParser
:   Breaks `token` into its constituent parts
    using the following algorithm.

hasArg() - Method in class org.apache.commons.cli.Option.Builder
:   Indicates that the Option will require an argument.

hasArg(boolean) - Method in class org.apache.commons.cli.Option.Builder
:   Indicates if the Option has an argument or not.

hasArg() - Method in class org.apache.commons.cli.Option
:   Query to see if this Option requires an argument

hasArg() - Static method in class org.apache.commons.cli.OptionBuilder
:   Deprecated.

    The next Option created will require an argument value.

hasArg(boolean) - Static method in class org.apache.commons.cli.OptionBuilder
:   Deprecated.

    The next Option created will require an argument value if
    `hasArg` is true.

hasArgName() - Method in class org.apache.commons.cli.Option
:   Returns whether the display name for the argument value has been set.

hasArgs() - Method in class org.apache.commons.cli.Option.Builder
:   Indicates that the Option can have unlimited argument values.

hasArgs() - Method in class org.apache.commons.cli.Option
:   Query to see if this Option can take many values.

hasArgs() - Static method in class org.apache.commons.cli.OptionBuilder
:   Deprecated.

    The next Option created can have unlimited argument values.

hasArgs(int) - Static method in class org.apache.commons.cli.OptionBuilder
:   Deprecated.

    The next Option created can have `num` argument values.

hashCode() - Method in class org.apache.commons.cli.Option


hasLongOpt() - Method in class org.apache.commons.cli.Option
:   Query to see if this Option has a long name

hasLongOption(String) - Method in class org.apache.commons.cli.Options
:   Returns whether the named `Option` is a member of this `Options`.

hasOption(String) - Method in class org.apache.commons.cli.CommandLine
:   Query to see if an option has been set.

hasOption(char) - Method in class org.apache.commons.cli.CommandLine
:   Query to see if an option has been set.

hasOption(String) - Method in class org.apache.commons.cli.Options
:   Returns whether the named `Option` is a member of this `Options`.

hasOptionalArg() - Method in class org.apache.commons.cli.Option


hasOptionalArg() - Static method in class org.apache.commons.cli.OptionBuilder
:   Deprecated.

    The next Option can have an optional argument.

hasOptionalArgs() - Static method in class org.apache.commons.cli.OptionBuilder
:   Deprecated.

    The next Option can have an unlimited number of optional arguments.

hasOptionalArgs(int) - Static method in class org.apache.commons.cli.OptionBuilder
:   Deprecated.

    The next Option can have the specified number of optional arguments.

hasShortOption(String) - Method in class org.apache.commons.cli.Options
:   Returns whether the named `Option` is a member of this `Options`.

hasValueSeparator() - Method in class org.apache.commons.cli.Option
:   Return whether this Option has specified a value separator.

HelpFormatter - Class in org.apache.commons.cli
:   A formatter of help messages for command line options.

HelpFormatter() - Constructor for class org.apache.commons.cli.HelpFormatter


## I

isRequired() - Method in class org.apache.commons.cli.Option
:   Query to see if this Option is mandatory

isRequired() - Static method in class org.apache.commons.cli.OptionBuilder
:   Deprecated.

    The next Option created will be required.

isRequired(boolean) - Static method in class org.apache.commons.cli.OptionBuilder
:   Deprecated.

    The next Option created will be required if `required`
    is true.

isRequired() - Method in class org.apache.commons.cli.OptionGroup
:   Returns whether this option group is required.

isValueCode(char) - Static method in class org.apache.commons.cli.PatternOptionBuilder
:   Returns whether `ch` is a value code, i.e.

iterator() - Method in class org.apache.commons.cli.CommandLine
:   Returns an iterator over the Option members of CommandLine.


## L

longOpt(String) - Method in class org.apache.commons.cli.Option.Builder
:   Sets the long name of the Option.


## M

MissingArgumentException - Exception in org.apache.commons.cli
:   Thrown when an option requiring an argument
    is not provided with an argument.

MissingArgumentException(String) - Constructor for exception org.apache.commons.cli.MissingArgumentException
:   Construct a new `MissingArgumentException`
    with the specified detail message.

MissingArgumentException(Option) - Constructor for exception org.apache.commons.cli.MissingArgumentException
:   Construct a new `MissingArgumentException`
    with the specified detail message.

MissingOptionException - Exception in org.apache.commons.cli
:   Thrown when a required option has not been provided.

MissingOptionException(String) - Constructor for exception org.apache.commons.cli.MissingOptionException
:   Construct a new `MissingSelectedException`
    with the specified detail message.

MissingOptionException(List) - Constructor for exception org.apache.commons.cli.MissingOptionException
:   Constructs a new `MissingSelectedException` with the
    specified list of missing options.


## N

NUMBER\_VALUE - Static variable in class org.apache.commons.cli.PatternOptionBuilder
:   Number class

numberOfArgs(int) - Method in class org.apache.commons.cli.Option.Builder
:   Sets the number of argument values the Option can take.


## O

OBJECT\_VALUE - Static variable in class org.apache.commons.cli.PatternOptionBuilder
:   Object class

Option - Class in org.apache.commons.cli
:   Describes a single command-line option.

Option(String, String) - Constructor for class org.apache.commons.cli.Option
:   Creates an Option using the specified parameters.

Option(String, boolean, String) - Constructor for class org.apache.commons.cli.Option
:   Creates an Option using the specified parameters.

Option(String, String, boolean, String) - Constructor for class org.apache.commons.cli.Option
:   Creates an Option using the specified parameters.

Option.Builder - Class in org.apache.commons.cli
:   A nested builder class to create `Option` instances
    using descriptive methods.

optionalArg(boolean) - Method in class org.apache.commons.cli.Option.Builder
:   Sets whether the Option can have an optional argument.

OptionBuilder - Class in org.apache.commons.cli
:   Deprecated.

    since 1.3, use `Option.builder(String)` instead

optionComparator - Variable in class org.apache.commons.cli.HelpFormatter
:   Comparator used to sort the options when they output in help text
    Defaults to case-insensitive alphabetical sorting by option key

OptionGroup - Class in org.apache.commons.cli
:   A group of mutually exclusive options.

OptionGroup() - Constructor for class org.apache.commons.cli.OptionGroup


options - Variable in class org.apache.commons.cli.DefaultParser
:   The current options.

Options - Class in org.apache.commons.cli
:   Main entry-point into the library.

Options() - Constructor for class org.apache.commons.cli.Options


org.apache.commons.cli - package org.apache.commons.cli
:   Commons CLI 1.3


## P

parse(Options, String[]) - Method in interface org.apache.commons.cli.CommandLineParser
:   Parse the arguments according to the specified options.

parse(Options, String[], boolean) - Method in interface org.apache.commons.cli.CommandLineParser
:   Parse the arguments according to the specified options.

parse(Options, String[]) - Method in class org.apache.commons.cli.DefaultParser


parse(Options, String[], Properties) - Method in class org.apache.commons.cli.DefaultParser
:   Parse the arguments according to the specified options and properties.

parse(Options, String[], boolean) - Method in class org.apache.commons.cli.DefaultParser


parse(Options, String[], Properties, boolean) - Method in class org.apache.commons.cli.DefaultParser
:   Parse the arguments according to the specified options and properties.

parse(Options, String[]) - Method in class org.apache.commons.cli.Parser
:   Deprecated.

    Parses the specified `arguments` based
    on the specified `Options`.

parse(Options, String[], Properties) - Method in class org.apache.commons.cli.Parser
:   Deprecated.

    Parse the arguments according to the specified options and properties.

parse(Options, String[], boolean) - Method in class org.apache.commons.cli.Parser
:   Deprecated.

    Parses the specified `arguments`
    based on the specified `Options`.

parse(Options, String[], Properties, boolean) - Method in class org.apache.commons.cli.Parser
:   Deprecated.

    Parse the arguments according to the specified options and
    properties.

ParseException - Exception in org.apache.commons.cli
:   Base for Exceptions thrown during parsing of a command-line.

ParseException(String) - Constructor for exception org.apache.commons.cli.ParseException
:   Construct a new `ParseException`
    with the specified detail message.

parsePattern(String) - Static method in class org.apache.commons.cli.PatternOptionBuilder
:   Returns the `Options` instance represented by `pattern`.

Parser - Class in org.apache.commons.cli
:   Deprecated.

    since 1.3, the two-pass parsing with the flatten method is not enough flexible to handle complex cases

Parser() - Constructor for class org.apache.commons.cli.Parser
:   Deprecated.

PatternOptionBuilder - Class in org.apache.commons.cli
:   Allows Options to be created from a single String.

PatternOptionBuilder() - Constructor for class org.apache.commons.cli.PatternOptionBuilder


PosixParser - Class in org.apache.commons.cli
:   Deprecated.

    since 1.3, use the `DefaultParser` instead

PosixParser() - Constructor for class org.apache.commons.cli.PosixParser
:   Deprecated.

printHelp(String, Options) - Method in class org.apache.commons.cli.HelpFormatter
:   Print the help for `options` with the specified
    command line syntax.

printHelp(String, Options, boolean) - Method in class org.apache.commons.cli.HelpFormatter
:   Print the help for `options` with the specified
    command line syntax.

printHelp(String, String, Options, String) - Method in class org.apache.commons.cli.HelpFormatter
:   Print the help for `options` with the specified
    command line syntax.

printHelp(String, String, Options, String, boolean) - Method in class org.apache.commons.cli.HelpFormatter
:   Print the help for `options` with the specified
    command line syntax.

printHelp(int, String, String, Options, String) - Method in class org.apache.commons.cli.HelpFormatter
:   Print the help for `options` with the specified
    command line syntax.

printHelp(int, String, String, Options, String, boolean) - Method in class org.apache.commons.cli.HelpFormatter
:   Print the help for `options` with the specified
    command line syntax.

printHelp(PrintWriter, int, String, String, Options, int, int, String) - Method in class org.apache.commons.cli.HelpFormatter
:   Print the help for `options` with the specified
    command line syntax.

printHelp(PrintWriter, int, String, String, Options, int, int, String, boolean) - Method in class org.apache.commons.cli.HelpFormatter
:   Print the help for `options` with the specified
    command line syntax.

printOptions(PrintWriter, int, Options, int, int) - Method in class org.apache.commons.cli.HelpFormatter
:   Print the help for the specified Options to the specified writer,
    using the specified width, left padding and description padding.

printUsage(PrintWriter, int, String, Options) - Method in class org.apache.commons.cli.HelpFormatter
:   Prints the usage statement for the specified application.

printUsage(PrintWriter, int, String) - Method in class org.apache.commons.cli.HelpFormatter
:   Print the cmdLineSyntax to the specified writer, using the
    specified width.

printWrapped(PrintWriter, int, String) - Method in class org.apache.commons.cli.HelpFormatter
:   Print the specified text to the specified PrintWriter.

printWrapped(PrintWriter, int, int, String) - Method in class org.apache.commons.cli.HelpFormatter
:   Print the specified text to the specified PrintWriter.

processArgs(Option, ListIterator<String>) - Method in class org.apache.commons.cli.Parser
:   Deprecated.

    Process the argument values for the specified Option
    `opt` using the values retrieved from the
    specified iterator `iter`.

processOption(String, ListIterator<String>) - Method in class org.apache.commons.cli.Parser
:   Deprecated.

    Process the Option specified by `arg` using the values
    retrieved from the specified iterator `iter`.

processProperties(Properties) - Method in class org.apache.commons.cli.Parser
:   Deprecated.

    Sets the values of Options using the values in `properties`.


## R

renderOptions(StringBuffer, int, Options, int, int) - Method in class org.apache.commons.cli.HelpFormatter
:   Render the specified Options and return the rendered Options
    in a StringBuffer.

renderWrappedText(StringBuffer, int, int, String) - Method in class org.apache.commons.cli.HelpFormatter
:   Render the specified text and return the rendered Options
    in a StringBuffer.

required() - Method in class org.apache.commons.cli.Option.Builder
:   Marks this Option as required.

required(boolean) - Method in class org.apache.commons.cli.Option.Builder
:   Sets whether the Option is mandatory.

rtrim(String) - Method in class org.apache.commons.cli.HelpFormatter
:   Remove the trailing whitespace from the specified String.


## S

setArgName(String) - Method in class org.apache.commons.cli.HelpFormatter
:   Sets the 'argName'.

setArgName(String) - Method in class org.apache.commons.cli.Option
:   Sets the display name for the argument value.

setArgs(int) - Method in class org.apache.commons.cli.Option
:   Sets the number of argument values this Option can take.

setDescPadding(int) - Method in class org.apache.commons.cli.HelpFormatter
:   Sets the 'descPadding'.

setDescription(String) - Method in class org.apache.commons.cli.Option
:   Sets the self-documenting description of this Option

setLeftPadding(int) - Method in class org.apache.commons.cli.HelpFormatter
:   Sets the 'leftPadding'.

setLongOpt(String) - Method in class org.apache.commons.cli.Option
:   Sets the long name of this Option.

setLongOptPrefix(String) - Method in class org.apache.commons.cli.HelpFormatter
:   Sets the 'longOptPrefix'.

setLongOptSeparator(String) - Method in class org.apache.commons.cli.HelpFormatter
:   Set the separator displayed between a long option and its value.

setNewLine(String) - Method in class org.apache.commons.cli.HelpFormatter
:   Sets the 'newLine'.

setOptionalArg(boolean) - Method in class org.apache.commons.cli.Option
:   Sets whether this Option can have an optional argument.

setOptionComparator(Comparator<Option>) - Method in class org.apache.commons.cli.HelpFormatter
:   Set the comparator used to sort the options when they output in help text.

setOptions(Options) - Method in class org.apache.commons.cli.Parser
:   Deprecated.

setOptPrefix(String) - Method in class org.apache.commons.cli.HelpFormatter
:   Sets the 'optPrefix'.

setRequired(boolean) - Method in class org.apache.commons.cli.Option
:   Sets whether this Option is mandatory.

setRequired(boolean) - Method in class org.apache.commons.cli.OptionGroup


setSelected(Option) - Method in class org.apache.commons.cli.OptionGroup
:   Set the selected option of this group to `name`.

setSyntaxPrefix(String) - Method in class org.apache.commons.cli.HelpFormatter
:   Sets the 'syntaxPrefix'.

setType(Object) - Method in class org.apache.commons.cli.Option
:   Deprecated.

    since 1.3, use `Option.setType(Class)` instead

setType(Class<?>) - Method in class org.apache.commons.cli.Option
:   Sets the type of this Option.

setValueSeparator(char) - Method in class org.apache.commons.cli.Option
:   Sets the value separator.

setWidth(int) - Method in class org.apache.commons.cli.HelpFormatter
:   Sets the 'width'.

skipParsing - Variable in class org.apache.commons.cli.DefaultParser
:   Flag indicating if tokens should no longer be analysed and simply added as arguments of the command line.

stopAtNonOption - Variable in class org.apache.commons.cli.DefaultParser
:   Flag indicating how unrecognized tokens are handled.

STRING\_VALUE - Static variable in class org.apache.commons.cli.PatternOptionBuilder
:   String class


## T

toString() - Method in class org.apache.commons.cli.Option
:   Dump state, suitable for debugging.

toString() - Method in class org.apache.commons.cli.OptionGroup
:   Returns the stringified version of this OptionGroup.

toString() - Method in class org.apache.commons.cli.Options
:   Dump state, suitable for debugging.

type(Class<?>) - Method in class org.apache.commons.cli.Option.Builder
:   Sets the type of the Option.

TypeHandler - Class in org.apache.commons.cli
:   This is a temporary implementation.

TypeHandler() - Constructor for class org.apache.commons.cli.TypeHandler


## U

UNINITIALIZED - Static variable in class org.apache.commons.cli.Option
:   constant that specifies the number of argument values has not been specified

UNLIMITED\_VALUES - Static variable in class org.apache.commons.cli.Option
:   constant that specifies the number of argument values is infinite

UnrecognizedOptionException - Exception in org.apache.commons.cli
:   Exception thrown during parsing signalling an unrecognized
    option was seen.

UnrecognizedOptionException(String) - Constructor for exception org.apache.commons.cli.UnrecognizedOptionException
:   Construct a new `UnrecognizedArgumentException`
    with the specified detail message.

UnrecognizedOptionException(String, String) - Constructor for exception org.apache.commons.cli.UnrecognizedOptionException
:   Construct a new `UnrecognizedArgumentException`
    with the specified option and detail message.

URL\_VALUE - Static variable in class org.apache.commons.cli.PatternOptionBuilder
:   URL class


## V

valueSeparator() - Method in class org.apache.commons.cli.Option.Builder
:   The Option will use '=' as a means to separate argument value.

valueSeparator(char) - Method in class org.apache.commons.cli.Option.Builder
:   The Option will use `sep` as a means to
    separate argument values.


## W

withArgName(String) - Static method in class org.apache.commons.cli.OptionBuilder
:   Deprecated.

    The next Option created will have the specified argument value name.

withDescription(String) - Static method in class org.apache.commons.cli.OptionBuilder
:   Deprecated.

    The next Option created will have the specified description

withLongOpt(String) - Static method in class org.apache.commons.cli.OptionBuilder
:   Deprecated.

    The next Option created will have the following long option value.

withType(Object) - Static method in class org.apache.commons.cli.OptionBuilder
:   Deprecated.

    since 1.3, use `OptionBuilder.withType(Class)` instead

withType(Class<?>) - Static method in class org.apache.commons.cli.OptionBuilder
:   Deprecated.

    The next Option created will have a value that will be an instance
    of `type`.

withValueSeparator(char) - Static method in class org.apache.commons.cli.OptionBuilder
:   Deprecated.

    The next Option created uses `sep` as a means to
    separate argument values.

withValueSeparator() - Static method in class org.apache.commons.cli.OptionBuilder
:   Deprecated.

    The next Option created uses '`=`' as a means to
    separate argument values.

A B C D E F G H I L M N O P R S T U V W

Skip navigation links


- Package
- Class
- Use
- Tree
- Deprecated
- Index
- Help

- Prev
- Next

- Frames
- No Frames

- All Classes

Copyright © 2002–2015 The Apache Software Foundation. All rights reserved.
