# Supplementary material for: ACBM: An Integrated Agent and Constraint Based Modeling Framework for Simulation of Microbial Communities
Source: Sci Rep. 2020 May 26;10:8695. doi: 10.1038/s41598-020-65659-w (PMC7250870; doi:10.1038/s41598-020-65659-w)
Supplement: Supplementary file 2 [file 41598_2020_65659_MOESM2_ESM.zip › ACBM1.4/lib/commons-cli-1.3/apidocs/org/apache/commons/cli/AlreadySelectedException.html]

AlreadySelectedException (Apache Commons CLI 1.3 API)


JavaScript is disabled on your browser.


Skip navigation links


- Package
- Class
- Use
- Tree
- Deprecated
- Index
- Help

- Prev Class
- Next Class

- Frames
- No Frames

- All Classes

- Summary:
- Nested |
- Field |
- Constr |
- Method

- Detail:
- Field |
- Constr |
- Method


org.apache.commons.cli

## Class AlreadySelectedException

- java.lang.Object
- - java.lang.Throwable
  - - java.lang.Exception
    - - org.apache.commons.cli.ParseException
      - - org.apache.commons.cli.AlreadySelectedException

- All Implemented Interfaces:
  :   Serializable

  ---

    

  ```
  public class AlreadySelectedException
  extends ParseException
  ```

  Thrown when more than one option in an option group
  has been provided.

  Version:
  :   $Id: AlreadySelectedException.java 1443102 2013-02-06 18:12:16Z tn $

  See Also:
  :   Serialized Form

- - ### Constructor Summary

    Constructors

    | Constructor and Description |
    | `AlreadySelectedException(OptionGroup group, Option option)` Construct a new `AlreadySelectedException` for the specified option group. |
    | `AlreadySelectedException(String message)` Construct a new `AlreadySelectedException` with the specified detail message. |
  - ### Method Summary

    All Methods Instance Methods Concrete Methods

    | Modifier and Type | Method and Description |
    | `Option` | `getOption()` Returns the option that was added to the group and triggered the exception. |
    | `OptionGroup` | `getOptionGroup()` Returns the option group where another option has been selected. |

    - ### Methods inherited from class java.lang.Throwable

      `addSuppressed, fillInStackTrace, getCause, getLocalizedMessage, getMessage, getStackTrace, getSuppressed, initCause, printStackTrace, printStackTrace, printStackTrace, setStackTrace, toString`
    - ### Methods inherited from class java.lang.Object

      `clone, equals, finalize, getClass, hashCode, notify, notifyAll, wait, wait, wait`

- - ### Constructor Detail


    - #### AlreadySelectedException

      ```
      public AlreadySelectedException(String message)
      ```

      Construct a new `AlreadySelectedException`
      with the specified detail message.

      Parameters:
      :   `message` - the detail message


    - #### AlreadySelectedException

      ```
      public AlreadySelectedException(OptionGroup group,
                                      Option option)
      ```

      Construct a new `AlreadySelectedException`
      for the specified option group.

      Parameters:
      :   `group` - the option group already selected
      :   `option` - the option that triggered the exception

      Since:
      :   1.2
  - ### Method Detail


    - #### getOptionGroup

      ```
      public OptionGroup getOptionGroup()
      ```

      Returns the option group where another option has been selected.

      Returns:
      :   the related option group

      Since:
      :   1.2


    - #### getOption

      ```
      public Option getOption()
      ```

      Returns the option that was added to the group and triggered the exception.

      Returns:
      :   the related option

      Since:
      :   1.2


Skip navigation links


- Package
- Class
- Use
- Tree
- Deprecated
- Index
- Help

- Prev Class
- Next Class

- Frames
- No Frames

- All Classes

- Summary:
- Nested |
- Field |
- Constr |
- Method

- Detail:
- Field |
- Constr |
- Method

Copyright © 2002–2015 The Apache Software Foundation. All rights reserved.
