# Supplementary material for: ACBM: An Integrated Agent and Constraint Based Modeling Framework for Simulation of Microbial Communities
Source: Sci Rep. 2020 May 26;10:8695. doi: 10.1038/s41598-020-65659-w (PMC7250870; doi:10.1038/s41598-020-65659-w)
Supplement: Supplementary file 2 [file 41598_2020_65659_MOESM2_ESM.zip › ACBM1.4/lib/commons-cli-1.3/apidocs/org/apache/commons/cli/AmbiguousOptionException.html]

AmbiguousOptionException (Apache Commons CLI 1.3 API)


JavaScript is disabled on your browser.


Skip navigation links


- Package
- Class
- Use
- Tree
- Deprecated
- Index
- Help

- Prev Class
- Next Class

- Frames
- No Frames

- All Classes

- Summary:
- Nested |
- Field |
- Constr |
- Method

- Detail:
- Field |
- Constr |
- Method


org.apache.commons.cli

## Class AmbiguousOptionException

- java.lang.Object
- - java.lang.Throwable
  - - java.lang.Exception
    - - org.apache.commons.cli.ParseException
      - - org.apache.commons.cli.UnrecognizedOptionException
        - - org.apache.commons.cli.AmbiguousOptionException

- All Implemented Interfaces:
  :   Serializable

  ---

    

  ```
  public class AmbiguousOptionException
  extends UnrecognizedOptionException
  ```

  Exception thrown when an option can't be identified from a partial name.

  Since:
  :   1.3

  Version:
  :   $Id: AmbiguousOptionException.java 1669814 2015-03-28 18:09:26Z britter $

  See Also:
  :   Serialized Form

- - ### Constructor Summary

    Constructors

    | Constructor and Description |
    | `AmbiguousOptionException(String option, Collection<String> matchingOptions)` Constructs a new AmbiguousOptionException. |
  - ### Method Summary

    All Methods Instance Methods Concrete Methods

    | Modifier and Type | Method and Description |
    | `Collection<String>` | `getMatchingOptions()` Returns the options matching the partial name. |

    - ### Methods inherited from class org.apache.commons.cli.UnrecognizedOptionException

      `getOption`
    - ### Methods inherited from class java.lang.Throwable

      `addSuppressed, fillInStackTrace, getCause, getLocalizedMessage, getMessage, getStackTrace, getSuppressed, initCause, printStackTrace, printStackTrace, printStackTrace, setStackTrace, toString`
    - ### Methods inherited from class java.lang.Object

      `clone, equals, finalize, getClass, hashCode, notify, notifyAll, wait, wait, wait`

- - ### Constructor Detail


    - #### AmbiguousOptionException

      ```
      public AmbiguousOptionException(String option,
                                      Collection<String> matchingOptions)
      ```

      Constructs a new AmbiguousOptionException.

      Parameters:
      :   `option` - the partial option name
      :   `matchingOptions` - the options matching the name
  - ### Method Detail


    - #### getMatchingOptions

      ```
      public Collection<String> getMatchingOptions()
      ```

      Returns the options matching the partial name.

      Returns:
      :   a collection of options matching the name


Skip navigation links


- Package
- Class
- Use
- Tree
- Deprecated
- Index
- Help

- Prev Class
- Next Class

- Frames
- No Frames

- All Classes

- Summary:
- Nested |
- Field |
- Constr |
- Method

- Detail:
- Field |
- Constr |
- Method

Copyright © 2002–2015 The Apache Software Foundation. All rights reserved.
