# Supplementary material for: ACBM: An Integrated Agent and Constraint Based Modeling Framework for Simulation of Microbial Communities
Source: Sci Rep. 2020 May 26;10:8695. doi: 10.1038/s41598-020-65659-w (PMC7250870; doi:10.1038/s41598-020-65659-w)
Supplement: Supplementary file 2 [file 41598_2020_65659_MOESM2_ESM.zip › ACBM1.4/lib/commons-cli-1.3/apidocs/org/apache/commons/cli/class-use/CommandLine.html]

Uses of Class org.apache.commons.cli.CommandLine (Apache Commons CLI 1.3 API)


JavaScript is disabled on your browser.


Skip navigation links


- Package
- Class
- Use
- Tree
- Deprecated
- Index
- Help

- Prev
- Next

- Frames
- No Frames

- All Classes

## Uses of Class org.apache.commons.cli.CommandLine

- - ### Uses of CommandLine in org.apache.commons.cli

    Fields in org.apache.commons.cli declared as CommandLine

    | Modifier and Type | Field and Description |
    |  |  |
    | --- | --- |
    | `protected CommandLine` | Parser.`cmd` Deprecated.  commandline instance |
    | `protected CommandLine` | DefaultParser.`cmd` The command-line instance. |

    Methods in org.apache.commons.cli that return CommandLine

    | Modifier and Type | Method and Description |
    |  |  |
    | --- | --- |
    | `CommandLine` | Parser.`parse(Options options, String[] arguments)` Deprecated.  Parses the specified `arguments` based on the specified `Options`. |
    | `CommandLine` | DefaultParser.`parse(Options options, String[] arguments)` |
    | `CommandLine` | CommandLineParser.`parse(Options options, String[] arguments)` Parse the arguments according to the specified options. |
    | `CommandLine` | Parser.`parse(Options options, String[] arguments, boolean stopAtNonOption)` Deprecated.  Parses the specified `arguments` based on the specified `Options`. |
    | `CommandLine` | DefaultParser.`parse(Options options, String[] arguments, boolean stopAtNonOption)` |
    | `CommandLine` | CommandLineParser.`parse(Options options, String[] arguments, boolean stopAtNonOption)` Parse the arguments according to the specified options. |
    | `CommandLine` | Parser.`parse(Options options, String[] arguments, Properties properties)` Deprecated.  Parse the arguments according to the specified options and properties. |
    | `CommandLine` | DefaultParser.`parse(Options options, String[] arguments, Properties properties)` Parse the arguments according to the specified options and properties. |
    | `CommandLine` | Parser.`parse(Options options, String[] arguments, Properties properties, boolean stopAtNonOption)` Deprecated.  Parse the arguments according to the specified options and properties. |
    | `CommandLine` | DefaultParser.`parse(Options options, String[] arguments, Properties properties, boolean stopAtNonOption)` Parse the arguments according to the specified options and properties. |

Skip navigation links


- Package
- Class
- Use
- Tree
- Deprecated
- Index
- Help

- Prev
- Next

- Frames
- No Frames

- All Classes

Copyright © 2002–2015 The Apache Software Foundation. All rights reserved.
