# Supplementary material for: ACBM: An Integrated Agent and Constraint Based Modeling Framework for Simulation of Microbial Communities
Source: Sci Rep. 2020 May 26;10:8695. doi: 10.1038/s41598-020-65659-w (PMC7250870; doi:10.1038/s41598-020-65659-w)
Supplement: Supplementary file 2 [file 41598_2020_65659_MOESM2_ESM.zip › ACBM1.4/lib/commons-cli-1.3/apidocs/org/apache/commons/cli/class-use/Option.Builder.html]

Uses of Class org.apache.commons.cli.Option.Builder (Apache Commons CLI 1.3 API)


JavaScript is disabled on your browser.


Skip navigation links


- Package
- Class
- Use
- Tree
- Deprecated
- Index
- Help

- Prev
- Next

- Frames
- No Frames

- All Classes

## Uses of Class org.apache.commons.cli.Option.Builder

- - ### Uses of Option.Builder in org.apache.commons.cli

    Methods in org.apache.commons.cli that return Option.Builder

    | Modifier and Type | Method and Description |
    |  |  |
    | --- | --- |
    | `Option.Builder` | Option.Builder.`argName(String argName)` Sets the display name for the argument value. |
    | `static Option.Builder` | Option.`builder()` Returns a `Option.Builder` to create an `Option` using descriptive methods. |
    | `static Option.Builder` | Option.`builder(String opt)` Returns a `Option.Builder` to create an `Option` using descriptive methods. |
    | `Option.Builder` | Option.Builder.`desc(String description)` Sets the description for this option. |
    | `Option.Builder` | Option.Builder.`hasArg()` Indicates that the Option will require an argument. |
    | `Option.Builder` | Option.Builder.`hasArg(boolean hasArg)` Indicates if the Option has an argument or not. |
    | `Option.Builder` | Option.Builder.`hasArgs()` Indicates that the Option can have unlimited argument values. |
    | `Option.Builder` | Option.Builder.`longOpt(String longOpt)` Sets the long name of the Option. |
    | `Option.Builder` | Option.Builder.`numberOfArgs(int numberOfArgs)` Sets the number of argument values the Option can take. |
    | `Option.Builder` | Option.Builder.`optionalArg(boolean isOptional)` Sets whether the Option can have an optional argument. |
    | `Option.Builder` | Option.Builder.`required()` Marks this Option as required. |
    | `Option.Builder` | Option.Builder.`required(boolean required)` Sets whether the Option is mandatory. |
    | `Option.Builder` | Option.Builder.`type(Class<?> type)` Sets the type of the Option. |
    | `Option.Builder` | Option.Builder.`valueSeparator()` The Option will use '=' as a means to separate argument value. |
    | `Option.Builder` | Option.Builder.`valueSeparator(char sep)` The Option will use `sep` as a means to separate argument values. |

Skip navigation links


- Package
- Class
- Use
- Tree
- Deprecated
- Index
- Help

- Prev
- Next

- Frames
- No Frames

- All Classes

Copyright © 2002–2015 The Apache Software Foundation. All rights reserved.
