# Supplementary material for: ACBM: An Integrated Agent and Constraint Based Modeling Framework for Simulation of Microbial Communities
Source: Sci Rep. 2020 May 26;10:8695. doi: 10.1038/s41598-020-65659-w (PMC7250870; doi:10.1038/s41598-020-65659-w)
Supplement: Supplementary file 2 [file 41598_2020_65659_MOESM2_ESM.zip › ACBM1.4/lib/commons-cli-1.3/apidocs/org/apache/commons/cli/class-use/Option.html]

Uses of Class org.apache.commons.cli.Option (Apache Commons CLI 1.3 API)


JavaScript is disabled on your browser.


Skip navigation links


- Package
- Class
- Use
- Tree
- Deprecated
- Index
- Help

- Prev
- Next

- Frames
- No Frames

- All Classes

## Uses of Class org.apache.commons.cli.Option

- - ### Uses of Option in org.apache.commons.cli

    Fields in org.apache.commons.cli declared as Option

    | Modifier and Type | Field and Description |
    |  |  |
    | --- | --- |
    | `protected Option` | DefaultParser.`currentOption` The last option parsed. |

    Fields in org.apache.commons.cli with type parameters of type Option

    | Modifier and Type | Field and Description |
    |  |  |
    | --- | --- |
    | `protected Comparator<Option>` | HelpFormatter.`optionComparator` Comparator used to sort the options when they output in help text Defaults to case-insensitive alphabetical sorting by option key |

    Methods in org.apache.commons.cli that return Option

    | Modifier and Type | Method and Description |
    |  |  |
    | --- | --- |
    | `Option` | Option.Builder.`build()` Constructs an Option with the values declared by this `Option.Builder`. |
    | `static Option` | OptionBuilder.`create()` Deprecated.  Create an Option using the current settings |
    | `static Option` | OptionBuilder.`create(char opt)` Deprecated.  Create an Option using the current settings and with the specified Option `char`. |
    | `static Option` | OptionBuilder.`create(String opt)` Deprecated.  Create an Option using the current settings and with the specified Option `char`. |
    | `Option` | MissingArgumentException.`getOption()` Return the option requiring an argument that wasn't provided on the command line. |
    | `Option` | AlreadySelectedException.`getOption()` Returns the option that was added to the group and triggered the exception. |
    | `Option` | Options.`getOption(String opt)` Retrieve the `Option` matching the long or short name specified. |
    | `Option[]` | CommandLine.`getOptions()` Returns an array of the processed `Option`s. |

    Methods in org.apache.commons.cli that return types with arguments of type Option

    | Modifier and Type | Method and Description |
    |  |  |
    | --- | --- |
    | `Comparator<Option>` | HelpFormatter.`getOptionComparator()` Comparator used to sort the options when they output in help text. |
    | `Collection<Option>` | Options.`getOptions()` Retrieve a read-only list of options in this set |
    | `Collection<Option>` | OptionGroup.`getOptions()` |
    | `Iterator<Option>` | CommandLine.`iterator()` Returns an iterator over the Option members of CommandLine. |

    Methods in org.apache.commons.cli with parameters of type Option

    | Modifier and Type | Method and Description |
    |  |  |
    | --- | --- |
    | `Options` | Options.`addOption(Option opt)` Adds an option instance |
    | `OptionGroup` | OptionGroup.`addOption(Option option)` Add the specified `Option` to this group. |
    | `protected void` | CommandLine.`addOption(Option opt)` Add an option to the command line. |
    | `OptionGroup` | Options.`getOptionGroup(Option opt)` Returns the OptionGroup the `opt` belongs to. |
    | `void` | Parser.`processArgs(Option opt, ListIterator<String> iter)` Deprecated.  Process the argument values for the specified Option `opt` using the values retrieved from the specified iterator `iter`. |
    | `void` | OptionGroup.`setSelected(Option option)` Set the selected option of this group to `name`. |

    Method parameters in org.apache.commons.cli with type arguments of type Option

    | Modifier and Type | Method and Description |
    |  |  |
    | --- | --- |
    | `void` | HelpFormatter.`setOptionComparator(Comparator<Option> comparator)` Set the comparator used to sort the options when they output in help text. |

    Constructors in org.apache.commons.cli with parameters of type Option

    | Constructor and Description |
    |  |
    | --- |
    | `AlreadySelectedException(OptionGroup group, Option option)` Construct a new `AlreadySelectedException` for the specified option group. |
    | `MissingArgumentException(Option option)` Construct a new `MissingArgumentException` with the specified detail message. |

Skip navigation links


- Package
- Class
- Use
- Tree
- Deprecated
- Index
- Help

- Prev
- Next

- Frames
- No Frames

- All Classes

Copyright © 2002–2015 The Apache Software Foundation. All rights reserved.
