# Supplementary material for: ACBM: An Integrated Agent and Constraint Based Modeling Framework for Simulation of Microbial Communities
Source: Sci Rep. 2020 May 26;10:8695. doi: 10.1038/s41598-020-65659-w (PMC7250870; doi:10.1038/s41598-020-65659-w)
Supplement: Supplementary file 2 [file 41598_2020_65659_MOESM2_ESM.zip › ACBM1.4/lib/commons-cli-1.3/apidocs/org/apache/commons/cli/class-use/OptionBuilder.html]

Uses of Class org.apache.commons.cli.OptionBuilder (Apache Commons CLI 1.3 API)


JavaScript is disabled on your browser.


Skip navigation links


- Package
- Class
- Use
- Tree
- Deprecated
- Index
- Help

- Prev
- Next

- Frames
- No Frames

- All Classes

## Uses of Class org.apache.commons.cli.OptionBuilder

- - ### Uses of OptionBuilder in org.apache.commons.cli

    Methods in org.apache.commons.cli that return OptionBuilder

    | Modifier and Type | Method and Description |
    |  |  |
    | --- | --- |
    | `static OptionBuilder` | OptionBuilder.`hasArg()` Deprecated.  The next Option created will require an argument value. |
    | `static OptionBuilder` | OptionBuilder.`hasArg(boolean hasArg)` Deprecated.  The next Option created will require an argument value if `hasArg` is true. |
    | `static OptionBuilder` | OptionBuilder.`hasArgs()` Deprecated.  The next Option created can have unlimited argument values. |
    | `static OptionBuilder` | OptionBuilder.`hasArgs(int num)` Deprecated.  The next Option created can have `num` argument values. |
    | `static OptionBuilder` | OptionBuilder.`hasOptionalArg()` Deprecated.  The next Option can have an optional argument. |
    | `static OptionBuilder` | OptionBuilder.`hasOptionalArgs()` Deprecated.  The next Option can have an unlimited number of optional arguments. |
    | `static OptionBuilder` | OptionBuilder.`hasOptionalArgs(int numArgs)` Deprecated.  The next Option can have the specified number of optional arguments. |
    | `static OptionBuilder` | OptionBuilder.`isRequired()` Deprecated.  The next Option created will be required. |
    | `static OptionBuilder` | OptionBuilder.`isRequired(boolean newRequired)` Deprecated.  The next Option created will be required if `required` is true. |
    | `static OptionBuilder` | OptionBuilder.`withArgName(String name)` Deprecated.  The next Option created will have the specified argument value name. |
    | `static OptionBuilder` | OptionBuilder.`withDescription(String newDescription)` Deprecated.  The next Option created will have the specified description |
    | `static OptionBuilder` | OptionBuilder.`withLongOpt(String newLongopt)` Deprecated.  The next Option created will have the following long option value. |
    | `static OptionBuilder` | OptionBuilder.`withType(Class<?> newType)` Deprecated.  The next Option created will have a value that will be an instance of `type`. |
    | `static OptionBuilder` | OptionBuilder.`withType(Object newType)` Deprecated. since 1.3, use `withType(Class)` instead |
    | `static OptionBuilder` | OptionBuilder.`withValueSeparator()` Deprecated.  The next Option created uses '`=`' as a means to separate argument values. |
    | `static OptionBuilder` | OptionBuilder.`withValueSeparator(char sep)` Deprecated.  The next Option created uses `sep` as a means to separate argument values. |

Skip navigation links


- Package
- Class
- Use
- Tree
- Deprecated
- Index
- Help

- Prev
- Next

- Frames
- No Frames

- All Classes

Copyright © 2002–2015 The Apache Software Foundation. All rights reserved.
