# Supplementary material for: ACBM: An Integrated Agent and Constraint Based Modeling Framework for Simulation of Microbial Communities
Source: Sci Rep. 2020 May 26;10:8695. doi: 10.1038/s41598-020-65659-w (PMC7250870; doi:10.1038/s41598-020-65659-w)
Supplement: Supplementary file 2 [file 41598_2020_65659_MOESM2_ESM.zip › ACBM1.4/lib/commons-cli-1.3/apidocs/org/apache/commons/cli/class-use/OptionGroup.html]

Uses of Class org.apache.commons.cli.OptionGroup (Apache Commons CLI 1.3 API)


JavaScript is disabled on your browser.


Skip navigation links


- Package
- Class
- Use
- Tree
- Deprecated
- Index
- Help

- Prev
- Next

- Frames
- No Frames

- All Classes

## Uses of Class org.apache.commons.cli.OptionGroup

- - ### Uses of OptionGroup in org.apache.commons.cli

    Methods in org.apache.commons.cli that return OptionGroup

    | Modifier and Type | Method and Description |
    |  |  |
    | --- | --- |
    | `OptionGroup` | OptionGroup.`addOption(Option option)` Add the specified `Option` to this group. |
    | `OptionGroup` | AlreadySelectedException.`getOptionGroup()` Returns the option group where another option has been selected. |
    | `OptionGroup` | Options.`getOptionGroup(Option opt)` Returns the OptionGroup the `opt` belongs to. |

    Methods in org.apache.commons.cli with parameters of type OptionGroup

    | Modifier and Type | Method and Description |
    |  |  |
    | --- | --- |
    | `Options` | Options.`addOptionGroup(OptionGroup group)` Add the specified option group. |

    Constructors in org.apache.commons.cli with parameters of type OptionGroup

    | Constructor and Description |
    |  |
    | --- |
    | `AlreadySelectedException(OptionGroup group, Option option)` Construct a new `AlreadySelectedException` for the specified option group. |

Skip navigation links


- Package
- Class
- Use
- Tree
- Deprecated
- Index
- Help

- Prev
- Next

- Frames
- No Frames

- All Classes

Copyright © 2002–2015 The Apache Software Foundation. All rights reserved.
