# Supplementary material for: ACBM: An Integrated Agent and Constraint Based Modeling Framework for Simulation of Microbial Communities
Source: Sci Rep. 2020 May 26;10:8695. doi: 10.1038/s41598-020-65659-w (PMC7250870; doi:10.1038/s41598-020-65659-w)
Supplement: Supplementary file 2 [file 41598_2020_65659_MOESM2_ESM.zip › ACBM1.4/lib/commons-cli-1.3/apidocs/org/apache/commons/cli/class-use/Options.html]

Uses of Class org.apache.commons.cli.Options (Apache Commons CLI 1.3 API)


JavaScript is disabled on your browser.


Skip navigation links


- Package
- Class
- Use
- Tree
- Deprecated
- Index
- Help

- Prev
- Next

- Frames
- No Frames

- All Classes

## Uses of Class org.apache.commons.cli.Options

- - ### Uses of Options in org.apache.commons.cli

    Fields in org.apache.commons.cli declared as Options

    | Modifier and Type | Field and Description |
    |  |  |
    | --- | --- |
    | `protected Options` | DefaultParser.`options` The current options. |

    Methods in org.apache.commons.cli that return Options

    | Modifier and Type | Method and Description |
    |  |  |
    | --- | --- |
    | `Options` | Options.`addOption(Option opt)` Adds an option instance |
    | `Options` | Options.`addOption(String opt, boolean hasArg, String description)` Add an option that only contains a short-name. |
    | `Options` | Options.`addOption(String opt, String description)` Add an option that only contains a short name. |
    | `Options` | Options.`addOption(String opt, String longOpt, boolean hasArg, String description)` Add an option that contains a short-name and a long-name. |
    | `Options` | Options.`addOptionGroup(OptionGroup group)` Add the specified option group. |
    | `protected Options` | Parser.`getOptions()` Deprecated. |
    | `static Options` | PatternOptionBuilder.`parsePattern(String pattern)` Returns the `Options` instance represented by `pattern`. |

    Methods in org.apache.commons.cli with parameters of type Options

    | Modifier and Type | Method and Description |
    |  |  |
    | --- | --- |
    | `protected String[]` | PosixParser.`flatten(Options options, String[] arguments, boolean stopAtNonOption)` Deprecated.  An implementation of `Parser`'s abstract `flatten` method. |
    | `protected abstract String[]` | Parser.`flatten(Options opts, String[] arguments, boolean stopAtNonOption)` Deprecated.  Subclasses must implement this method to reduce the `arguments` that have been passed to the parse method. |
    | `protected String[]` | GnuParser.`flatten(Options options, String[] arguments, boolean stopAtNonOption)` Deprecated.  This flatten method does so using the following rules: If an `Option` exists for the first character of the `arguments` entry **AND** an `Option` does not exist for the whole `argument` then add the first character as an option to the processed tokens list e.g. |
    | `protected String[]` | BasicParser.`flatten(Options options, String[] arguments, boolean stopAtNonOption)` Deprecated.  A simple implementation of `Parser`'s abstract `flatten` method. |
    | `CommandLine` | Parser.`parse(Options options, String[] arguments)` Deprecated.  Parses the specified `arguments` based on the specified `Options`. |
    | `CommandLine` | DefaultParser.`parse(Options options, String[] arguments)` |
    | `CommandLine` | CommandLineParser.`parse(Options options, String[] arguments)` Parse the arguments according to the specified options. |
    | `CommandLine` | Parser.`parse(Options options, String[] arguments, boolean stopAtNonOption)` Deprecated.  Parses the specified `arguments` based on the specified `Options`. |
    | `CommandLine` | DefaultParser.`parse(Options options, String[] arguments, boolean stopAtNonOption)` |
    | `CommandLine` | CommandLineParser.`parse(Options options, String[] arguments, boolean stopAtNonOption)` Parse the arguments according to the specified options. |
    | `CommandLine` | Parser.`parse(Options options, String[] arguments, Properties properties)` Deprecated.  Parse the arguments according to the specified options and properties. |
    | `CommandLine` | DefaultParser.`parse(Options options, String[] arguments, Properties properties)` Parse the arguments according to the specified options and properties. |
    | `CommandLine` | Parser.`parse(Options options, String[] arguments, Properties properties, boolean stopAtNonOption)` Deprecated.  Parse the arguments according to the specified options and properties. |
    | `CommandLine` | DefaultParser.`parse(Options options, String[] arguments, Properties properties, boolean stopAtNonOption)` Parse the arguments according to the specified options and properties. |
    | `void` | HelpFormatter.`printHelp(int width, String cmdLineSyntax, String header, Options options, String footer)` Print the help for `options` with the specified command line syntax. |
    | `void` | HelpFormatter.`printHelp(int width, String cmdLineSyntax, String header, Options options, String footer, boolean autoUsage)` Print the help for `options` with the specified command line syntax. |
    | `void` | HelpFormatter.`printHelp(PrintWriter pw, int width, String cmdLineSyntax, String header, Options options, int leftPad, int descPad, String footer)` Print the help for `options` with the specified command line syntax. |
    | `void` | HelpFormatter.`printHelp(PrintWriter pw, int width, String cmdLineSyntax, String header, Options options, int leftPad, int descPad, String footer, boolean autoUsage)` Print the help for `options` with the specified command line syntax. |
    | `void` | HelpFormatter.`printHelp(String cmdLineSyntax, Options options)` Print the help for `options` with the specified command line syntax. |
    | `void` | HelpFormatter.`printHelp(String cmdLineSyntax, Options options, boolean autoUsage)` Print the help for `options` with the specified command line syntax. |
    | `void` | HelpFormatter.`printHelp(String cmdLineSyntax, String header, Options options, String footer)` Print the help for `options` with the specified command line syntax. |
    | `void` | HelpFormatter.`printHelp(String cmdLineSyntax, String header, Options options, String footer, boolean autoUsage)` Print the help for `options` with the specified command line syntax. |
    | `void` | HelpFormatter.`printOptions(PrintWriter pw, int width, Options options, int leftPad, int descPad)` Print the help for the specified Options to the specified writer, using the specified width, left padding and description padding. |
    | `void` | HelpFormatter.`printUsage(PrintWriter pw, int width, String app, Options options)` Prints the usage statement for the specified application. |
    | `protected StringBuffer` | HelpFormatter.`renderOptions(StringBuffer sb, int width, Options options, int leftPad, int descPad)` Render the specified Options and return the rendered Options in a StringBuffer. |
    | `protected void` | Parser.`setOptions(Options options)` Deprecated. |

Skip navigation links


- Package
- Class
- Use
- Tree
- Deprecated
- Index
- Help

- Prev
- Next

- Frames
- No Frames

- All Classes

Copyright © 2002–2015 The Apache Software Foundation. All rights reserved.
