# Supplementary material for: ACBM: An Integrated Agent and Constraint Based Modeling Framework for Simulation of Microbial Communities
Source: Sci Rep. 2020 May 26;10:8695. doi: 10.1038/s41598-020-65659-w (PMC7250870; doi:10.1038/s41598-020-65659-w)
Supplement: Supplementary file 2 [file 41598_2020_65659_MOESM2_ESM.zip › ACBM1.4/lib/commons-cli-1.3/apidocs/org/apache/commons/cli/class-use/ParseException.html]

Uses of Class org.apache.commons.cli.ParseException (Apache Commons CLI 1.3 API)


JavaScript is disabled on your browser.


Skip navigation links


- Package
- Class
- Use
- Tree
- Deprecated
- Index
- Help

- Prev
- Next

- Frames
- No Frames

- All Classes

## Uses of Class org.apache.commons.cli.ParseException

- - ### Uses of ParseException in org.apache.commons.cli

    Subclasses of ParseException in org.apache.commons.cli

    | Modifier and Type | Class and Description |
    |  |  |
    | --- | --- |
    | `class` | `AlreadySelectedException` Thrown when more than one option in an option group has been provided. |
    | `class` | `AmbiguousOptionException` Exception thrown when an option can't be identified from a partial name. |
    | `class` | `MissingArgumentException` Thrown when an option requiring an argument is not provided with an argument. |
    | `class` | `MissingOptionException` Thrown when a required option has not been provided. |
    | `class` | `UnrecognizedOptionException` Exception thrown during parsing signalling an unrecognized option was seen. |

    Methods in org.apache.commons.cli that throw ParseException

    | Modifier and Type | Method and Description |
    |  |  |
    | --- | --- |
    | `static Class<?>` | TypeHandler.`createClass(String classname)` Returns the class whose name is `classname`. |
    | `static Number` | TypeHandler.`createNumber(String str)` Create a number from a String. |
    | `static Object` | TypeHandler.`createObject(String classname)` Create an Object from the classname and empty constructor. |
    | `static URL` | TypeHandler.`createURL(String str)` Returns the URL represented by `str`. |
    | `static Object` | TypeHandler.`createValue(String str, Class<?> clazz)` Returns the `Object` of type `clazz` with the value of `str`. |
    | `static Object` | TypeHandler.`createValue(String str, Object obj)` Returns the `Object` of type `obj` with the value of `str`. |
    | `protected String[]` | PosixParser.`flatten(Options options, String[] arguments, boolean stopAtNonOption)` Deprecated.  An implementation of `Parser`'s abstract `flatten` method. |
    | `protected abstract String[]` | Parser.`flatten(Options opts, String[] arguments, boolean stopAtNonOption)` Deprecated.  Subclasses must implement this method to reduce the `arguments` that have been passed to the parse method. |
    | `Object` | CommandLine.`getParsedOptionValue(String opt)` Return a version of this `Option` converted to a particular type. |
    | `protected void` | DefaultParser.`handleConcatenatedOptions(String token)` Breaks `token` into its constituent parts using the following algorithm. |
    | `CommandLine` | Parser.`parse(Options options, String[] arguments)` Deprecated.  Parses the specified `arguments` based on the specified `Options`. |
    | `CommandLine` | DefaultParser.`parse(Options options, String[] arguments)` |
    | `CommandLine` | CommandLineParser.`parse(Options options, String[] arguments)` Parse the arguments according to the specified options. |
    | `CommandLine` | Parser.`parse(Options options, String[] arguments, boolean stopAtNonOption)` Deprecated.  Parses the specified `arguments` based on the specified `Options`. |
    | `CommandLine` | DefaultParser.`parse(Options options, String[] arguments, boolean stopAtNonOption)` |
    | `CommandLine` | CommandLineParser.`parse(Options options, String[] arguments, boolean stopAtNonOption)` Parse the arguments according to the specified options. |
    | `CommandLine` | Parser.`parse(Options options, String[] arguments, Properties properties)` Deprecated.  Parse the arguments according to the specified options and properties. |
    | `CommandLine` | DefaultParser.`parse(Options options, String[] arguments, Properties properties)` Parse the arguments according to the specified options and properties. |
    | `CommandLine` | Parser.`parse(Options options, String[] arguments, Properties properties, boolean stopAtNonOption)` Deprecated.  Parse the arguments according to the specified options and properties. |
    | `CommandLine` | DefaultParser.`parse(Options options, String[] arguments, Properties properties, boolean stopAtNonOption)` Parse the arguments according to the specified options and properties. |
    | `void` | Parser.`processArgs(Option opt, ListIterator<String> iter)` Deprecated.  Process the argument values for the specified Option `opt` using the values retrieved from the specified iterator `iter`. |
    | `protected void` | Parser.`processOption(String arg, ListIterator<String> iter)` Deprecated.  Process the Option specified by `arg` using the values retrieved from the specified iterator `iter`. |
    | `protected void` | Parser.`processProperties(Properties properties)` Deprecated.  Sets the values of Options using the values in `properties`. |

Skip navigation links


- Package
- Class
- Use
- Tree
- Deprecated
- Index
- Help

- Prev
- Next

- Frames
- No Frames

- All Classes

Copyright © 2002–2015 The Apache Software Foundation. All rights reserved.
