# Supplementary material for: ACBM: An Integrated Agent and Constraint Based Modeling Framework for Simulation of Microbial Communities
Source: Sci Rep. 2020 May 26;10:8695. doi: 10.1038/s41598-020-65659-w (PMC7250870; doi:10.1038/s41598-020-65659-w)
Supplement: Supplementary file 2 [file 41598_2020_65659_MOESM2_ESM.zip › ACBM1.4/lib/commons-cli-1.3/apidocs/org/apache/commons/cli/class-use/UnrecognizedOptionException.html]

Uses of Class org.apache.commons.cli.UnrecognizedOptionException (Apache Commons CLI 1.3 API)


JavaScript is disabled on your browser.


Skip navigation links


- Package
- Class
- Use
- Tree
- Deprecated
- Index
- Help

- Prev
- Next

- Frames
- No Frames

- All Classes

## Uses of Class org.apache.commons.cli.UnrecognizedOptionException

- - ### Uses of UnrecognizedOptionException in org.apache.commons.cli

    Subclasses of UnrecognizedOptionException in org.apache.commons.cli

    | Modifier and Type | Class and Description |
    |  |  |
    | --- | --- |
    | `class` | `AmbiguousOptionException` Exception thrown when an option can't be identified from a partial name. |

Skip navigation links


- Package
- Class
- Use
- Tree
- Deprecated
- Index
- Help

- Prev
- Next

- Frames
- No Frames

- All Classes

Copyright © 2002–2015 The Apache Software Foundation. All rights reserved.
