# Supplementary material for: ACBM: An Integrated Agent and Constraint Based Modeling Framework for Simulation of Microbial Communities
Source: Sci Rep. 2020 May 26;10:8695. doi: 10.1038/s41598-020-65659-w (PMC7250870; doi:10.1038/s41598-020-65659-w)
Supplement: Supplementary file 2 [file 41598_2020_65659_MOESM2_ESM.zip › ACBM1.4/lib/commons-cli-1.3/apidocs/org/apache/commons/cli/CommandLine.html]

CommandLine (Apache Commons CLI 1.3 API)


JavaScript is disabled on your browser.


Skip navigation links


- Package
- Class
- Use
- Tree
- Deprecated
- Index
- Help

- Prev Class
- Next Class

- Frames
- No Frames

- All Classes

- Summary:
- Nested |
- Field |
- Constr |
- Method

- Detail:
- Field |
- Constr |
- Method


org.apache.commons.cli

## Class CommandLine

- java.lang.Object
- - org.apache.commons.cli.CommandLine

- All Implemented Interfaces:
  :   Serializable

  ---

    

  ```
  public class CommandLine
  extends Object
  implements Serializable
  ```

  Represents list of arguments parsed against a `Options` descriptor.

  It allows querying of a boolean `hasOption(String opt)`,
  in addition to retrieving the `getOptionValue(String opt)`
  for options requiring arguments.

  Additionally, any left-over or unrecognized arguments,
  are available for further processing.

  Version:
  :   $Id: CommandLine.java 1444365 2013-02-09 14:21:27Z tn $

  See Also:
  :   Serialized Form

- - ### Constructor Summary

    Constructors

    | Modifier | Constructor and Description |
    | `protected` | `CommandLine()` Creates a command line. |
  - ### Method Summary

    All Methods Instance Methods Concrete Methods Deprecated Methods

    | Modifier and Type | Method and Description |
    | `protected void` | `addArg(String arg)` Add left-over unrecognized option/argument. |
    | `protected void` | `addOption(Option opt)` Add an option to the command line. |
    | `List<String>` | `getArgList()` Retrieve any left-over non-recognized options and arguments |
    | `String[]` | `getArgs()` Retrieve any left-over non-recognized options and arguments |
    | `Object` | `getOptionObject(char opt)` Return the `Object` type of this `Option`. |
    | `Object` | `getOptionObject(String opt)` Deprecated. due to System.err message. Instead use getParsedOptionValue(String) |
    | `Properties` | `getOptionProperties(String opt)` Retrieve the map of values associated to the option. |
    | `Option[]` | `getOptions()` Returns an array of the processed `Option`s. |
    | `String` | `getOptionValue(char opt)` Retrieve the first argument, if any, of this option. |
    | `String` | `getOptionValue(char opt, String defaultValue)` Retrieve the argument, if any, of an option. |
    | `String` | `getOptionValue(String opt)` Retrieve the first argument, if any, of this option. |
    | `String` | `getOptionValue(String opt, String defaultValue)` Retrieve the first argument, if any, of an option. |
    | `String[]` | `getOptionValues(char opt)` Retrieves the array of values, if any, of an option. |
    | `String[]` | `getOptionValues(String opt)` Retrieves the array of values, if any, of an option. |
    | `Object` | `getParsedOptionValue(String opt)` Return a version of this `Option` converted to a particular type. |
    | `boolean` | `hasOption(char opt)` Query to see if an option has been set. |
    | `boolean` | `hasOption(String opt)` Query to see if an option has been set. |
    | `Iterator<Option>` | `iterator()` Returns an iterator over the Option members of CommandLine. |

    - ### Methods inherited from class java.lang.Object

      `clone, equals, finalize, getClass, hashCode, notify, notifyAll, toString, wait, wait, wait`

- - ### Constructor Detail


    - #### CommandLine

      ```
      protected CommandLine()
      ```

      Creates a command line.
  - ### Method Detail


    - #### hasOption

      ```
      public boolean hasOption(String opt)
      ```

      Query to see if an option has been set.

      Parameters:
      :   `opt` - Short name of the option

      Returns:
      :   true if set, false if not


    - #### hasOption

      ```
      public boolean hasOption(char opt)
      ```

      Query to see if an option has been set.

      Parameters:
      :   `opt` - character name of the option

      Returns:
      :   true if set, false if not


    - #### getOptionObject

      ```
      @Deprecated
      public Object getOptionObject(String opt)
      ```

      Deprecated. due to System.err message. Instead use getParsedOptionValue(String)

      Return the `Object` type of this `Option`.

      Parameters:
      :   `opt` - the name of the option

      Returns:
      :   the type of this `Option`


    - #### getParsedOptionValue

      ```
      public Object getParsedOptionValue(String opt)
                                  throws ParseException
      ```

      Return a version of this `Option` converted to a particular type.

      Parameters:
      :   `opt` - the name of the option

      Returns:
      :   the value parsed into a particular object

      Throws:
      :   `ParseException` - if there are problems turning the option value into the desired type

      Since:
      :   1.2

      See Also:
      :   `PatternOptionBuilder`


    - #### getOptionObject

      ```
      public Object getOptionObject(char opt)
      ```

      Return the `Object` type of this `Option`.

      Parameters:
      :   `opt` - the name of the option

      Returns:
      :   the type of opt


    - #### getOptionValue

      ```
      public String getOptionValue(String opt)
      ```

      Retrieve the first argument, if any, of this option.

      Parameters:
      :   `opt` - the name of the option

      Returns:
      :   Value of the argument if option is set, and has an argument,
          otherwise null.


    - #### getOptionValue

      ```
      public String getOptionValue(char opt)
      ```

      Retrieve the first argument, if any, of this option.

      Parameters:
      :   `opt` - the character name of the option

      Returns:
      :   Value of the argument if option is set, and has an argument,
          otherwise null.


    - #### getOptionValues

      ```
      public String[] getOptionValues(String opt)
      ```

      Retrieves the array of values, if any, of an option.

      Parameters:
      :   `opt` - string name of the option

      Returns:
      :   Values of the argument if option is set, and has an argument,
          otherwise null.


    - #### getOptionValues

      ```
      public String[] getOptionValues(char opt)
      ```

      Retrieves the array of values, if any, of an option.

      Parameters:
      :   `opt` - character name of the option

      Returns:
      :   Values of the argument if option is set, and has an argument,
          otherwise null.


    - #### getOptionValue

      ```
      public String getOptionValue(String opt,
                                   String defaultValue)
      ```

      Retrieve the first argument, if any, of an option.

      Parameters:
      :   `opt` - name of the option
      :   `defaultValue` - is the default value to be returned if the option
          is not specified

      Returns:
      :   Value of the argument if option is set, and has an argument,
          otherwise `defaultValue`.


    - #### getOptionValue

      ```
      public String getOptionValue(char opt,
                                   String defaultValue)
      ```

      Retrieve the argument, if any, of an option.

      Parameters:
      :   `opt` - character name of the option
      :   `defaultValue` - is the default value to be returned if the option
          is not specified

      Returns:
      :   Value of the argument if option is set, and has an argument,
          otherwise `defaultValue`.


    - #### getOptionProperties

      ```
      public Properties getOptionProperties(String opt)
      ```

      Retrieve the map of values associated to the option. This is convenient
      for options specifying Java properties like -Dparam1=value1
      -Dparam2=value2. The first argument of the option is the key, and
      the 2nd argument is the value. If the option has only one argument
      (-Dfoo) it is considered as a boolean flag and the value is
      "true".

      Parameters:
      :   `opt` - name of the option

      Returns:
      :   The Properties mapped by the option, never null
          even if the option doesn't exists

      Since:
      :   1.2


    - #### getArgs

      ```
      public String[] getArgs()
      ```

      Retrieve any left-over non-recognized options and arguments

      Returns:
      :   remaining items passed in but not parsed as an array


    - #### getArgList

      ```
      public List<String> getArgList()
      ```

      Retrieve any left-over non-recognized options and arguments

      Returns:
      :   remaining items passed in but not parsed as a `List`.


    - #### addArg

      ```
      protected void addArg(String arg)
      ```

      Add left-over unrecognized option/argument.

      Parameters:
      :   `arg` - the unrecognised option/argument.


    - #### addOption

      ```
      protected void addOption(Option opt)
      ```

      Add an option to the command line. The values of the option are stored.

      Parameters:
      :   `opt` - the processed option


    - #### iterator

      ```
      public Iterator<Option> iterator()
      ```

      Returns an iterator over the Option members of CommandLine.

      Returns:
      :   an `Iterator` over the processed `Option`
          members of this `CommandLine`


    - #### getOptions

      ```
      public Option[] getOptions()
      ```

      Returns an array of the processed `Option`s.

      Returns:
      :   an array of the processed `Option`s.


Skip navigation links


- Package
- Class
- Use
- Tree
- Deprecated
- Index
- Help

- Prev Class
- Next Class

- Frames
- No Frames

- All Classes

- Summary:
- Nested |
- Field |
- Constr |
- Method

- Detail:
- Field |
- Constr |
- Method

Copyright © 2002–2015 The Apache Software Foundation. All rights reserved.
