# Supplementary material for: ACBM: An Integrated Agent and Constraint Based Modeling Framework for Simulation of Microbial Communities
Source: Sci Rep. 2020 May 26;10:8695. doi: 10.1038/s41598-020-65659-w (PMC7250870; doi:10.1038/s41598-020-65659-w)
Supplement: Supplementary file 2 [file 41598_2020_65659_MOESM2_ESM.zip › ACBM1.4/lib/commons-cli-1.3/apidocs/org/apache/commons/cli/CommandLineParser.html]

CommandLineParser (Apache Commons CLI 1.3 API)


JavaScript is disabled on your browser.


Skip navigation links


- Package
- Class
- Use
- Tree
- Deprecated
- Index
- Help

- Prev Class
- Next Class

- Frames
- No Frames

- All Classes

- Summary:
- Nested |
- Field |
- Constr |
- Method

- Detail:
- Field |
- Constr |
- Method


org.apache.commons.cli

## Interface CommandLineParser

- All Known Implementing Classes:
  :   BasicParser, DefaultParser, GnuParser, Parser, PosixParser

  ---

    

  ```
  public interface CommandLineParser
  ```

  A class that implements the `CommandLineParser` interface
  can parse a String array according to the `Options` specified
  and return a `CommandLine`.

  Version:
  :   $Id: CommandLineParser.java 1443102 2013-02-06 18:12:16Z tn $

- - ### Method Summary

    All Methods Instance Methods Abstract Methods

    | Modifier and Type | Method and Description |
    | `CommandLine` | `parse(Options options, String[] arguments)` Parse the arguments according to the specified options. |
    | `CommandLine` | `parse(Options options, String[] arguments, boolean stopAtNonOption)` Parse the arguments according to the specified options. |

- - ### Method Detail


    - #### parse

      ```
      CommandLine parse(Options options,
                        String[] arguments)
                 throws ParseException
      ```

      Parse the arguments according to the specified options.

      Parameters:
      :   `options` - the specified Options
      :   `arguments` - the command line arguments

      Returns:
      :   the list of atomic option and value tokens

      Throws:
      :   `ParseException` - if there are any problems encountered
          while parsing the command line tokens.


    - #### parse

      ```
      CommandLine parse(Options options,
                        String[] arguments,
                        boolean stopAtNonOption)
                 throws ParseException
      ```

      Parse the arguments according to the specified options.

      Parameters:
      :   `options` - the specified Options
      :   `arguments` - the command line arguments
      :   `stopAtNonOption` - if true an unrecognized argument stops
          the parsing and the remaining arguments are added to the
          `CommandLine`s args list. If false an unrecognized
          argument triggers a ParseException.

      Returns:
      :   the list of atomic option and value tokens

      Throws:
      :   `ParseException` - if there are any problems encountered
          while parsing the command line tokens.


Skip navigation links


- Package
- Class
- Use
- Tree
- Deprecated
- Index
- Help

- Prev Class
- Next Class

- Frames
- No Frames

- All Classes

- Summary:
- Nested |
- Field |
- Constr |
- Method

- Detail:
- Field |
- Constr |
- Method

Copyright © 2002–2015 The Apache Software Foundation. All rights reserved.
