# Supplementary material for: ACBM: An Integrated Agent and Constraint Based Modeling Framework for Simulation of Microbial Communities
Source: Sci Rep. 2020 May 26;10:8695. doi: 10.1038/s41598-020-65659-w (PMC7250870; doi:10.1038/s41598-020-65659-w)
Supplement: Supplementary file 2 [file 41598_2020_65659_MOESM2_ESM.zip › ACBM1.4/lib/commons-cli-1.3/apidocs/org/apache/commons/cli/DefaultParser.html]

DefaultParser (Apache Commons CLI 1.3 API)


JavaScript is disabled on your browser.


Skip navigation links


- Package
- Class
- Use
- Tree
- Deprecated
- Index
- Help

- Prev Class
- Next Class

- Frames
- No Frames

- All Classes

- Summary:
- Nested |
- Field |
- Constr |
- Method

- Detail:
- Field |
- Constr |
- Method


org.apache.commons.cli

## Class DefaultParser

- java.lang.Object
- - org.apache.commons.cli.DefaultParser

- All Implemented Interfaces:
  :   CommandLineParser

  ---

    

  ```
  public class DefaultParser
  extends Object
  implements CommandLineParser
  ```

  Default parser.

  Since:
  :   1.3

  Version:
  :   $Id: DefaultParser.java 1677406 2015-05-03 14:27:31Z britter $

- - ### Field Summary

    Fields

    | Modifier and Type | Field and Description |
    | `protected CommandLine` | `cmd` The command-line instance. |
    | `protected Option` | `currentOption` The last option parsed. |
    | `protected String` | `currentToken` The token currently processed. |
    | `protected List` | `expectedOpts` The required options and groups expected to be found when parsing the command line. |
    | `protected Options` | `options` The current options. |
    | `protected boolean` | `skipParsing` Flag indicating if tokens should no longer be analysed and simply added as arguments of the command line. |
    | `protected boolean` | `stopAtNonOption` Flag indicating how unrecognized tokens are handled. |
  - ### Constructor Summary

    Constructors

    | Constructor and Description |
    | `DefaultParser()` |
  - ### Method Summary

    All Methods Instance Methods Concrete Methods

    | Modifier and Type | Method and Description |
    | `protected void` | `handleConcatenatedOptions(String token)` Breaks `token` into its constituent parts using the following algorithm. |
    | `CommandLine` | `parse(Options options, String[] arguments)` Parse the arguments according to the specified options. |
    | `CommandLine` | `parse(Options options, String[] arguments, boolean stopAtNonOption)` Parse the arguments according to the specified options. |
    | `CommandLine` | `parse(Options options, String[] arguments, Properties properties)` Parse the arguments according to the specified options and properties. |
    | `CommandLine` | `parse(Options options, String[] arguments, Properties properties, boolean stopAtNonOption)` Parse the arguments according to the specified options and properties. |

    - ### Methods inherited from class java.lang.Object

      `clone, equals, finalize, getClass, hashCode, notify, notifyAll, toString, wait, wait, wait`

- - ### Field Detail


    - #### cmd

      ```
      protected CommandLine cmd
      ```

      The command-line instance.


    - #### options

      ```
      protected Options options
      ```

      The current options.


    - #### stopAtNonOption

      ```
      protected boolean stopAtNonOption
      ```

      Flag indicating how unrecognized tokens are handled. true to stop
      the parsing and add the remaining tokens to the args list.
      false to throw an exception.


    - #### currentToken

      ```
      protected String currentToken
      ```

      The token currently processed.


    - #### currentOption

      ```
      protected Option currentOption
      ```

      The last option parsed.


    - #### skipParsing

      ```
      protected boolean skipParsing
      ```

      Flag indicating if tokens should no longer be analysed and simply added as arguments of the command line.


    - #### expectedOpts

      ```
      protected List expectedOpts
      ```

      The required options and groups expected to be found when parsing the command line.
  - ### Constructor Detail


    - #### DefaultParser

      ```
      public DefaultParser()
      ```
  - ### Method Detail


    - #### parse

      ```
      public CommandLine parse(Options options,
                               String[] arguments)
                        throws ParseException
      ```

      Description copied from interface: `CommandLineParser`

      Parse the arguments according to the specified options.

      Specified by:
      :   `parse` in interface `CommandLineParser`

      Parameters:
      :   `options` - the specified Options
      :   `arguments` - the command line arguments

      Returns:
      :   the list of atomic option and value tokens

      Throws:
      :   `ParseException` - if there are any problems encountered
          while parsing the command line tokens.


    - #### parse

      ```
      public CommandLine parse(Options options,
                               String[] arguments,
                               Properties properties)
                        throws ParseException
      ```

      Parse the arguments according to the specified options and properties.

      Parameters:
      :   `options` - the specified Options
      :   `arguments` - the command line arguments
      :   `properties` - command line option name-value pairs

      Returns:
      :   the list of atomic option and value tokens

      Throws:
      :   `ParseException` - if there are any problems encountered
          while parsing the command line tokens.


    - #### parse

      ```
      public CommandLine parse(Options options,
                               String[] arguments,
                               boolean stopAtNonOption)
                        throws ParseException
      ```

      Description copied from interface: `CommandLineParser`

      Parse the arguments according to the specified options.

      Specified by:
      :   `parse` in interface `CommandLineParser`

      Parameters:
      :   `options` - the specified Options
      :   `arguments` - the command line arguments
      :   `stopAtNonOption` - if true an unrecognized argument stops
          the parsing and the remaining arguments are added to the
          `CommandLine`s args list. If false an unrecognized
          argument triggers a ParseException.

      Returns:
      :   the list of atomic option and value tokens

      Throws:
      :   `ParseException` - if there are any problems encountered
          while parsing the command line tokens.


    - #### parse

      ```
      public CommandLine parse(Options options,
                               String[] arguments,
                               Properties properties,
                               boolean stopAtNonOption)
                        throws ParseException
      ```

      Parse the arguments according to the specified options and properties.

      Parameters:
      :   `options` - the specified Options
      :   `arguments` - the command line arguments
      :   `properties` - command line option name-value pairs
      :   `stopAtNonOption` - if true an unrecognized argument stops
          the parsing and the remaining arguments are added to the
          `CommandLine`s args list. If false an unrecognized
          argument triggers a ParseException.

      Returns:
      :   the list of atomic option and value tokens

      Throws:
      :   `ParseException` - if there are any problems encountered
          while parsing the command line tokens.


    - #### handleConcatenatedOptions

      ```
      protected void handleConcatenatedOptions(String token)
                                        throws ParseException
      ```

      Breaks `token` into its constituent parts
      using the following algorithm.
      - ignore the first character ("**-**")
      - foreach remaining character check if an `Option`
        exists with that id.
      - if an `Option` does exist then add that character
        prepended with "**-**" to the list of processed tokens.
      - if the `Option` can have an argument value and there
        are remaining characters in the token then add the remaining
        characters as a token to the list of processed tokens.
      - if an `Option` does **NOT** exist **AND**
        `stopAtNonOption` **IS** set then add the special token
        "**--**" followed by the remaining characters and also
        the remaining tokens directly to the processed tokens list.
      - if an `Option` does **NOT** exist **AND**
        `stopAtNonOption` **IS NOT** set then add that
        character prepended with "**-**".

      Parameters:
      :   `token` - The current token to be **burst**
          at the first non-Option encountered.

      Throws:
      :   `ParseException` - if there are any problems encountered
          while parsing the command line token.


Skip navigation links


- Package
- Class
- Use
- Tree
- Deprecated
- Index
- Help

- Prev Class
- Next Class

- Frames
- No Frames

- All Classes

- Summary:
- Nested |
- Field |
- Constr |
- Method

- Detail:
- Field |
- Constr |
- Method

Copyright © 2002–2015 The Apache Software Foundation. All rights reserved.
