# Supplementary material for: ACBM: An Integrated Agent and Constraint Based Modeling Framework for Simulation of Microbial Communities
Source: Sci Rep. 2020 May 26;10:8695. doi: 10.1038/s41598-020-65659-w (PMC7250870; doi:10.1038/s41598-020-65659-w)
Supplement: Supplementary file 2 [file 41598_2020_65659_MOESM2_ESM.zip › ACBM1.4/lib/commons-cli-1.3/apidocs/org/apache/commons/cli/GnuParser.html]

GnuParser (Apache Commons CLI 1.3 API)


JavaScript is disabled on your browser.


Skip navigation links


- Package
- Class
- Use
- Tree
- Deprecated
- Index
- Help

- Prev Class
- Next Class

- Frames
- No Frames

- All Classes

- Summary:
- Nested |
- Field |
- Constr |
- Method

- Detail:
- Field |
- Constr |
- Method


org.apache.commons.cli

## Class GnuParser

- java.lang.Object
- - org.apache.commons.cli.Parser
  - - org.apache.commons.cli.GnuParser

- All Implemented Interfaces:
  :   CommandLineParser

  ---

  Deprecated.

  since 1.3, use the `DefaultParser` instead

    

  ```
  @Deprecated
  public class GnuParser
  extends Parser
  ```

  The class GnuParser provides an implementation of the
  `flatten` method.

  Version:
  :   $Id: GnuParser.java 1445352 2013-02-12 20:48:19Z tn $

- - ### Field Summary

    - ### Fields inherited from class org.apache.commons.cli.Parser

      `cmd`
  - ### Constructor Summary

    Constructors

    | Constructor and Description |
    | `GnuParser()` Deprecated. |
  - ### Method Summary

    All Methods Instance Methods Concrete Methods Deprecated Methods

    | Modifier and Type | Method and Description |
    | `protected String[]` | `flatten(Options options, String[] arguments, boolean stopAtNonOption)` Deprecated.  This flatten method does so using the following rules: If an `Option` exists for the first character of the `arguments` entry **AND** an `Option` does not exist for the whole `argument` then add the first character as an option to the processed tokens list e.g. |

    - ### Methods inherited from class org.apache.commons.cli.Parser

      `checkRequiredOptions, getOptions, getRequiredOptions, parse, parse, parse, parse, processArgs, processOption, processProperties, setOptions`
    - ### Methods inherited from class java.lang.Object

      `clone, equals, finalize, getClass, hashCode, notify, notifyAll, toString, wait, wait, wait`

- - ### Constructor Detail


    - #### GnuParser

      ```
      public GnuParser()
      ```

      Deprecated.
  - ### Method Detail


    - #### flatten

      ```
      protected String[] flatten(Options options,
                                 String[] arguments,
                                 boolean stopAtNonOption)
      ```

      Deprecated.

      This flatten method does so using the following rules:
      1. If an `Option` exists for the first character of
         the `arguments` entry **AND** an `Option`
         does not exist for the whole `argument` then
         add the first character as an option to the processed tokens
         list e.g. "-D" and add the rest of the entry to the also.
      2. Otherwise just add the token to the processed tokens list.

      Specified by:
      :   `flatten` in class `Parser`

      Parameters:
      :   `options` - The Options to parse the arguments by.
      :   `arguments` - The arguments that have to be flattened.
      :   `stopAtNonOption` - specifies whether to stop flattening when
          a non option has been encountered

      Returns:
      :   a String array of the flattened arguments


Skip navigation links


- Package
- Class
- Use
- Tree
- Deprecated
- Index
- Help

- Prev Class
- Next Class

- Frames
- No Frames

- All Classes

- Summary:
- Nested |
- Field |
- Constr |
- Method

- Detail:
- Field |
- Constr |
- Method

Copyright © 2002–2015 The Apache Software Foundation. All rights reserved.
