# Supplementary material for: ACBM: An Integrated Agent and Constraint Based Modeling Framework for Simulation of Microbial Communities
Source: Sci Rep. 2020 May 26;10:8695. doi: 10.1038/s41598-020-65659-w (PMC7250870; doi:10.1038/s41598-020-65659-w)
Supplement: Supplementary file 2 [file 41598_2020_65659_MOESM2_ESM.zip › ACBM1.4/lib/commons-cli-1.3/apidocs/org/apache/commons/cli/HelpFormatter.html]

HelpFormatter (Apache Commons CLI 1.3 API)


JavaScript is disabled on your browser.


Skip navigation links


- Package
- Class
- Use
- Tree
- Deprecated
- Index
- Help

- Prev Class
- Next Class

- Frames
- No Frames

- All Classes

- Summary:
- Nested |
- Field |
- Constr |
- Method

- Detail:
- Field |
- Constr |
- Method


org.apache.commons.cli

## Class HelpFormatter

- java.lang.Object
- - org.apache.commons.cli.HelpFormatter

- ---

    

  ```
  public class HelpFormatter
  extends Object
  ```

  A formatter of help messages for command line options.

  Example:

  ```
   Options options = new Options();
   options.addOption(OptionBuilder.withLongOpt("file")
                                  .withDescription("The file to be processed")
                                  .hasArg()
                                  .withArgName("FILE")
                                  .isRequired()
                                  .create('f'));
   options.addOption(OptionBuilder.withLongOpt("version")
                                  .withDescription("Print the version of the application")
                                  .create('v'));
   options.addOption(OptionBuilder.withLongOpt("help").create('h'));
   
   String header = "Do something useful with an input file\n\n";
   String footer = "\nPlease report issues at http://example.com/issues";
   
   HelpFormatter formatter = new HelpFormatter();
   formatter.printHelp("myapp", header, options, footer, true);
  ```

  This produces the following output:

  ```
   usage: myapp -f <FILE> [-h] [-v]
   Do something useful with an input file
   
    -f,--file <FILE>   The file to be processed
    -h,--help
    -v,--version       Print the version of the application
   
   Please report issues at http://example.com/issues
  ```

  Version:
  :   $Id: HelpFormatter.java 1677407 2015-05-03 14:31:12Z britter $

- - ### Field Summary

    Fields

    | Modifier and Type | Field and Description |
    | `static String` | `DEFAULT_ARG_NAME` default name for an argument |
    | `static int` | `DEFAULT_DESC_PAD` number of space characters to be prefixed to each description line |
    | `static int` | `DEFAULT_LEFT_PAD` default padding to the left of each line |
    | `static String` | `DEFAULT_LONG_OPT_PREFIX` default prefix for long Option |
    | `static String` | `DEFAULT_LONG_OPT_SEPARATOR` default separator displayed between a long Option and its value |
    | `static String` | `DEFAULT_OPT_PREFIX` default prefix for shortOpts |
    | `static String` | `DEFAULT_SYNTAX_PREFIX` the string to display at the beginning of the usage statement |
    | `static int` | `DEFAULT_WIDTH` default number of characters per line |
    | `String` | `defaultArgName` Deprecated. Scope will be made private for next major version - use get/setArgName methods instead. |
    | `int` | `defaultDescPad` Deprecated. Scope will be made private for next major version - use get/setDescPadding methods instead. |
    | `int` | `defaultLeftPad` Deprecated. Scope will be made private for next major version - use get/setLeftPadding methods instead. |
    | `String` | `defaultLongOptPrefix` Deprecated. Scope will be made private for next major version - use get/setLongOptPrefix methods instead. |
    | `String` | `defaultNewLine` Deprecated. Scope will be made private for next major version - use get/setNewLine methods instead. |
    | `String` | `defaultOptPrefix` Deprecated. Scope will be made private for next major version - use get/setOptPrefix methods instead. |
    | `String` | `defaultSyntaxPrefix` Deprecated. Scope will be made private for next major version - use get/setSyntaxPrefix methods instead. |
    | `int` | `defaultWidth` Deprecated. Scope will be made private for next major version - use get/setWidth methods instead. |
    | `protected Comparator<Option>` | `optionComparator` Comparator used to sort the options when they output in help text Defaults to case-insensitive alphabetical sorting by option key |
  - ### Constructor Summary

    Constructors

    | Constructor and Description |
    | `HelpFormatter()` |
  - ### Method Summary

    All Methods Instance Methods Concrete Methods

    | Modifier and Type | Method and Description |
    | `protected String` | `createPadding(int len)` Return a String of padding of length `len`. |
    | `protected int` | `findWrapPos(String text, int width, int startPos)` Finds the next text wrap position after `startPos` for the text in `text` with the column width `width`. |
    | `String` | `getArgName()` Returns the 'argName'. |
    | `int` | `getDescPadding()` Returns the 'descPadding'. |
    | `int` | `getLeftPadding()` Returns the 'leftPadding'. |
    | `String` | `getLongOptPrefix()` Returns the 'longOptPrefix'. |
    | `String` | `getLongOptSeparator()` Returns the separator displayed between a long option and its value. |
    | `String` | `getNewLine()` Returns the 'newLine'. |
    | `Comparator<Option>` | `getOptionComparator()` Comparator used to sort the options when they output in help text. |
    | `String` | `getOptPrefix()` Returns the 'optPrefix'. |
    | `String` | `getSyntaxPrefix()` Returns the 'syntaxPrefix'. |
    | `int` | `getWidth()` Returns the 'width'. |
    | `void` | `printHelp(int width, String cmdLineSyntax, String header, Options options, String footer)` Print the help for `options` with the specified command line syntax. |
    | `void` | `printHelp(int width, String cmdLineSyntax, String header, Options options, String footer, boolean autoUsage)` Print the help for `options` with the specified command line syntax. |
    | `void` | `printHelp(PrintWriter pw, int width, String cmdLineSyntax, String header, Options options, int leftPad, int descPad, String footer)` Print the help for `options` with the specified command line syntax. |
    | `void` | `printHelp(PrintWriter pw, int width, String cmdLineSyntax, String header, Options options, int leftPad, int descPad, String footer, boolean autoUsage)` Print the help for `options` with the specified command line syntax. |
    | `void` | `printHelp(String cmdLineSyntax, Options options)` Print the help for `options` with the specified command line syntax. |
    | `void` | `printHelp(String cmdLineSyntax, Options options, boolean autoUsage)` Print the help for `options` with the specified command line syntax. |
    | `void` | `printHelp(String cmdLineSyntax, String header, Options options, String footer)` Print the help for `options` with the specified command line syntax. |
    | `void` | `printHelp(String cmdLineSyntax, String header, Options options, String footer, boolean autoUsage)` Print the help for `options` with the specified command line syntax. |
    | `void` | `printOptions(PrintWriter pw, int width, Options options, int leftPad, int descPad)` Print the help for the specified Options to the specified writer, using the specified width, left padding and description padding. |
    | `void` | `printUsage(PrintWriter pw, int width, String cmdLineSyntax)` Print the cmdLineSyntax to the specified writer, using the specified width. |
    | `void` | `printUsage(PrintWriter pw, int width, String app, Options options)` Prints the usage statement for the specified application. |
    | `void` | `printWrapped(PrintWriter pw, int width, int nextLineTabStop, String text)` Print the specified text to the specified PrintWriter. |
    | `void` | `printWrapped(PrintWriter pw, int width, String text)` Print the specified text to the specified PrintWriter. |
    | `protected StringBuffer` | `renderOptions(StringBuffer sb, int width, Options options, int leftPad, int descPad)` Render the specified Options and return the rendered Options in a StringBuffer. |
    | `protected StringBuffer` | `renderWrappedText(StringBuffer sb, int width, int nextLineTabStop, String text)` Render the specified text and return the rendered Options in a StringBuffer. |
    | `protected String` | `rtrim(String s)` Remove the trailing whitespace from the specified String. |
    | `void` | `setArgName(String name)` Sets the 'argName'. |
    | `void` | `setDescPadding(int padding)` Sets the 'descPadding'. |
    | `void` | `setLeftPadding(int padding)` Sets the 'leftPadding'. |
    | `void` | `setLongOptPrefix(String prefix)` Sets the 'longOptPrefix'. |
    | `void` | `setLongOptSeparator(String longOptSeparator)` Set the separator displayed between a long option and its value. |
    | `void` | `setNewLine(String newline)` Sets the 'newLine'. |
    | `void` | `setOptionComparator(Comparator<Option> comparator)` Set the comparator used to sort the options when they output in help text. |
    | `void` | `setOptPrefix(String prefix)` Sets the 'optPrefix'. |
    | `void` | `setSyntaxPrefix(String prefix)` Sets the 'syntaxPrefix'. |
    | `void` | `setWidth(int width)` Sets the 'width'. |

    - ### Methods inherited from class java.lang.Object

      `clone, equals, finalize, getClass, hashCode, notify, notifyAll, toString, wait, wait, wait`

- - ### Field Detail


    - #### DEFAULT\_WIDTH

      ```
      public static final int DEFAULT_WIDTH
      ```

      default number of characters per line

      See Also:
      :   Constant Field Values


    - #### DEFAULT\_LEFT\_PAD

      ```
      public static final int DEFAULT_LEFT_PAD
      ```

      default padding to the left of each line

      See Also:
      :   Constant Field Values


    - #### DEFAULT\_DESC\_PAD

      ```
      public static final int DEFAULT_DESC_PAD
      ```

      number of space characters to be prefixed to each description line

      See Also:
      :   Constant Field Values


    - #### DEFAULT\_SYNTAX\_PREFIX

      ```
      public static final String DEFAULT_SYNTAX_PREFIX
      ```

      the string to display at the beginning of the usage statement

      See Also:
      :   Constant Field Values


    - #### DEFAULT\_OPT\_PREFIX

      ```
      public static final String DEFAULT_OPT_PREFIX
      ```

      default prefix for shortOpts

      See Also:
      :   Constant Field Values


    - #### DEFAULT\_LONG\_OPT\_PREFIX

      ```
      public static final String DEFAULT_LONG_OPT_PREFIX
      ```

      default prefix for long Option

      See Also:
      :   Constant Field Values


    - #### DEFAULT\_LONG\_OPT\_SEPARATOR

      ```
      public static final String DEFAULT_LONG_OPT_SEPARATOR
      ```

      default separator displayed between a long Option and its value

      Since:
      :   1.3

      See Also:
      :   Constant Field Values


    - #### DEFAULT\_ARG\_NAME

      ```
      public static final String DEFAULT_ARG_NAME
      ```

      default name for an argument

      See Also:
      :   Constant Field Values


    - #### defaultWidth

      ```
      @Deprecated
      public int defaultWidth
      ```

      Deprecated. Scope will be made private for next major version
      - use get/setWidth methods instead.

      number of characters per line


    - #### defaultLeftPad

      ```
      @Deprecated
      public int defaultLeftPad
      ```

      Deprecated. Scope will be made private for next major version
      - use get/setLeftPadding methods instead.

      amount of padding to the left of each line


    - #### defaultDescPad

      ```
      @Deprecated
      public int defaultDescPad
      ```

      Deprecated. Scope will be made private for next major version
      - use get/setDescPadding methods instead.

      the number of characters of padding to be prefixed
      to each description line


    - #### defaultSyntaxPrefix

      ```
      @Deprecated
      public String defaultSyntaxPrefix
      ```

      Deprecated. Scope will be made private for next major version
      - use get/setSyntaxPrefix methods instead.

      the string to display at the beginning of the usage statement


    - #### defaultNewLine

      ```
      @Deprecated
      public String defaultNewLine
      ```

      Deprecated. Scope will be made private for next major version
      - use get/setNewLine methods instead.

      the new line string


    - #### defaultOptPrefix

      ```
      @Deprecated
      public String defaultOptPrefix
      ```

      Deprecated. Scope will be made private for next major version
      - use get/setOptPrefix methods instead.

      the shortOpt prefix


    - #### defaultLongOptPrefix

      ```
      @Deprecated
      public String defaultLongOptPrefix
      ```

      Deprecated. Scope will be made private for next major version
      - use get/setLongOptPrefix methods instead.

      the long Opt prefix


    - #### defaultArgName

      ```
      @Deprecated
      public String defaultArgName
      ```

      Deprecated. Scope will be made private for next major version
      - use get/setArgName methods instead.

      the name of the argument


    - #### optionComparator

      ```
      protected Comparator<Option> optionComparator
      ```

      Comparator used to sort the options when they output in help text
      Defaults to case-insensitive alphabetical sorting by option key
  - ### Constructor Detail


    - #### HelpFormatter

      ```
      public HelpFormatter()
      ```
  - ### Method Detail


    - #### setWidth

      ```
      public void setWidth(int width)
      ```

      Sets the 'width'.

      Parameters:
      :   `width` - the new value of 'width'


    - #### getWidth

      ```
      public int getWidth()
      ```

      Returns the 'width'.

      Returns:
      :   the 'width'


    - #### setLeftPadding

      ```
      public void setLeftPadding(int padding)
      ```

      Sets the 'leftPadding'.

      Parameters:
      :   `padding` - the new value of 'leftPadding'


    - #### getLeftPadding

      ```
      public int getLeftPadding()
      ```

      Returns the 'leftPadding'.

      Returns:
      :   the 'leftPadding'


    - #### setDescPadding

      ```
      public void setDescPadding(int padding)
      ```

      Sets the 'descPadding'.

      Parameters:
      :   `padding` - the new value of 'descPadding'


    - #### getDescPadding

      ```
      public int getDescPadding()
      ```

      Returns the 'descPadding'.

      Returns:
      :   the 'descPadding'


    - #### setSyntaxPrefix

      ```
      public void setSyntaxPrefix(String prefix)
      ```

      Sets the 'syntaxPrefix'.

      Parameters:
      :   `prefix` - the new value of 'syntaxPrefix'


    - #### getSyntaxPrefix

      ```
      public String getSyntaxPrefix()
      ```

      Returns the 'syntaxPrefix'.

      Returns:
      :   the 'syntaxPrefix'


    - #### setNewLine

      ```
      public void setNewLine(String newline)
      ```

      Sets the 'newLine'.

      Parameters:
      :   `newline` - the new value of 'newLine'


    - #### getNewLine

      ```
      public String getNewLine()
      ```

      Returns the 'newLine'.

      Returns:
      :   the 'newLine'


    - #### setOptPrefix

      ```
      public void setOptPrefix(String prefix)
      ```

      Sets the 'optPrefix'.

      Parameters:
      :   `prefix` - the new value of 'optPrefix'


    - #### getOptPrefix

      ```
      public String getOptPrefix()
      ```

      Returns the 'optPrefix'.

      Returns:
      :   the 'optPrefix'


    - #### setLongOptPrefix

      ```
      public void setLongOptPrefix(String prefix)
      ```

      Sets the 'longOptPrefix'.

      Parameters:
      :   `prefix` - the new value of 'longOptPrefix'


    - #### getLongOptPrefix

      ```
      public String getLongOptPrefix()
      ```

      Returns the 'longOptPrefix'.

      Returns:
      :   the 'longOptPrefix'


    - #### setLongOptSeparator

      ```
      public void setLongOptSeparator(String longOptSeparator)
      ```

      Set the separator displayed between a long option and its value.
      Ensure that the separator specified is supported by the parser used,
      typically ' ' or '='.

      Parameters:
      :   `longOptSeparator` - the separator, typically ' ' or '='.

      Since:
      :   1.3


    - #### getLongOptSeparator

      ```
      public String getLongOptSeparator()
      ```

      Returns the separator displayed between a long option and its value.

      Returns:
      :   the separator

      Since:
      :   1.3


    - #### setArgName

      ```
      public void setArgName(String name)
      ```

      Sets the 'argName'.

      Parameters:
      :   `name` - the new value of 'argName'


    - #### getArgName

      ```
      public String getArgName()
      ```

      Returns the 'argName'.

      Returns:
      :   the 'argName'


    - #### getOptionComparator

      ```
      public Comparator<Option> getOptionComparator()
      ```

      Comparator used to sort the options when they output in help text.
      Defaults to case-insensitive alphabetical sorting by option key.

      Returns:
      :   the `Comparator` currently in use to sort the options

      Since:
      :   1.2


    - #### setOptionComparator

      ```
      public void setOptionComparator(Comparator<Option> comparator)
      ```

      Set the comparator used to sort the options when they output in help text.
      Passing in a null comparator will keep the options in the order they were declared.

      Parameters:
      :   `comparator` - the `Comparator` to use for sorting the options

      Since:
      :   1.2


    - #### printHelp

      ```
      public void printHelp(String cmdLineSyntax,
                            Options options)
      ```

      Print the help for `options` with the specified
      command line syntax. This method prints help information to
      System.out.

      Parameters:
      :   `cmdLineSyntax` - the syntax for this application
      :   `options` - the Options instance


    - #### printHelp

      ```
      public void printHelp(String cmdLineSyntax,
                            Options options,
                            boolean autoUsage)
      ```

      Print the help for `options` with the specified
      command line syntax. This method prints help information to
      System.out.

      Parameters:
      :   `cmdLineSyntax` - the syntax for this application
      :   `options` - the Options instance
      :   `autoUsage` - whether to print an automatically generated
          usage statement


    - #### printHelp

      ```
      public void printHelp(String cmdLineSyntax,
                            String header,
                            Options options,
                            String footer)
      ```

      Print the help for `options` with the specified
      command line syntax. This method prints help information to
      System.out.

      Parameters:
      :   `cmdLineSyntax` - the syntax for this application
      :   `header` - the banner to display at the beginning of the help
      :   `options` - the Options instance
      :   `footer` - the banner to display at the end of the help


    - #### printHelp

      ```
      public void printHelp(String cmdLineSyntax,
                            String header,
                            Options options,
                            String footer,
                            boolean autoUsage)
      ```

      Print the help for `options` with the specified
      command line syntax. This method prints help information to
      System.out.

      Parameters:
      :   `cmdLineSyntax` - the syntax for this application
      :   `header` - the banner to display at the beginning of the help
      :   `options` - the Options instance
      :   `footer` - the banner to display at the end of the help
      :   `autoUsage` - whether to print an automatically generated
          usage statement


    - #### printHelp

      ```
      public void printHelp(int width,
                            String cmdLineSyntax,
                            String header,
                            Options options,
                            String footer)
      ```

      Print the help for `options` with the specified
      command line syntax. This method prints help information to
      System.out.

      Parameters:
      :   `width` - the number of characters to be displayed on each line
      :   `cmdLineSyntax` - the syntax for this application
      :   `header` - the banner to display at the beginning of the help
      :   `options` - the Options instance
      :   `footer` - the banner to display at the end of the help


    - #### printHelp

      ```
      public void printHelp(int width,
                            String cmdLineSyntax,
                            String header,
                            Options options,
                            String footer,
                            boolean autoUsage)
      ```

      Print the help for `options` with the specified
      command line syntax. This method prints help information to
      System.out.

      Parameters:
      :   `width` - the number of characters to be displayed on each line
      :   `cmdLineSyntax` - the syntax for this application
      :   `header` - the banner to display at the beginning of the help
      :   `options` - the Options instance
      :   `footer` - the banner to display at the end of the help
      :   `autoUsage` - whether to print an automatically generated
          usage statement


    - #### printHelp

      ```
      public void printHelp(PrintWriter pw,
                            int width,
                            String cmdLineSyntax,
                            String header,
                            Options options,
                            int leftPad,
                            int descPad,
                            String footer)
      ```

      Print the help for `options` with the specified
      command line syntax.

      Parameters:
      :   `pw` - the writer to which the help will be written
      :   `width` - the number of characters to be displayed on each line
      :   `cmdLineSyntax` - the syntax for this application
      :   `header` - the banner to display at the beginning of the help
      :   `options` - the Options instance
      :   `leftPad` - the number of characters of padding to be prefixed
          to each line
      :   `descPad` - the number of characters of padding to be prefixed
          to each description line
      :   `footer` - the banner to display at the end of the help

      Throws:
      :   `IllegalStateException` - if there is no room to print a line


    - #### printHelp

      ```
      public void printHelp(PrintWriter pw,
                            int width,
                            String cmdLineSyntax,
                            String header,
                            Options options,
                            int leftPad,
                            int descPad,
                            String footer,
                            boolean autoUsage)
      ```

      Print the help for `options` with the specified
      command line syntax.

      Parameters:
      :   `pw` - the writer to which the help will be written
      :   `width` - the number of characters to be displayed on each line
      :   `cmdLineSyntax` - the syntax for this application
      :   `header` - the banner to display at the beginning of the help
      :   `options` - the Options instance
      :   `leftPad` - the number of characters of padding to be prefixed
          to each line
      :   `descPad` - the number of characters of padding to be prefixed
          to each description line
      :   `footer` - the banner to display at the end of the help
      :   `autoUsage` - whether to print an automatically generated
          usage statement

      Throws:
      :   `IllegalStateException` - if there is no room to print a line


    - #### printUsage

      ```
      public void printUsage(PrintWriter pw,
                             int width,
                             String app,
                             Options options)
      ```

      Prints the usage statement for the specified application.

      Parameters:
      :   `pw` - The PrintWriter to print the usage statement
      :   `width` - The number of characters to display per line
      :   `app` - The application name
      :   `options` - The command line Options


    - #### printUsage

      ```
      public void printUsage(PrintWriter pw,
                             int width,
                             String cmdLineSyntax)
      ```

      Print the cmdLineSyntax to the specified writer, using the
      specified width.

      Parameters:
      :   `pw` - The printWriter to write the help to
      :   `width` - The number of characters per line for the usage statement.
      :   `cmdLineSyntax` - The usage statement.


    - #### printOptions

      ```
      public void printOptions(PrintWriter pw,
                               int width,
                               Options options,
                               int leftPad,
                               int descPad)
      ```

      Print the help for the specified Options to the specified writer,
      using the specified width, left padding and description padding.

      Parameters:
      :   `pw` - The printWriter to write the help to
      :   `width` - The number of characters to display per line
      :   `options` - The command line Options
      :   `leftPad` - the number of characters of padding to be prefixed
          to each line
      :   `descPad` - the number of characters of padding to be prefixed
          to each description line


    - #### printWrapped

      ```
      public void printWrapped(PrintWriter pw,
                               int width,
                               String text)
      ```

      Print the specified text to the specified PrintWriter.

      Parameters:
      :   `pw` - The printWriter to write the help to
      :   `width` - The number of characters to display per line
      :   `text` - The text to be written to the PrintWriter


    - #### printWrapped

      ```
      public void printWrapped(PrintWriter pw,
                               int width,
                               int nextLineTabStop,
                               String text)
      ```

      Print the specified text to the specified PrintWriter.

      Parameters:
      :   `pw` - The printWriter to write the help to
      :   `width` - The number of characters to display per line
      :   `nextLineTabStop` - The position on the next line for the first tab.
      :   `text` - The text to be written to the PrintWriter


    - #### renderOptions

      ```
      protected StringBuffer renderOptions(StringBuffer sb,
                                           int width,
                                           Options options,
                                           int leftPad,
                                           int descPad)
      ```

      Render the specified Options and return the rendered Options
      in a StringBuffer.

      Parameters:
      :   `sb` - The StringBuffer to place the rendered Options into.
      :   `width` - The number of characters to display per line
      :   `options` - The command line Options
      :   `leftPad` - the number of characters of padding to be prefixed
          to each line
      :   `descPad` - the number of characters of padding to be prefixed
          to each description line

      Returns:
      :   the StringBuffer with the rendered Options contents.


    - #### renderWrappedText

      ```
      protected StringBuffer renderWrappedText(StringBuffer sb,
                                               int width,
                                               int nextLineTabStop,
                                               String text)
      ```

      Render the specified text and return the rendered Options
      in a StringBuffer.

      Parameters:
      :   `sb` - The StringBuffer to place the rendered text into.
      :   `width` - The number of characters to display per line
      :   `nextLineTabStop` - The position on the next line for the first tab.
      :   `text` - The text to be rendered.

      Returns:
      :   the StringBuffer with the rendered Options contents.


    - #### findWrapPos

      ```
      protected int findWrapPos(String text,
                                int width,
                                int startPos)
      ```

      Finds the next text wrap position after `startPos` for the
      text in `text` with the column width `width`.
      The wrap point is the last position before startPos+width having a
      whitespace character (space, \n, \r). If there is no whitespace character
      before startPos+width, it will return startPos+width.

      Parameters:
      :   `text` - The text being searched for the wrap position
      :   `width` - width of the wrapped text
      :   `startPos` - position from which to start the lookup whitespace
          character

      Returns:
      :   position on which the text must be wrapped or -1 if the wrap
          position is at the end of the text


    - #### createPadding

      ```
      protected String createPadding(int len)
      ```

      Return a String of padding of length `len`.

      Parameters:
      :   `len` - The length of the String of padding to create.

      Returns:
      :   The String of padding


    - #### rtrim

      ```
      protected String rtrim(String s)
      ```

      Remove the trailing whitespace from the specified String.

      Parameters:
      :   `s` - The String to remove the trailing padding from.

      Returns:
      :   The String of without the trailing padding


Skip navigation links


- Package
- Class
- Use
- Tree
- Deprecated
- Index
- Help

- Prev Class
- Next Class

- Frames
- No Frames

- All Classes

- Summary:
- Nested |
- Field |
- Constr |
- Method

- Detail:
- Field |
- Constr |
- Method

Copyright © 2002–2015 The Apache Software Foundation. All rights reserved.
