# Supplementary material for: ACBM: An Integrated Agent and Constraint Based Modeling Framework for Simulation of Microbial Communities
Source: Sci Rep. 2020 May 26;10:8695. doi: 10.1038/s41598-020-65659-w (PMC7250870; doi:10.1038/s41598-020-65659-w)
Supplement: Supplementary file 2 [file 41598_2020_65659_MOESM2_ESM.zip › ACBM1.4/lib/commons-cli-1.3/apidocs/org/apache/commons/cli/Option.Builder.html]

Option.Builder (Apache Commons CLI 1.3 API)


JavaScript is disabled on your browser.


Skip navigation links


- Package
- Class
- Use
- Tree
- Deprecated
- Index
- Help

- Prev Class
- Next Class

- Frames
- No Frames

- All Classes

- Summary:
- Nested |
- Field |
- Constr |
- Method

- Detail:
- Field |
- Constr |
- Method


org.apache.commons.cli

## Class Option.Builder

- java.lang.Object
- - org.apache.commons.cli.Option.Builder

- Enclosing class:
  :   Option

  ---

    

  ```
  public static final class Option.Builder
  extends Object
  ```

  A nested builder class to create `Option` instances
  using descriptive methods.

  Example usage:

  ```
   Option option = Option.builder("a")
       .required(true)
       .longOpt("arg-name")
       .build();
  ```

  Since:
  :   1.3

- - ### Method Summary

    All Methods Instance Methods Concrete Methods

    | Modifier and Type | Method and Description |
    | `Option.Builder` | `argName(String argName)` Sets the display name for the argument value. |
    | `Option` | `build()` Constructs an Option with the values declared by this `Option.Builder`. |
    | `Option.Builder` | `desc(String description)` Sets the description for this option. |
    | `Option.Builder` | `hasArg()` Indicates that the Option will require an argument. |
    | `Option.Builder` | `hasArg(boolean hasArg)` Indicates if the Option has an argument or not. |
    | `Option.Builder` | `hasArgs()` Indicates that the Option can have unlimited argument values. |
    | `Option.Builder` | `longOpt(String longOpt)` Sets the long name of the Option. |
    | `Option.Builder` | `numberOfArgs(int numberOfArgs)` Sets the number of argument values the Option can take. |
    | `Option.Builder` | `optionalArg(boolean isOptional)` Sets whether the Option can have an optional argument. |
    | `Option.Builder` | `required()` Marks this Option as required. |
    | `Option.Builder` | `required(boolean required)` Sets whether the Option is mandatory. |
    | `Option.Builder` | `type(Class<?> type)` Sets the type of the Option. |
    | `Option.Builder` | `valueSeparator()` The Option will use '=' as a means to separate argument value. |
    | `Option.Builder` | `valueSeparator(char sep)` The Option will use `sep` as a means to separate argument values. |

    - ### Methods inherited from class java.lang.Object

      `clone, equals, finalize, getClass, hashCode, notify, notifyAll, toString, wait, wait, wait`

- - ### Method Detail


    - #### argName

      ```
      public Option.Builder argName(String argName)
      ```

      Sets the display name for the argument value.

      Parameters:
      :   `argName` - the display name for the argument value.

      Returns:
      :   this builder, to allow method chaining


    - #### desc

      ```
      public Option.Builder desc(String description)
      ```

      Sets the description for this option.

      Parameters:
      :   `description` - the description of the option.

      Returns:
      :   this builder, to allow method chaining


    - #### longOpt

      ```
      public Option.Builder longOpt(String longOpt)
      ```

      Sets the long name of the Option.

      Parameters:
      :   `longOpt` - the long name of the Option

      Returns:
      :   this builder, to allow method chaining


    - #### numberOfArgs

      ```
      public Option.Builder numberOfArgs(int numberOfArgs)
      ```

      Sets the number of argument values the Option can take.

      Parameters:
      :   `numberOfArgs` - the number of argument values

      Returns:
      :   this builder, to allow method chaining


    - #### optionalArg

      ```
      public Option.Builder optionalArg(boolean isOptional)
      ```

      Sets whether the Option can have an optional argument.

      Parameters:
      :   `isOptional` - specifies whether the Option can have
          an optional argument.

      Returns:
      :   this builder, to allow method chaining


    - #### required

      ```
      public Option.Builder required()
      ```

      Marks this Option as required.

      Returns:
      :   this builder, to allow method chaining


    - #### required

      ```
      public Option.Builder required(boolean required)
      ```

      Sets whether the Option is mandatory.

      Parameters:
      :   `required` - specifies whether the Option is mandatory

      Returns:
      :   this builder, to allow method chaining


    - #### type

      ```
      public Option.Builder type(Class<?> type)
      ```

      Sets the type of the Option.

      Parameters:
      :   `type` - the type of the Option

      Returns:
      :   this builder, to allow method chaining


    - #### valueSeparator

      ```
      public Option.Builder valueSeparator()
      ```

      The Option will use '=' as a means to separate argument value.

      Returns:
      :   this builder, to allow method chaining


    - #### valueSeparator

      ```
      public Option.Builder valueSeparator(char sep)
      ```

      The Option will use `sep` as a means to
      separate argument values.

      **Example:**

      ```
       Option opt = Option.builder("D").hasArgs()
                                       .valueSeparator('=')
                                       .build();
       Options options = new Options();
       options.addOption(opt);
       String[] args = {"-Dkey=value"};
       CommandLineParser parser = new DefaultParser();
       CommandLine line = parser.parse(options, args);
       String propertyName = line.getOptionValues("D")[0];  // will be "key"
       String propertyValue = line.getOptionValues("D")[1]; // will be "value"
      ```

      Parameters:
      :   `sep` - The value separator.

      Returns:
      :   this builder, to allow method chaining


    - #### hasArg

      ```
      public Option.Builder hasArg()
      ```

      Indicates that the Option will require an argument.

      Returns:
      :   this builder, to allow method chaining


    - #### hasArg

      ```
      public Option.Builder hasArg(boolean hasArg)
      ```

      Indicates if the Option has an argument or not.

      Parameters:
      :   `hasArg` - specifies whether the Option takes an argument or not

      Returns:
      :   this builder, to allow method chaining


    - #### hasArgs

      ```
      public Option.Builder hasArgs()
      ```

      Indicates that the Option can have unlimited argument values.

      Returns:
      :   this builder, to allow method chaining


    - #### build

      ```
      public Option build()
      ```

      Constructs an Option with the values declared by this `Option.Builder`.

      Returns:
      :   the new `Option`

      Throws:
      :   `IllegalArgumentException` - if neither `opt` or `longOpt` has been set


Skip navigation links


- Package
- Class
- Use
- Tree
- Deprecated
- Index
- Help

- Prev Class
- Next Class

- Frames
- No Frames

- All Classes

- Summary:
- Nested |
- Field |
- Constr |
- Method

- Detail:
- Field |
- Constr |
- Method

Copyright © 2002–2015 The Apache Software Foundation. All rights reserved.
