# Supplementary material for: ACBM: An Integrated Agent and Constraint Based Modeling Framework for Simulation of Microbial Communities
Source: Sci Rep. 2020 May 26;10:8695. doi: 10.1038/s41598-020-65659-w (PMC7250870; doi:10.1038/s41598-020-65659-w)
Supplement: Supplementary file 2 [file 41598_2020_65659_MOESM2_ESM.zip › ACBM1.4/lib/commons-cli-1.3/apidocs/org/apache/commons/cli/Option.html]

Option (Apache Commons CLI 1.3 API)


JavaScript is disabled on your browser.


Skip navigation links


- Package
- Class
- Use
- Tree
- Deprecated
- Index
- Help

- Prev Class
- Next Class

- Frames
- No Frames

- All Classes

- Summary:
- Nested |
- Field |
- Constr |
- Method

- Detail:
- Field |
- Constr |
- Method


org.apache.commons.cli

## Class Option

- java.lang.Object
- - org.apache.commons.cli.Option

- All Implemented Interfaces:
  :   Serializable, Cloneable

  ---

    

  ```
  public class Option
  extends Object
  implements Cloneable, Serializable
  ```

  Describes a single command-line option. It maintains
  information regarding the short-name of the option, the long-name,
  if any exists, a flag indicating if an argument is required for
  this option, and a self-documenting description of the option.

  An Option is not created independently, but is created through
  an instance of `Options`. An Option is required to have
  at least a short or a long-name.

  **Note:** once an `Option` has been added to an instance
  of `Options`, it's required flag may not be changed anymore.

  Version:
  :   $Id: Option.java 1677406 2015-05-03 14:27:31Z britter $

  See Also:
  :   `Options`,
      `CommandLine`,
      Serialized Form

- - ### Nested Class Summary

    Nested Classes

    | Modifier and Type | Class and Description |
    | `static class` | `Option.Builder` A nested builder class to create `Option` instances using descriptive methods. |
  - ### Field Summary

    Fields

    | Modifier and Type | Field and Description |
    | `static int` | `UNINITIALIZED` constant that specifies the number of argument values has not been specified |
    | `static int` | `UNLIMITED_VALUES` constant that specifies the number of argument values is infinite |
  - ### Constructor Summary

    Constructors

    | Constructor and Description |
    | `Option(String opt, boolean hasArg, String description)` Creates an Option using the specified parameters. |
    | `Option(String opt, String description)` Creates an Option using the specified parameters. |
    | `Option(String opt, String longOpt, boolean hasArg, String description)` Creates an Option using the specified parameters. |
  - ### Method Summary

    All Methods Static Methods Instance Methods Concrete Methods Deprecated Methods

    | Modifier and Type | Method and Description |
    | `boolean` | `addValue(String value)` Deprecated. |
    | `static Option.Builder` | `builder()` Returns a `Option.Builder` to create an `Option` using descriptive methods. |
    | `static Option.Builder` | `builder(String opt)` Returns a `Option.Builder` to create an `Option` using descriptive methods. |
    | `Object` | `clone()` A rather odd clone method - due to incorrect code in 1.0 it is public and in 1.1 rather than throwing a CloneNotSupportedException it throws a RuntimeException so as to maintain backwards compat at the API level. |
    | `boolean` | `equals(Object o)` |
    | `String` | `getArgName()` Gets the display name for the argument value. |
    | `int` | `getArgs()` Returns the number of argument values this Option can take. |
    | `String` | `getDescription()` Retrieve the self-documenting description of this Option |
    | `int` | `getId()` Returns the id of this Option. |
    | `String` | `getLongOpt()` Retrieve the long name of this Option. |
    | `String` | `getOpt()` Retrieve the name of this Option. |
    | `Object` | `getType()` Retrieve the type of this Option. |
    | `String` | `getValue()` Returns the specified value of this Option or `null` if there is no value. |
    | `String` | `getValue(int index)` Returns the specified value of this Option or `null` if there is no value. |
    | `String` | `getValue(String defaultValue)` Returns the value/first value of this Option or the `defaultValue` if there is no value. |
    | `String[]` | `getValues()` Return the values of this Option as a String array or null if there are no values |
    | `char` | `getValueSeparator()` Returns the value separator character. |
    | `List<String>` | `getValuesList()` |
    | `boolean` | `hasArg()` Query to see if this Option requires an argument |
    | `boolean` | `hasArgName()` Returns whether the display name for the argument value has been set. |
    | `boolean` | `hasArgs()` Query to see if this Option can take many values. |
    | `int` | `hashCode()` |
    | `boolean` | `hasLongOpt()` Query to see if this Option has a long name |
    | `boolean` | `hasOptionalArg()` |
    | `boolean` | `hasValueSeparator()` Return whether this Option has specified a value separator. |
    | `boolean` | `isRequired()` Query to see if this Option is mandatory |
    | `void` | `setArgName(String argName)` Sets the display name for the argument value. |
    | `void` | `setArgs(int num)` Sets the number of argument values this Option can take. |
    | `void` | `setDescription(String description)` Sets the self-documenting description of this Option |
    | `void` | `setLongOpt(String longOpt)` Sets the long name of this Option. |
    | `void` | `setOptionalArg(boolean optionalArg)` Sets whether this Option can have an optional argument. |
    | `void` | `setRequired(boolean required)` Sets whether this Option is mandatory. |
    | `void` | `setType(Class<?> type)` Sets the type of this Option. |
    | `void` | `setType(Object type)` Deprecated. since 1.3, use `setType(Class)` instead |
    | `void` | `setValueSeparator(char sep)` Sets the value separator. |
    | `String` | `toString()` Dump state, suitable for debugging. |

    - ### Methods inherited from class java.lang.Object

      `finalize, getClass, notify, notifyAll, wait, wait, wait`

- - ### Field Detail


    - #### UNINITIALIZED

      ```
      public static final int UNINITIALIZED
      ```

      constant that specifies the number of argument values has not been specified

      See Also:
      :   Constant Field Values


    - #### UNLIMITED\_VALUES

      ```
      public static final int UNLIMITED_VALUES
      ```

      constant that specifies the number of argument values is infinite

      See Also:
      :   Constant Field Values
  - ### Constructor Detail


    - #### Option

      ```
      public Option(String opt,
                    String description)
             throws IllegalArgumentException
      ```

      Creates an Option using the specified parameters.
      The option does not take an argument.

      Parameters:
      :   `opt` - short representation of the option
      :   `description` - describes the function of the option

      Throws:
      :   `IllegalArgumentException` - if there are any non valid
          Option characters in `opt`.


    - #### Option

      ```
      public Option(String opt,
                    boolean hasArg,
                    String description)
             throws IllegalArgumentException
      ```

      Creates an Option using the specified parameters.

      Parameters:
      :   `opt` - short representation of the option
      :   `hasArg` - specifies whether the Option takes an argument or not
      :   `description` - describes the function of the option

      Throws:
      :   `IllegalArgumentException` - if there are any non valid
          Option characters in `opt`.


    - #### Option

      ```
      public Option(String opt,
                    String longOpt,
                    boolean hasArg,
                    String description)
             throws IllegalArgumentException
      ```

      Creates an Option using the specified parameters.

      Parameters:
      :   `opt` - short representation of the option
      :   `longOpt` - the long representation of the option
      :   `hasArg` - specifies whether the Option takes an argument or not
      :   `description` - describes the function of the option

      Throws:
      :   `IllegalArgumentException` - if there are any non valid
          Option characters in `opt`.
  - ### Method Detail


    - #### getId

      ```
      public int getId()
      ```

      Returns the id of this Option. This is only set when the
      Option shortOpt is a single character. This is used for switch
      statements.

      Returns:
      :   the id of this Option


    - #### getOpt

      ```
      public String getOpt()
      ```

      Retrieve the name of this Option.
      It is this String which can be used with
      `CommandLine.hasOption(String opt)` and
      `CommandLine.getOptionValue(String opt)` to check
      for existence and argument.

      Returns:
      :   The name of this option


    - #### getType

      ```
      public Object getType()
      ```

      Retrieve the type of this Option.

      Returns:
      :   The type of this option


    - #### setType

      ```
      @Deprecated
      public void setType(Object type)
      ```

      Deprecated. since 1.3, use `setType(Class)` instead

      Sets the type of this Option.

      **Note:** this method is kept for binary compatibility and the
      input type is supposed to be a `Class` object.

      Parameters:
      :   `type` - the type of this Option


    - #### setType

      ```
      public void setType(Class<?> type)
      ```

      Sets the type of this Option.

      Parameters:
      :   `type` - the type of this Option

      Since:
      :   1.3


    - #### getLongOpt

      ```
      public String getLongOpt()
      ```

      Retrieve the long name of this Option.

      Returns:
      :   Long name of this option, or null, if there is no long name


    - #### setLongOpt

      ```
      public void setLongOpt(String longOpt)
      ```

      Sets the long name of this Option.

      Parameters:
      :   `longOpt` - the long name of this Option


    - #### setOptionalArg

      ```
      public void setOptionalArg(boolean optionalArg)
      ```

      Sets whether this Option can have an optional argument.

      Parameters:
      :   `optionalArg` - specifies whether the Option can have
          an optional argument.


    - #### hasOptionalArg

      ```
      public boolean hasOptionalArg()
      ```

      Returns:
      :   whether this Option can have an optional argument


    - #### hasLongOpt

      ```
      public boolean hasLongOpt()
      ```

      Query to see if this Option has a long name

      Returns:
      :   boolean flag indicating existence of a long name


    - #### hasArg

      ```
      public boolean hasArg()
      ```

      Query to see if this Option requires an argument

      Returns:
      :   boolean flag indicating if an argument is required


    - #### getDescription

      ```
      public String getDescription()
      ```

      Retrieve the self-documenting description of this Option

      Returns:
      :   The string description of this option


    - #### setDescription

      ```
      public void setDescription(String description)
      ```

      Sets the self-documenting description of this Option

      Parameters:
      :   `description` - The description of this option

      Since:
      :   1.1


    - #### isRequired

      ```
      public boolean isRequired()
      ```

      Query to see if this Option is mandatory

      Returns:
      :   boolean flag indicating whether this Option is mandatory


    - #### setRequired

      ```
      public void setRequired(boolean required)
      ```

      Sets whether this Option is mandatory.

      Parameters:
      :   `required` - specifies whether this Option is mandatory


    - #### setArgName

      ```
      public void setArgName(String argName)
      ```

      Sets the display name for the argument value.

      Parameters:
      :   `argName` - the display name for the argument value.


    - #### getArgName

      ```
      public String getArgName()
      ```

      Gets the display name for the argument value.

      Returns:
      :   the display name for the argument value.


    - #### hasArgName

      ```
      public boolean hasArgName()
      ```

      Returns whether the display name for the argument value has been set.

      Returns:
      :   if the display name for the argument value has been set.


    - #### hasArgs

      ```
      public boolean hasArgs()
      ```

      Query to see if this Option can take many values.

      Returns:
      :   boolean flag indicating if multiple values are allowed


    - #### setArgs

      ```
      public void setArgs(int num)
      ```

      Sets the number of argument values this Option can take.

      Parameters:
      :   `num` - the number of argument values


    - #### setValueSeparator

      ```
      public void setValueSeparator(char sep)
      ```

      Sets the value separator. For example if the argument value
      was a Java property, the value separator would be '='.

      Parameters:
      :   `sep` - The value separator.


    - #### getValueSeparator

      ```
      public char getValueSeparator()
      ```

      Returns the value separator character.

      Returns:
      :   the value separator character.


    - #### hasValueSeparator

      ```
      public boolean hasValueSeparator()
      ```

      Return whether this Option has specified a value separator.

      Returns:
      :   whether this Option has specified a value separator.

      Since:
      :   1.1


    - #### getArgs

      ```
      public int getArgs()
      ```

      Returns the number of argument values this Option can take.

      Returns:
      :   num the number of argument values


    - #### getValue

      ```
      public String getValue()
      ```

      Returns the specified value of this Option or
      `null` if there is no value.

      Returns:
      :   the value/first value of this Option or
          `null` if there is no value.


    - #### getValue

      ```
      public String getValue(int index)
                      throws IndexOutOfBoundsException
      ```

      Returns the specified value of this Option or
      `null` if there is no value.

      Parameters:
      :   `index` - The index of the value to be returned.

      Returns:
      :   the specified value of this Option or
          `null` if there is no value.

      Throws:
      :   `IndexOutOfBoundsException` - if index is less than 1
          or greater than the number of the values for this Option.


    - #### getValue

      ```
      public String getValue(String defaultValue)
      ```

      Returns the value/first value of this Option or the
      `defaultValue` if there is no value.

      Parameters:
      :   `defaultValue` - The value to be returned if there
          is no value.

      Returns:
      :   the value/first value of this Option or the
          `defaultValue` if there are no values.


    - #### getValues

      ```
      public String[] getValues()
      ```

      Return the values of this Option as a String array
      or null if there are no values

      Returns:
      :   the values of this Option as a String array
          or null if there are no values


    - #### getValuesList

      ```
      public List<String> getValuesList()
      ```

      Returns:
      :   the values of this Option as a List
          or null if there are no values


    - #### toString

      ```
      public String toString()
      ```

      Dump state, suitable for debugging.

      Overrides:
      :   `toString` in class `Object`

      Returns:
      :   Stringified form of this object


    - #### equals

      ```
      public boolean equals(Object o)
      ```

      Overrides:
      :   `equals` in class `Object`


    - #### hashCode

      ```
      public int hashCode()
      ```

      Overrides:
      :   `hashCode` in class `Object`


    - #### clone

      ```
      public Object clone()
      ```

      A rather odd clone method - due to incorrect code in 1.0 it is public
      and in 1.1 rather than throwing a CloneNotSupportedException it throws
      a RuntimeException so as to maintain backwards compat at the API level.
      After calling this method, it is very likely you will want to call
      clearValues().

      Overrides:
      :   `clone` in class `Object`

      Returns:
      :   a clone of this Option instance

      Throws:
      :   `RuntimeException` - if a `CloneNotSupportedException` has been thrown
          by `super.clone()`


    - #### addValue

      ```
      @Deprecated
      public boolean addValue(String value)
      ```

      Deprecated.

      This method is not intended to be used. It was a piece of internal
      API that was made public in 1.0. It currently throws an UnsupportedOperationException.

      Parameters:
      :   `value` - the value to add

      Returns:
      :   always throws an `UnsupportedOperationException`

      Throws:
      :   `UnsupportedOperationException` - always


    - #### builder

      ```
      public static Option.Builder builder()
      ```

      Returns a `Option.Builder` to create an `Option` using descriptive
      methods.

      Returns:
      :   a new `Option.Builder` instance

      Since:
      :   1.3


    - #### builder

      ```
      public static Option.Builder builder(String opt)
      ```

      Returns a `Option.Builder` to create an `Option` using descriptive
      methods.

      Parameters:
      :   `opt` - short representation of the option

      Returns:
      :   a new `Option.Builder` instance

      Throws:
      :   `IllegalArgumentException` - if there are any non valid Option characters in `opt`

      Since:
      :   1.3


Skip navigation links


- Package
- Class
- Use
- Tree
- Deprecated
- Index
- Help

- Prev Class
- Next Class

- Frames
- No Frames

- All Classes

- Summary:
- Nested |
- Field |
- Constr |
- Method

- Detail:
- Field |
- Constr |
- Method

Copyright © 2002–2015 The Apache Software Foundation. All rights reserved.
