# Supplementary material for: ACBM: An Integrated Agent and Constraint Based Modeling Framework for Simulation of Microbial Communities
Source: Sci Rep. 2020 May 26;10:8695. doi: 10.1038/s41598-020-65659-w (PMC7250870; doi:10.1038/s41598-020-65659-w)
Supplement: Supplementary file 2 [file 41598_2020_65659_MOESM2_ESM.zip › ACBM1.4/lib/commons-cli-1.3/apidocs/org/apache/commons/cli/OptionBuilder.html]

OptionBuilder (Apache Commons CLI 1.3 API)


JavaScript is disabled on your browser.


Skip navigation links


- Package
- Class
- Use
- Tree
- Deprecated
- Index
- Help

- Prev Class
- Next Class

- Frames
- No Frames

- All Classes

- Summary:
- Nested |
- Field |
- Constr |
- Method

- Detail:
- Field |
- Constr |
- Method


org.apache.commons.cli

## Class OptionBuilder

- java.lang.Object
- - org.apache.commons.cli.OptionBuilder

- ---

  Deprecated.

  since 1.3, use `Option.builder(String)` instead

    

  ```
  @Deprecated
  public final class OptionBuilder
  extends Object
  ```

  OptionBuilder allows the user to create Options using descriptive methods.

  Details on the Builder pattern can be found at
  http://c2.com/cgi-bin/wiki?BuilderPattern.

  This class is NOT thread safe. See CLI-209

  Since:
  :   1.0

  Version:
  :   $Id: OptionBuilder.java 1677400 2015-05-03 13:46:08Z britter $

- - ### Method Summary

    All Methods Static Methods Concrete Methods Deprecated Methods

    | Modifier and Type | Method and Description |
    | `static Option` | `create()` Deprecated.  Create an Option using the current settings |
    | `static Option` | `create(char opt)` Deprecated.  Create an Option using the current settings and with the specified Option `char`. |
    | `static Option` | `create(String opt)` Deprecated.  Create an Option using the current settings and with the specified Option `char`. |
    | `static OptionBuilder` | `hasArg()` Deprecated.  The next Option created will require an argument value. |
    | `static OptionBuilder` | `hasArg(boolean hasArg)` Deprecated.  The next Option created will require an argument value if `hasArg` is true. |
    | `static OptionBuilder` | `hasArgs()` Deprecated.  The next Option created can have unlimited argument values. |
    | `static OptionBuilder` | `hasArgs(int num)` Deprecated.  The next Option created can have `num` argument values. |
    | `static OptionBuilder` | `hasOptionalArg()` Deprecated.  The next Option can have an optional argument. |
    | `static OptionBuilder` | `hasOptionalArgs()` Deprecated.  The next Option can have an unlimited number of optional arguments. |
    | `static OptionBuilder` | `hasOptionalArgs(int numArgs)` Deprecated.  The next Option can have the specified number of optional arguments. |
    | `static OptionBuilder` | `isRequired()` Deprecated.  The next Option created will be required. |
    | `static OptionBuilder` | `isRequired(boolean newRequired)` Deprecated.  The next Option created will be required if `required` is true. |
    | `static OptionBuilder` | `withArgName(String name)` Deprecated.  The next Option created will have the specified argument value name. |
    | `static OptionBuilder` | `withDescription(String newDescription)` Deprecated.  The next Option created will have the specified description |
    | `static OptionBuilder` | `withLongOpt(String newLongopt)` Deprecated.  The next Option created will have the following long option value. |
    | `static OptionBuilder` | `withType(Class<?> newType)` Deprecated.  The next Option created will have a value that will be an instance of `type`. |
    | `static OptionBuilder` | `withType(Object newType)` Deprecated. since 1.3, use `withType(Class)` instead |
    | `static OptionBuilder` | `withValueSeparator()` Deprecated.  The next Option created uses '`=`' as a means to separate argument values. |
    | `static OptionBuilder` | `withValueSeparator(char sep)` Deprecated.  The next Option created uses `sep` as a means to separate argument values. |

    - ### Methods inherited from class java.lang.Object

      `clone, equals, finalize, getClass, hashCode, notify, notifyAll, toString, wait, wait, wait`

- - ### Method Detail


    - #### withLongOpt

      ```
      public static OptionBuilder withLongOpt(String newLongopt)
      ```

      Deprecated.

      The next Option created will have the following long option value.

      Parameters:
      :   `newLongopt` - the long option value

      Returns:
      :   the OptionBuilder instance


    - #### hasArg

      ```
      public static OptionBuilder hasArg()
      ```

      Deprecated.

      The next Option created will require an argument value.

      Returns:
      :   the OptionBuilder instance


    - #### hasArg

      ```
      public static OptionBuilder hasArg(boolean hasArg)
      ```

      Deprecated.

      The next Option created will require an argument value if
      `hasArg` is true.

      Parameters:
      :   `hasArg` - if true then the Option has an argument value

      Returns:
      :   the OptionBuilder instance


    - #### withArgName

      ```
      public static OptionBuilder withArgName(String name)
      ```

      Deprecated.

      The next Option created will have the specified argument value name.

      Parameters:
      :   `name` - the name for the argument value

      Returns:
      :   the OptionBuilder instance


    - #### isRequired

      ```
      public static OptionBuilder isRequired()
      ```

      Deprecated.

      The next Option created will be required.

      Returns:
      :   the OptionBuilder instance


    - #### withValueSeparator

      ```
      public static OptionBuilder withValueSeparator(char sep)
      ```

      Deprecated.

      The next Option created uses `sep` as a means to
      separate argument values.

      **Example:**

      ```
       Option opt = OptionBuilder.withValueSeparator('=')
                                 .create('D');

       String args = "-Dkey=value";
       CommandLine line = parser.parse(args);
       String propertyName = opt.getValue(0);  // will be "key"
       String propertyValue = opt.getValue(1); // will be "value"
      ```

      Parameters:
      :   `sep` - The value separator to be used for the argument values.

      Returns:
      :   the OptionBuilder instance


    - #### withValueSeparator

      ```
      public static OptionBuilder withValueSeparator()
      ```

      Deprecated.

      The next Option created uses '`=`' as a means to
      separate argument values.
      **Example:**

      ```
       Option opt = OptionBuilder.withValueSeparator()
                                 .create('D');

       CommandLine line = parser.parse(args);
       String propertyName = opt.getValue(0);
       String propertyValue = opt.getValue(1);
      ```

      Returns:
      :   the OptionBuilder instance


    - #### isRequired

      ```
      public static OptionBuilder isRequired(boolean newRequired)
      ```

      Deprecated.

      The next Option created will be required if `required`
      is true.

      Parameters:
      :   `newRequired` - if true then the Option is required

      Returns:
      :   the OptionBuilder instance


    - #### hasArgs

      ```
      public static OptionBuilder hasArgs()
      ```

      Deprecated.

      The next Option created can have unlimited argument values.

      Returns:
      :   the OptionBuilder instance


    - #### hasArgs

      ```
      public static OptionBuilder hasArgs(int num)
      ```

      Deprecated.

      The next Option created can have `num` argument values.

      Parameters:
      :   `num` - the number of args that the option can have

      Returns:
      :   the OptionBuilder instance


    - #### hasOptionalArg

      ```
      public static OptionBuilder hasOptionalArg()
      ```

      Deprecated.

      The next Option can have an optional argument.

      Returns:
      :   the OptionBuilder instance


    - #### hasOptionalArgs

      ```
      public static OptionBuilder hasOptionalArgs()
      ```

      Deprecated.

      The next Option can have an unlimited number of optional arguments.

      Returns:
      :   the OptionBuilder instance


    - #### hasOptionalArgs

      ```
      public static OptionBuilder hasOptionalArgs(int numArgs)
      ```

      Deprecated.

      The next Option can have the specified number of optional arguments.

      Parameters:
      :   `numArgs` - - the maximum number of optional arguments
          the next Option created can have.

      Returns:
      :   the OptionBuilder instance


    - #### withType

      ```
      @Deprecated
      public static OptionBuilder withType(Object newType)
      ```

      Deprecated. since 1.3, use `withType(Class)` instead

      The next Option created will have a value that will be an instance
      of `type`.

      **Note:** this method is kept for binary compatibility and the
      input type is supposed to be a `Class` object.

      Parameters:
      :   `newType` - the type of the Options argument value

      Returns:
      :   the OptionBuilder instance


    - #### withType

      ```
      public static OptionBuilder withType(Class<?> newType)
      ```

      Deprecated.

      The next Option created will have a value that will be an instance
      of `type`.

      Parameters:
      :   `newType` - the type of the Options argument value

      Returns:
      :   the OptionBuilder instance

      Since:
      :   1.3


    - #### withDescription

      ```
      public static OptionBuilder withDescription(String newDescription)
      ```

      Deprecated.

      The next Option created will have the specified description

      Parameters:
      :   `newDescription` - a description of the Option's purpose

      Returns:
      :   the OptionBuilder instance


    - #### create

      ```
      public static Option create(char opt)
                           throws IllegalArgumentException
      ```

      Deprecated.

      Create an Option using the current settings and with
      the specified Option `char`.

      Parameters:
      :   `opt` - the character representation of the Option

      Returns:
      :   the Option instance

      Throws:
      :   `IllegalArgumentException` - if `opt` is not
          a valid character. See Option.


    - #### create

      ```
      public static Option create()
                           throws IllegalArgumentException
      ```

      Deprecated.

      Create an Option using the current settings

      Returns:
      :   the Option instance

      Throws:
      :   `IllegalArgumentException` - if `longOpt` has not been set.


    - #### create

      ```
      public static Option create(String opt)
                           throws IllegalArgumentException
      ```

      Deprecated.

      Create an Option using the current settings and with
      the specified Option `char`.

      Parameters:
      :   `opt` - the `java.lang.String` representation
          of the Option

      Returns:
      :   the Option instance

      Throws:
      :   `IllegalArgumentException` - if `opt` is not
          a valid character. See Option.


Skip navigation links


- Package
- Class
- Use
- Tree
- Deprecated
- Index
- Help

- Prev Class
- Next Class

- Frames
- No Frames

- All Classes

- Summary:
- Nested |
- Field |
- Constr |
- Method

- Detail:
- Field |
- Constr |
- Method

Copyright © 2002–2015 The Apache Software Foundation. All rights reserved.
