# Supplementary material for: ACBM: An Integrated Agent and Constraint Based Modeling Framework for Simulation of Microbial Communities
Source: Sci Rep. 2020 May 26;10:8695. doi: 10.1038/s41598-020-65659-w (PMC7250870; doi:10.1038/s41598-020-65659-w)
Supplement: Supplementary file 2 [file 41598_2020_65659_MOESM2_ESM.zip › ACBM1.4/lib/commons-cli-1.3/apidocs/org/apache/commons/cli/OptionGroup.html]

OptionGroup (Apache Commons CLI 1.3 API)


JavaScript is disabled on your browser.


Skip navigation links


- Package
- Class
- Use
- Tree
- Deprecated
- Index
- Help

- Prev Class
- Next Class

- Frames
- No Frames

- All Classes

- Summary:
- Nested |
- Field |
- Constr |
- Method

- Detail:
- Field |
- Constr |
- Method


org.apache.commons.cli

## Class OptionGroup

- java.lang.Object
- - org.apache.commons.cli.OptionGroup

- All Implemented Interfaces:
  :   Serializable

  ---

    

  ```
  public class OptionGroup
  extends Object
  implements Serializable
  ```

  A group of mutually exclusive options.

  Version:
  :   $Id: OptionGroup.java 1669814 2015-03-28 18:09:26Z britter $

  See Also:
  :   Serialized Form

- - ### Constructor Summary

    Constructors

    | Constructor and Description |
    | `OptionGroup()` |
  - ### Method Summary

    All Methods Instance Methods Concrete Methods

    | Modifier and Type | Method and Description |
    | `OptionGroup` | `addOption(Option option)` Add the specified `Option` to this group. |
    | `Collection<String>` | `getNames()` |
    | `Collection<Option>` | `getOptions()` |
    | `String` | `getSelected()` |
    | `boolean` | `isRequired()` Returns whether this option group is required. |
    | `void` | `setRequired(boolean required)` |
    | `void` | `setSelected(Option option)` Set the selected option of this group to `name`. |
    | `String` | `toString()` Returns the stringified version of this OptionGroup. |

    - ### Methods inherited from class java.lang.Object

      `clone, equals, finalize, getClass, hashCode, notify, notifyAll, wait, wait, wait`

- - ### Constructor Detail


    - #### OptionGroup

      ```
      public OptionGroup()
      ```
  - ### Method Detail


    - #### addOption

      ```
      public OptionGroup addOption(Option option)
      ```

      Add the specified `Option` to this group.

      Parameters:
      :   `option` - the option to add to this group

      Returns:
      :   this option group with the option added


    - #### getNames

      ```
      public Collection<String> getNames()
      ```

      Returns:
      :   the names of the options in this group as a
          `Collection`


    - #### getOptions

      ```
      public Collection<Option> getOptions()
      ```

      Returns:
      :   the options in this group as a `Collection`


    - #### setSelected

      ```
      public void setSelected(Option option)
                       throws AlreadySelectedException
      ```

      Set the selected option of this group to `name`.

      Parameters:
      :   `option` - the option that is selected

      Throws:
      :   `AlreadySelectedException` - if an option from this group has
          already been selected.


    - #### getSelected

      ```
      public String getSelected()
      ```

      Returns:
      :   the selected option name


    - #### setRequired

      ```
      public void setRequired(boolean required)
      ```

      Parameters:
      :   `required` - specifies if this group is required


    - #### isRequired

      ```
      public boolean isRequired()
      ```

      Returns whether this option group is required.

      Returns:
      :   whether this option group is required


    - #### toString

      ```
      public String toString()
      ```

      Returns the stringified version of this OptionGroup.

      Overrides:
      :   `toString` in class `Object`

      Returns:
      :   the stringified representation of this group


Skip navigation links


- Package
- Class
- Use
- Tree
- Deprecated
- Index
- Help

- Prev Class
- Next Class

- Frames
- No Frames

- All Classes

- Summary:
- Nested |
- Field |
- Constr |
- Method

- Detail:
- Field |
- Constr |
- Method

Copyright © 2002–2015 The Apache Software Foundation. All rights reserved.
