# Supplementary material for: ACBM: An Integrated Agent and Constraint Based Modeling Framework for Simulation of Microbial Communities
Source: Sci Rep. 2020 May 26;10:8695. doi: 10.1038/s41598-020-65659-w (PMC7250870; doi:10.1038/s41598-020-65659-w)
Supplement: Supplementary file 2 [file 41598_2020_65659_MOESM2_ESM.zip › ACBM1.4/lib/commons-cli-1.3/apidocs/org/apache/commons/cli/Options.html]

Options (Apache Commons CLI 1.3 API)


JavaScript is disabled on your browser.


Skip navigation links


- Package
- Class
- Use
- Tree
- Deprecated
- Index
- Help

- Prev Class
- Next Class

- Frames
- No Frames

- All Classes

- Summary:
- Nested |
- Field |
- Constr |
- Method

- Detail:
- Field |
- Constr |
- Method


org.apache.commons.cli

## Class Options

- java.lang.Object
- - org.apache.commons.cli.Options

- All Implemented Interfaces:
  :   Serializable

  ---

    

  ```
  public class Options
  extends Object
  implements Serializable
  ```

  Main entry-point into the library.

  Options represents a collection of `Option` objects, which
  describe the possible options for a command-line.

  It may flexibly parse long and short options, with or without
  values. Additionally, it may parse only a portion of a commandline,
  allowing for flexible multi-stage parsing.

  Version:
  :   $Id: Options.java 1669814 2015-03-28 18:09:26Z britter $

  See Also:
  :   `CommandLine`,
      Serialized Form

- - ### Constructor Summary

    Constructors

    | Constructor and Description |
    | `Options()` |
  - ### Method Summary

    All Methods Instance Methods Concrete Methods

    | Modifier and Type | Method and Description |
    | `Options` | `addOption(Option opt)` Adds an option instance |
    | `Options` | `addOption(String opt, boolean hasArg, String description)` Add an option that only contains a short-name. |
    | `Options` | `addOption(String opt, String description)` Add an option that only contains a short name. |
    | `Options` | `addOption(String opt, String longOpt, boolean hasArg, String description)` Add an option that contains a short-name and a long-name. |
    | `Options` | `addOptionGroup(OptionGroup group)` Add the specified option group. |
    | `List<String>` | `getMatchingOptions(String opt)` Returns the options with a long name starting with the name specified. |
    | `Option` | `getOption(String opt)` Retrieve the `Option` matching the long or short name specified. |
    | `OptionGroup` | `getOptionGroup(Option opt)` Returns the OptionGroup the `opt` belongs to. |
    | `Collection<Option>` | `getOptions()` Retrieve a read-only list of options in this set |
    | `List` | `getRequiredOptions()` Returns the required options. |
    | `boolean` | `hasLongOption(String opt)` Returns whether the named `Option` is a member of this `Options`. |
    | `boolean` | `hasOption(String opt)` Returns whether the named `Option` is a member of this `Options`. |
    | `boolean` | `hasShortOption(String opt)` Returns whether the named `Option` is a member of this `Options`. |
    | `String` | `toString()` Dump state, suitable for debugging. |

    - ### Methods inherited from class java.lang.Object

      `clone, equals, finalize, getClass, hashCode, notify, notifyAll, wait, wait, wait`

- - ### Constructor Detail


    - #### Options

      ```
      public Options()
      ```
  - ### Method Detail


    - #### addOptionGroup

      ```
      public Options addOptionGroup(OptionGroup group)
      ```

      Add the specified option group.

      Parameters:
      :   `group` - the OptionGroup that is to be added

      Returns:
      :   the resulting Options instance


    - #### addOption

      ```
      public Options addOption(String opt,
                               String description)
      ```

      Add an option that only contains a short name.
      The option does not take an argument.

      Parameters:
      :   `opt` - Short single-character name of the option.
      :   `description` - Self-documenting description

      Returns:
      :   the resulting Options instance

      Since:
      :   1.3


    - #### addOption

      ```
      public Options addOption(String opt,
                               boolean hasArg,
                               String description)
      ```

      Add an option that only contains a short-name.
      It may be specified as requiring an argument.

      Parameters:
      :   `opt` - Short single-character name of the option.
      :   `hasArg` - flag signally if an argument is required after this option
      :   `description` - Self-documenting description

      Returns:
      :   the resulting Options instance


    - #### addOption

      ```
      public Options addOption(String opt,
                               String longOpt,
                               boolean hasArg,
                               String description)
      ```

      Add an option that contains a short-name and a long-name.
      It may be specified as requiring an argument.

      Parameters:
      :   `opt` - Short single-character name of the option.
      :   `longOpt` - Long multi-character name of the option.
      :   `hasArg` - flag signally if an argument is required after this option
      :   `description` - Self-documenting description

      Returns:
      :   the resulting Options instance


    - #### addOption

      ```
      public Options addOption(Option opt)
      ```

      Adds an option instance

      Parameters:
      :   `opt` - the option that is to be added

      Returns:
      :   the resulting Options instance


    - #### getOptions

      ```
      public Collection<Option> getOptions()
      ```

      Retrieve a read-only list of options in this set

      Returns:
      :   read-only Collection of `Option` objects in this descriptor


    - #### getRequiredOptions

      ```
      public List getRequiredOptions()
      ```

      Returns the required options.

      Returns:
      :   read-only List of required options


    - #### getOption

      ```
      public Option getOption(String opt)
      ```

      Retrieve the `Option` matching the long or short name specified.
      The leading hyphens in the name are ignored (up to 2).

      Parameters:
      :   `opt` - short or long name of the `Option`

      Returns:
      :   the option represented by opt


    - #### getMatchingOptions

      ```
      public List<String> getMatchingOptions(String opt)
      ```

      Returns the options with a long name starting with the name specified.

      Parameters:
      :   `opt` - the partial name of the option

      Returns:
      :   the options matching the partial name specified, or an empty list if none matches

      Since:
      :   1.3


    - #### hasOption

      ```
      public boolean hasOption(String opt)
      ```

      Returns whether the named `Option` is a member of this `Options`.

      Parameters:
      :   `opt` - short or long name of the `Option`

      Returns:
      :   true if the named `Option` is a member of this `Options`


    - #### hasLongOption

      ```
      public boolean hasLongOption(String opt)
      ```

      Returns whether the named `Option` is a member of this `Options`.

      Parameters:
      :   `opt` - long name of the `Option`

      Returns:
      :   true if the named `Option` is a member of this `Options`

      Since:
      :   1.3


    - #### hasShortOption

      ```
      public boolean hasShortOption(String opt)
      ```

      Returns whether the named `Option` is a member of this `Options`.

      Parameters:
      :   `opt` - short name of the `Option`

      Returns:
      :   true if the named `Option` is a member of this `Options`

      Since:
      :   1.3


    - #### getOptionGroup

      ```
      public OptionGroup getOptionGroup(Option opt)
      ```

      Returns the OptionGroup the `opt` belongs to.

      Parameters:
      :   `opt` - the option whose OptionGroup is being queried.

      Returns:
      :   the OptionGroup if `opt` is part
          of an OptionGroup, otherwise return null


    - #### toString

      ```
      public String toString()
      ```

      Dump state, suitable for debugging.

      Overrides:
      :   `toString` in class `Object`

      Returns:
      :   Stringified form of this object


Skip navigation links


- Package
- Class
- Use
- Tree
- Deprecated
- Index
- Help

- Prev Class
- Next Class

- Frames
- No Frames

- All Classes

- Summary:
- Nested |
- Field |
- Constr |
- Method

- Detail:
- Field |
- Constr |
- Method

Copyright © 2002–2015 The Apache Software Foundation. All rights reserved.
