# Supplementary material for: ACBM: An Integrated Agent and Constraint Based Modeling Framework for Simulation of Microbial Communities
Source: Sci Rep. 2020 May 26;10:8695. doi: 10.1038/s41598-020-65659-w (PMC7250870; doi:10.1038/s41598-020-65659-w)
Supplement: Supplementary file 2 [file 41598_2020_65659_MOESM2_ESM.zip › ACBM1.4/lib/commons-cli-1.3/apidocs/org/apache/commons/cli/package-summary.html]

org.apache.commons.cli (Apache Commons CLI 1.3 API)


JavaScript is disabled on your browser.


Skip navigation links


- Package
- Class
- Use
- Tree
- Deprecated
- Index
- Help

- Prev Package
- Next Package

- Frames
- No Frames

- All Classes

# Package org.apache.commons.cli

Commons CLI 1.3

See: Description

- Interface Summary

  | Interface | Description |
  |  |  |
  | --- | --- |
  | CommandLineParser | A class that implements the `CommandLineParser` interface can parse a String array according to the `Options` specified and return a `CommandLine`. |
- Class Summary

  | Class | Description |
  |  |  |
  | --- | --- |
  | BasicParser | Deprecated since 1.3, use the `DefaultParser` instead |
  | CommandLine | Represents list of arguments parsed against a `Options` descriptor. |
  | DefaultParser | Default parser. |
  | GnuParser | Deprecated since 1.3, use the `DefaultParser` instead |
  | HelpFormatter | A formatter of help messages for command line options. |
  | Option | Describes a single command-line option. |
  | Option.Builder | A nested builder class to create `Option` instances using descriptive methods. |
  | OptionBuilder | Deprecated since 1.3, use `Option.builder(String)` instead |
  | OptionGroup | A group of mutually exclusive options. |
  | Options | Main entry-point into the library. |
  | Parser | Deprecated since 1.3, the two-pass parsing with the flatten method is not enough flexible to handle complex cases |
  | PatternOptionBuilder | Allows Options to be created from a single String. |
  | PosixParser | Deprecated since 1.3, use the `DefaultParser` instead |
  | TypeHandler | This is a temporary implementation. |
- Exception Summary

  | Exception | Description |
  |  |  |
  | --- | --- |
  | AlreadySelectedException | Thrown when more than one option in an option group has been provided. |
  | AmbiguousOptionException | Exception thrown when an option can't be identified from a partial name. |
  | MissingArgumentException | Thrown when an option requiring an argument is not provided with an argument. |
  | MissingOptionException | Thrown when a required option has not been provided. |
  | ParseException | Base for Exceptions thrown during parsing of a command-line. |
  | UnrecognizedOptionException | Exception thrown during parsing signalling an unrecognized option was seen. |


## Package org.apache.commons.cli Description

Commons CLI 1.3

Version:
:   $Id: package-info.java 1443102 2013-02-06 18:12:16Z tn $

Skip navigation links


- Package
- Class
- Use
- Tree
- Deprecated
- Index
- Help

- Prev Package
- Next Package

- Frames
- No Frames

- All Classes

Copyright © 2002–2015 The Apache Software Foundation. All rights reserved.
