# Supplementary material for: ACBM: An Integrated Agent and Constraint Based Modeling Framework for Simulation of Microbial Communities
Source: Sci Rep. 2020 May 26;10:8695. doi: 10.1038/s41598-020-65659-w (PMC7250870; doi:10.1038/s41598-020-65659-w)
Supplement: Supplementary file 2 [file 41598_2020_65659_MOESM2_ESM.zip › ACBM1.4/lib/commons-cli-1.3/apidocs/org/apache/commons/cli/package-use.html]

Uses of Package org.apache.commons.cli (Apache Commons CLI 1.3 API)


JavaScript is disabled on your browser.


Skip navigation links


- Package
- Class
- Use
- Tree
- Deprecated
- Index
- Help

- Prev
- Next

- Frames
- No Frames

- All Classes

# Uses of Package org.apache.commons.cli

- Classes in org.apache.commons.cli used by org.apache.commons.cli

  | Class and Description |
  |  |
  | --- |
  | AlreadySelectedException Thrown when more than one option in an option group has been provided. |
  | CommandLine Represents list of arguments parsed against a `Options` descriptor. |
  | CommandLineParser A class that implements the `CommandLineParser` interface can parse a String array according to the `Options` specified and return a `CommandLine`. |
  | MissingOptionException Thrown when a required option has not been provided. |
  | Option Describes a single command-line option. |
  | Option.Builder A nested builder class to create `Option` instances using descriptive methods. |
  | OptionBuilder Deprecated. since 1.3, use `Option.builder(String)` instead |
  | OptionGroup A group of mutually exclusive options. |
  | Options Main entry-point into the library. |
  | ParseException Base for Exceptions thrown during parsing of a command-line. |
  | Parser Deprecated. since 1.3, the two-pass parsing with the flatten method is not enough flexible to handle complex cases |
  | UnrecognizedOptionException Exception thrown during parsing signalling an unrecognized option was seen. |

Skip navigation links


- Package
- Class
- Use
- Tree
- Deprecated
- Index
- Help

- Prev
- Next

- Frames
- No Frames

- All Classes

Copyright © 2002–2015 The Apache Software Foundation. All rights reserved.
