# Supplementary material for: ACBM: An Integrated Agent and Constraint Based Modeling Framework for Simulation of Microbial Communities
Source: Sci Rep. 2020 May 26;10:8695. doi: 10.1038/s41598-020-65659-w (PMC7250870; doi:10.1038/s41598-020-65659-w)
Supplement: Supplementary file 2 [file 41598_2020_65659_MOESM2_ESM.zip › ACBM1.4/lib/commons-cli-1.3/apidocs/org/apache/commons/cli/Parser.html]

Parser (Apache Commons CLI 1.3 API)


JavaScript is disabled on your browser.


Skip navigation links


- Package
- Class
- Use
- Tree
- Deprecated
- Index
- Help

- Prev Class
- Next Class

- Frames
- No Frames

- All Classes

- Summary:
- Nested |
- Field |
- Constr |
- Method

- Detail:
- Field |
- Constr |
- Method


org.apache.commons.cli

## Class Parser

- java.lang.Object
- - org.apache.commons.cli.Parser

- All Implemented Interfaces:
  :   CommandLineParser

  Direct Known Subclasses:
  :   BasicParser, GnuParser, PosixParser

  ---

  Deprecated.

  since 1.3, the two-pass parsing with the flatten method is not enough flexible to handle complex cases

    

  ```
  @Deprecated
  public abstract class Parser
  extends Object
  implements CommandLineParser
  ```

  `Parser` creates `CommandLine`s.

  Version:
  :   $Id: Parser.java 1677406 2015-05-03 14:27:31Z britter $

- - ### Field Summary

    Fields

    | Modifier and Type | Field and Description |
    | `protected CommandLine` | `cmd` Deprecated.  commandline instance |
  - ### Constructor Summary

    Constructors

    | Constructor and Description |
    | `Parser()` Deprecated. |
  - ### Method Summary

    All Methods Instance Methods Abstract Methods Concrete Methods Deprecated Methods

    | Modifier and Type | Method and Description |
    | `protected void` | `checkRequiredOptions()` Deprecated.  Throws a `MissingOptionException` if all of the required options are not present. |
    | `protected abstract String[]` | `flatten(Options opts, String[] arguments, boolean stopAtNonOption)` Deprecated.  Subclasses must implement this method to reduce the `arguments` that have been passed to the parse method. |
    | `protected Options` | `getOptions()` Deprecated. |
    | `protected List` | `getRequiredOptions()` Deprecated. |
    | `CommandLine` | `parse(Options options, String[] arguments)` Deprecated.  Parses the specified `arguments` based on the specified `Options`. |
    | `CommandLine` | `parse(Options options, String[] arguments, boolean stopAtNonOption)` Deprecated.  Parses the specified `arguments` based on the specified `Options`. |
    | `CommandLine` | `parse(Options options, String[] arguments, Properties properties)` Deprecated.  Parse the arguments according to the specified options and properties. |
    | `CommandLine` | `parse(Options options, String[] arguments, Properties properties, boolean stopAtNonOption)` Deprecated.  Parse the arguments according to the specified options and properties. |
    | `void` | `processArgs(Option opt, ListIterator<String> iter)` Deprecated.  Process the argument values for the specified Option `opt` using the values retrieved from the specified iterator `iter`. |
    | `protected void` | `processOption(String arg, ListIterator<String> iter)` Deprecated.  Process the Option specified by `arg` using the values retrieved from the specified iterator `iter`. |
    | `protected void` | `processProperties(Properties properties)` Deprecated.  Sets the values of Options using the values in `properties`. |
    | `protected void` | `setOptions(Options options)` Deprecated. |

    - ### Methods inherited from class java.lang.Object

      `clone, equals, finalize, getClass, hashCode, notify, notifyAll, toString, wait, wait, wait`

- - ### Field Detail


    - #### cmd

      ```
      protected CommandLine cmd
      ```

      Deprecated.

      commandline instance
  - ### Constructor Detail


    - #### Parser

      ```
      public Parser()
      ```

      Deprecated.
  - ### Method Detail


    - #### setOptions

      ```
      protected void setOptions(Options options)
      ```

      Deprecated.


    - #### getOptions

      ```
      protected Options getOptions()
      ```

      Deprecated.


    - #### getRequiredOptions

      ```
      protected List getRequiredOptions()
      ```

      Deprecated.


    - #### flatten

      ```
      protected abstract String[] flatten(Options opts,
                                          String[] arguments,
                                          boolean stopAtNonOption)
                                   throws ParseException
      ```

      Deprecated.

      Subclasses must implement this method to reduce
      the `arguments` that have been passed to the parse method.

      Parameters:
      :   `opts` - The Options to parse the arguments by.
      :   `arguments` - The arguments that have to be flattened.
      :   `stopAtNonOption` - specifies whether to stop
          flattening when a non option has been encountered

      Returns:
      :   a String array of the flattened arguments

      Throws:
      :   `ParseException` - if there are any problems encountered
          while parsing the command line tokens.


    - #### parse

      ```
      public CommandLine parse(Options options,
                               String[] arguments)
                        throws ParseException
      ```

      Deprecated.

      Parses the specified `arguments` based
      on the specified `Options`.

      Specified by:
      :   `parse` in interface `CommandLineParser`

      Parameters:
      :   `options` - the `Options`
      :   `arguments` - the `arguments`

      Returns:
      :   the `CommandLine`

      Throws:
      :   `ParseException` - if there are any problems encountered
          while parsing the command line tokens.


    - #### parse

      ```
      public CommandLine parse(Options options,
                               String[] arguments,
                               Properties properties)
                        throws ParseException
      ```

      Deprecated.

      Parse the arguments according to the specified options and properties.

      Parameters:
      :   `options` - the specified Options
      :   `arguments` - the command line arguments
      :   `properties` - command line option name-value pairs

      Returns:
      :   the list of atomic option and value tokens

      Throws:
      :   `ParseException` - if there are any problems encountered
          while parsing the command line tokens.

      Since:
      :   1.1


    - #### parse

      ```
      public CommandLine parse(Options options,
                               String[] arguments,
                               boolean stopAtNonOption)
                        throws ParseException
      ```

      Deprecated.

      Parses the specified `arguments`
      based on the specified `Options`.

      Specified by:
      :   `parse` in interface `CommandLineParser`

      Parameters:
      :   `options` - the `Options`
      :   `arguments` - the `arguments`
      :   `stopAtNonOption` - if true an unrecognized argument stops
          the parsing and the remaining arguments are added to the
          `CommandLine`s args list. If false an unrecognized
          argument triggers a ParseException.

      Returns:
      :   the `CommandLine`

      Throws:
      :   `ParseException` - if an error occurs when parsing the arguments.


    - #### parse

      ```
      public CommandLine parse(Options options,
                               String[] arguments,
                               Properties properties,
                               boolean stopAtNonOption)
                        throws ParseException
      ```

      Deprecated.

      Parse the arguments according to the specified options and
      properties.

      Parameters:
      :   `options` - the specified Options
      :   `arguments` - the command line arguments
      :   `properties` - command line option name-value pairs
      :   `stopAtNonOption` - if true an unrecognized argument stops
          the parsing and the remaining arguments are added to the
          `CommandLine`s args list. If false an unrecognized
          argument triggers a ParseException.

      Returns:
      :   the list of atomic option and value tokens

      Throws:
      :   `ParseException` - if there are any problems encountered
          while parsing the command line tokens.

      Since:
      :   1.1


    - #### processProperties

      ```
      protected void processProperties(Properties properties)
                                throws ParseException
      ```

      Deprecated.

      Sets the values of Options using the values in `properties`.

      Parameters:
      :   `properties` - The value properties to be processed.

      Throws:
      :   `ParseException` - if there are any problems encountered
          while processing the properties.


    - #### checkRequiredOptions

      ```
      protected void checkRequiredOptions()
                                   throws MissingOptionException
      ```

      Deprecated.

      Throws a `MissingOptionException` if all of the required options
      are not present.

      Throws:
      :   `MissingOptionException` - if any of the required Options are not present.


    - #### processArgs

      ```
      public void processArgs(Option opt,
                              ListIterator<String> iter)
                       throws ParseException
      ```

      Deprecated.

      Process the argument values for the specified Option
      `opt` using the values retrieved from the
      specified iterator `iter`.

      Parameters:
      :   `opt` - The current Option
      :   `iter` - The iterator over the flattened command line Options.

      Throws:
      :   `ParseException` - if an argument value is required
          and it is has not been found.


    - #### processOption

      ```
      protected void processOption(String arg,
                                   ListIterator<String> iter)
                            throws ParseException
      ```

      Deprecated.

      Process the Option specified by `arg` using the values
      retrieved from the specified iterator `iter`.

      Parameters:
      :   `arg` - The String value representing an Option
      :   `iter` - The iterator over the flattened command line arguments.

      Throws:
      :   `ParseException` - if `arg` does not represent an Option


Skip navigation links


- Package
- Class
- Use
- Tree
- Deprecated
- Index
- Help

- Prev Class
- Next Class

- Frames
- No Frames

- All Classes

- Summary:
- Nested |
- Field |
- Constr |
- Method

- Detail:
- Field |
- Constr |
- Method

Copyright © 2002–2015 The Apache Software Foundation. All rights reserved.
