# Supplementary material for: ACBM: An Integrated Agent and Constraint Based Modeling Framework for Simulation of Microbial Communities
Source: Sci Rep. 2020 May 26;10:8695. doi: 10.1038/s41598-020-65659-w (PMC7250870; doi:10.1038/s41598-020-65659-w)
Supplement: Supplementary file 2 [file 41598_2020_65659_MOESM2_ESM.zip › ACBM1.4/lib/commons-cli-1.3/apidocs/org/apache/commons/cli/PatternOptionBuilder.html]

PatternOptionBuilder (Apache Commons CLI 1.3 API)


JavaScript is disabled on your browser.


Skip navigation links


- Package
- Class
- Use
- Tree
- Deprecated
- Index
- Help

- Prev Class
- Next Class

- Frames
- No Frames

- All Classes

- Summary:
- Nested |
- Field |
- Constr |
- Method

- Detail:
- Field |
- Constr |
- Method


org.apache.commons.cli

## Class PatternOptionBuilder

- java.lang.Object
- - org.apache.commons.cli.PatternOptionBuilder

- ---

    

  ```
  public class PatternOptionBuilder
  extends Object
  ```

  Allows Options to be created from a single String.
  The pattern contains various single character flags and via
  an optional punctuation character, their expected type.

  Overview of PatternOptionBuilder patterns

  | a | -a flag |
  | b@ | -b [classname] |
  | c> | -c [filename] |
  | d+ | -d [classname] (creates object via empty constructor) |
  | e% | -e [number] (creates Double/Long instance depending on existing of a '.') |
  | f/ | -f [url] |
  | g: | -g [string] |

  For example, the following allows command line flags of '-v -p string-value -f /dir/file'.
  The exclamation mark precede a mandatory option.

  ```
       Options options = PatternOptionBuilder.parsePattern("vp:!f/");
  ```

  TODO: These need to break out to OptionType and also to be pluggable.

  Version:
  :   $Id: PatternOptionBuilder.java 1677406 2015-05-03 14:27:31Z britter $

- - ### Field Summary

    Fields

    | Modifier and Type | Field and Description |
    | `static Class<?>` | `CLASS_VALUE` Class class |
    | `static Class<Date>` | `DATE_VALUE` Date class |
    | `static Class<FileInputStream>` | `EXISTING_FILE_VALUE` FileInputStream class |
    | `static Class<File>` | `FILE_VALUE` File class |
    | `static Class<File[]>` | `FILES_VALUE` File array class |
    | `static Class<Number>` | `NUMBER_VALUE` Number class |
    | `static Class<Object>` | `OBJECT_VALUE` Object class |
    | `static Class<String>` | `STRING_VALUE` String class |
    | `static Class<URL>` | `URL_VALUE` URL class |
  - ### Constructor Summary

    Constructors

    | Constructor and Description |
    | `PatternOptionBuilder()` |
  - ### Method Summary

    All Methods Static Methods Concrete Methods

    | Modifier and Type | Method and Description |
    | `static Object` | `getValueClass(char ch)` Retrieve the class that `ch` represents. |
    | `static boolean` | `isValueCode(char ch)` Returns whether `ch` is a value code, i.e. |
    | `static Options` | `parsePattern(String pattern)` Returns the `Options` instance represented by `pattern`. |

    - ### Methods inherited from class java.lang.Object

      `clone, equals, finalize, getClass, hashCode, notify, notifyAll, toString, wait, wait, wait`

- - ### Field Detail


    - #### STRING\_VALUE

      ```
      public static final Class<String> STRING_VALUE
      ```

      String class


    - #### OBJECT\_VALUE

      ```
      public static final Class<Object> OBJECT_VALUE
      ```

      Object class


    - #### NUMBER\_VALUE

      ```
      public static final Class<Number> NUMBER_VALUE
      ```

      Number class


    - #### DATE\_VALUE

      ```
      public static final Class<Date> DATE_VALUE
      ```

      Date class


    - #### CLASS\_VALUE

      ```
      public static final Class<?> CLASS_VALUE
      ```

      Class class


    - #### EXISTING\_FILE\_VALUE

      ```
      public static final Class<FileInputStream> EXISTING_FILE_VALUE
      ```

      FileInputStream class


    - #### FILE\_VALUE

      ```
      public static final Class<File> FILE_VALUE
      ```

      File class


    - #### FILES\_VALUE

      ```
      public static final Class<File[]> FILES_VALUE
      ```

      File array class


    - #### URL\_VALUE

      ```
      public static final Class<URL> URL_VALUE
      ```

      URL class
  - ### Constructor Detail


    - #### PatternOptionBuilder

      ```
      public PatternOptionBuilder()
      ```
  - ### Method Detail


    - #### getValueClass

      ```
      public static Object getValueClass(char ch)
      ```

      Retrieve the class that `ch` represents.

      Parameters:
      :   `ch` - the specified character

      Returns:
      :   The class that `ch` represents


    - #### isValueCode

      ```
      public static boolean isValueCode(char ch)
      ```

      Returns whether `ch` is a value code, i.e.
      whether it represents a class in a pattern.

      Parameters:
      :   `ch` - the specified character

      Returns:
      :   true if `ch` is a value code, otherwise false.


    - #### parsePattern

      ```
      public static Options parsePattern(String pattern)
      ```

      Returns the `Options` instance represented by `pattern`.

      Parameters:
      :   `pattern` - the pattern string

      Returns:
      :   The `Options` instance


Skip navigation links


- Package
- Class
- Use
- Tree
- Deprecated
- Index
- Help

- Prev Class
- Next Class

- Frames
- No Frames

- All Classes

- Summary:
- Nested |
- Field |
- Constr |
- Method

- Detail:
- Field |
- Constr |
- Method

Copyright © 2002–2015 The Apache Software Foundation. All rights reserved.
