# Supplementary material for: ACBM: An Integrated Agent and Constraint Based Modeling Framework for Simulation of Microbial Communities
Source: Sci Rep. 2020 May 26;10:8695. doi: 10.1038/s41598-020-65659-w (PMC7250870; doi:10.1038/s41598-020-65659-w)
Supplement: Supplementary file 2 [file 41598_2020_65659_MOESM2_ESM.zip › ACBM1.4/lib/commons-cli-1.3/apidocs/org/apache/commons/cli/PosixParser.html]

PosixParser (Apache Commons CLI 1.3 API)


JavaScript is disabled on your browser.


Skip navigation links


- Package
- Class
- Use
- Tree
- Deprecated
- Index
- Help

- Prev Class
- Next Class

- Frames
- No Frames

- All Classes

- Summary:
- Nested |
- Field |
- Constr |
- Method

- Detail:
- Field |
- Constr |
- Method


org.apache.commons.cli

## Class PosixParser

- java.lang.Object
- - org.apache.commons.cli.Parser
  - - org.apache.commons.cli.PosixParser

- All Implemented Interfaces:
  :   CommandLineParser

  ---

  Deprecated.

  since 1.3, use the `DefaultParser` instead

    

  ```
  @Deprecated
  public class PosixParser
  extends Parser
  ```

  The class PosixParser provides an implementation of the
  `flatten` method.

  Version:
  :   $Id: PosixParser.java 1677406 2015-05-03 14:27:31Z britter $

- - ### Field Summary

    - ### Fields inherited from class org.apache.commons.cli.Parser

      `cmd`
  - ### Constructor Summary

    Constructors

    | Constructor and Description |
    | `PosixParser()` Deprecated. |
  - ### Method Summary

    All Methods Instance Methods Concrete Methods Deprecated Methods

    | Modifier and Type | Method and Description |
    | `protected void` | `burstToken(String token, boolean stopAtNonOption)` Deprecated.  Breaks `token` into its constituent parts using the following algorithm. |
    | `protected String[]` | `flatten(Options options, String[] arguments, boolean stopAtNonOption)` Deprecated.  An implementation of `Parser`'s abstract `flatten` method. |

    - ### Methods inherited from class org.apache.commons.cli.Parser

      `checkRequiredOptions, getOptions, getRequiredOptions, parse, parse, parse, parse, processArgs, processOption, processProperties, setOptions`
    - ### Methods inherited from class java.lang.Object

      `clone, equals, finalize, getClass, hashCode, notify, notifyAll, toString, wait, wait, wait`

- - ### Constructor Detail


    - #### PosixParser

      ```
      public PosixParser()
      ```

      Deprecated.
  - ### Method Detail


    - #### flatten

      ```
      protected String[] flatten(Options options,
                                 String[] arguments,
                                 boolean stopAtNonOption)
                          throws ParseException
      ```

      Deprecated.

      An implementation of `Parser`'s abstract
      `flatten` method.

      The following are the rules used by this flatten method.

      1. if `stopAtNonOption` is **true** then do not
         burst anymore of `arguments` entries, just add each
         successive entry without further processing. Otherwise, ignore
         `stopAtNonOption`.
      2. if the current `arguments` entry is "**--**"
         just add the entry to the list of processed tokens
      3. if the current `arguments` entry is "**-**"
         just add the entry to the list of processed tokens
      4. if the current `arguments` entry is two characters
         in length and the first character is "**-**" then check if this
         is a valid `Option` id. If it is a valid id, then add the
         entry to the list of processed tokens and set the current `Option`
         member. If it is not a valid id and `stopAtNonOption`
         is true, then the remaining entries are copied to the list of
         processed tokens. Otherwise, the current entry is ignored.
      5. if the current `arguments` entry is more than two
         characters in length and the first character is "**-**" then
         we need to burst the entry to determine its constituents. For more
         information on the bursting algorithm see
         `burstToken`.
      6. if the current `arguments` entry is not handled
         by any of the previous rules, then the entry is added to the list
         of processed tokens.

      Specified by:
      :   `flatten` in class `Parser`

      Parameters:
      :   `options` - The command line `Options`
      :   `arguments` - The command line arguments to be parsed
      :   `stopAtNonOption` - Specifies whether to stop flattening
          when an non option is found.

      Returns:
      :   The flattened `arguments` String array.

      Throws:
      :   `ParseException` - if there are any problems encountered
          while parsing the command line tokens.


    - #### burstToken

      ```
      protected void burstToken(String token,
                                boolean stopAtNonOption)
      ```

      Deprecated.

      Breaks `token` into its constituent parts
      using the following algorithm.
      - ignore the first character ("**-**")
      - foreach remaining character check if an `Option`
        exists with that id.
      - if an `Option` does exist then add that character
        prepended with "**-**" to the list of processed tokens.
      - if the `Option` can have an argument value and there
        are remaining characters in the token then add the remaining
        characters as a token to the list of processed tokens.
      - if an `Option` does **NOT** exist **AND**
        `stopAtNonOption` **IS** set then add the special token
        "**--**" followed by the remaining characters and also
        the remaining tokens directly to the processed tokens list.
      - if an `Option` does **NOT** exist **AND**
        `stopAtNonOption` **IS NOT** set then add that
        character prepended with "**-**".

      Parameters:
      :   `token` - The current token to be **burst**
      :   `stopAtNonOption` - Specifies whether to stop processing
          at the first non-Option encountered.


Skip navigation links


- Package
- Class
- Use
- Tree
- Deprecated
- Index
- Help

- Prev Class
- Next Class

- Frames
- No Frames

- All Classes

- Summary:
- Nested |
- Field |
- Constr |
- Method

- Detail:
- Field |
- Constr |
- Method

Copyright © 2002–2015 The Apache Software Foundation. All rights reserved.
