# Supplementary material for: ACBM: An Integrated Agent and Constraint Based Modeling Framework for Simulation of Microbial Communities
Source: Sci Rep. 2020 May 26;10:8695. doi: 10.1038/s41598-020-65659-w (PMC7250870; doi:10.1038/s41598-020-65659-w)
Supplement: Supplementary file 2 [file 41598_2020_65659_MOESM2_ESM.zip › ACBM1.4/lib/commons-cli-1.3/apidocs/org/apache/commons/cli/TypeHandler.html]

TypeHandler (Apache Commons CLI 1.3 API)


JavaScript is disabled on your browser.


Skip navigation links


- Package
- Class
- Use
- Tree
- Deprecated
- Index
- Help

- Prev Class
- Next Class

- Frames
- No Frames

- All Classes

- Summary:
- Nested |
- Field |
- Constr |
- Method

- Detail:
- Field |
- Constr |
- Method


org.apache.commons.cli

## Class TypeHandler

- java.lang.Object
- - org.apache.commons.cli.TypeHandler

- ---

    

  ```
  public class TypeHandler
  extends Object
  ```

  This is a temporary implementation. TypeHandler will handle the
  pluggableness of OptionTypes and it will direct all of these types
  of conversion functionalities to ConvertUtils component in Commons
  already. BeanUtils I think.

  Version:
  :   $Id: TypeHandler.java 1443102 2013-02-06 18:12:16Z tn $

- - ### Constructor Summary

    Constructors

    | Constructor and Description |
    | `TypeHandler()` |
  - ### Method Summary

    All Methods Static Methods Concrete Methods

    | Modifier and Type | Method and Description |
    | `static Class<?>` | `createClass(String classname)` Returns the class whose name is `classname`. |
    | `static Date` | `createDate(String str)` Returns the date represented by `str`. |
    | `static File` | `createFile(String str)` Returns the File represented by `str`. |
    | `static File[]` | `createFiles(String str)` Returns the File[] represented by `str`. |
    | `static Number` | `createNumber(String str)` Create a number from a String. |
    | `static Object` | `createObject(String classname)` Create an Object from the classname and empty constructor. |
    | `static URL` | `createURL(String str)` Returns the URL represented by `str`. |
    | `static Object` | `createValue(String str, Class<?> clazz)` Returns the `Object` of type `clazz` with the value of `str`. |
    | `static Object` | `createValue(String str, Object obj)` Returns the `Object` of type `obj` with the value of `str`. |

    - ### Methods inherited from class java.lang.Object

      `clone, equals, finalize, getClass, hashCode, notify, notifyAll, toString, wait, wait, wait`

- - ### Constructor Detail


    - #### TypeHandler

      ```
      public TypeHandler()
      ```
  - ### Method Detail


    - #### createValue

      ```
      public static Object createValue(String str,
                                       Object obj)
                                throws ParseException
      ```

      Returns the `Object` of type `obj`
      with the value of `str`.

      Parameters:
      :   `str` - the command line value
      :   `obj` - the type of argument

      Returns:
      :   The instance of `obj` initialised with
          the value of `str`.

      Throws:
      :   `ParseException` - if the value creation for the given object type failed


    - #### createValue

      ```
      public static Object createValue(String str,
                                       Class<?> clazz)
                                throws ParseException
      ```

      Returns the `Object` of type `clazz`
      with the value of `str`.

      Parameters:
      :   `str` - the command line value
      :   `clazz` - the type of argument

      Returns:
      :   The instance of `clazz` initialised with
          the value of `str`.

      Throws:
      :   `ParseException` - if the value creation for the given class failed


    - #### createObject

      ```
      public static Object createObject(String classname)
                                 throws ParseException
      ```

      Create an Object from the classname and empty constructor.

      Parameters:
      :   `classname` - the argument value

      Returns:
      :   the initialised object

      Throws:
      :   `ParseException` - if the class could not be found or the object could not be created


    - #### createNumber

      ```
      public static Number createNumber(String str)
                                 throws ParseException
      ```

      Create a number from a String. If a . is present, it creates a
      Double, otherwise a Long.

      Parameters:
      :   `str` - the value

      Returns:
      :   the number represented by `str`

      Throws:
      :   `ParseException` - if `str` is not a number


    - #### createClass

      ```
      public static Class<?> createClass(String classname)
                                  throws ParseException
      ```

      Returns the class whose name is `classname`.

      Parameters:
      :   `classname` - the class name

      Returns:
      :   The class if it is found

      Throws:
      :   `ParseException` - if the class could not be found


    - #### createDate

      ```
      public static Date createDate(String str)
      ```

      Returns the date represented by `str`.

      This method is not yet implemented and always throws an
      `UnsupportedOperationException`.

      Parameters:
      :   `str` - the date string

      Returns:
      :   The date if `str` is a valid date string,
          otherwise return null.

      Throws:
      :   `UnsupportedOperationException` - always


    - #### createURL

      ```
      public static URL createURL(String str)
                           throws ParseException
      ```

      Returns the URL represented by `str`.

      Parameters:
      :   `str` - the URL string

      Returns:
      :   The URL in `str` is well-formed

      Throws:
      :   `ParseException` - if the URL in `str` is not well-formed


    - #### createFile

      ```
      public static File createFile(String str)
      ```

      Returns the File represented by `str`.

      Parameters:
      :   `str` - the File location

      Returns:
      :   The file represented by `str`.


    - #### createFiles

      ```
      public static File[] createFiles(String str)
      ```

      Returns the File[] represented by `str`.

      This method is not yet implemented and always throws an
      `UnsupportedOperationException`.

      Parameters:
      :   `str` - the paths to the files

      Returns:
      :   The File[] represented by `str`.

      Throws:
      :   `UnsupportedOperationException` - always


Skip navigation links


- Package
- Class
- Use
- Tree
- Deprecated
- Index
- Help

- Prev Class
- Next Class

- Frames
- No Frames

- All Classes

- Summary:
- Nested |
- Field |
- Constr |
- Method

- Detail:
- Field |
- Constr |
- Method

Copyright © 2002–2015 The Apache Software Foundation. All rights reserved.
