# Supplementary material for: ACBM: An Integrated Agent and Constraint Based Modeling Framework for Simulation of Microbial Communities
Source: Sci Rep. 2020 May 26;10:8695. doi: 10.1038/s41598-020-65659-w (PMC7250870; doi:10.1038/s41598-020-65659-w)
Supplement: Supplementary file 2 [file 41598_2020_65659_MOESM2_ESM.zip › ACBM1.4/lib/commons-cli-1.3/apidocs/overview-tree.html]

Class Hierarchy (Apache Commons CLI 1.3 API)


JavaScript is disabled on your browser.


Skip navigation links


- Package
- Class
- Use
- Tree
- Deprecated
- Index
- Help

- Prev
- Next

- Frames
- No Frames

- All Classes

# Hierarchy For All Packages

Package Hierarchies:

- org.apache.commons.cli

## Class Hierarchy

- java.lang.Object
  - org.apache.commons.cli.CommandLine (implements java.io.Serializable)
  - org.apache.commons.cli.DefaultParser (implements org.apache.commons.cli.CommandLineParser)
  - org.apache.commons.cli.HelpFormatter
  - org.apache.commons.cli.Option (implements java.lang.Cloneable, java.io.Serializable)
  - org.apache.commons.cli.Option.Builder
  - org.apache.commons.cli.OptionBuilder
  - org.apache.commons.cli.OptionGroup (implements java.io.Serializable)
  - org.apache.commons.cli.Options (implements java.io.Serializable)
  - org.apache.commons.cli.Parser (implements org.apache.commons.cli.CommandLineParser)
    - org.apache.commons.cli.BasicParser
    - org.apache.commons.cli.GnuParser
    - org.apache.commons.cli.PosixParser
  - org.apache.commons.cli.PatternOptionBuilder
  - java.lang.Throwable (implements java.io.Serializable)
    - java.lang.Exception
      - org.apache.commons.cli.ParseException
        - org.apache.commons.cli.AlreadySelectedException
        - org.apache.commons.cli.MissingArgumentException
        - org.apache.commons.cli.MissingOptionException
        - org.apache.commons.cli.UnrecognizedOptionException
          - org.apache.commons.cli.AmbiguousOptionException
  - org.apache.commons.cli.TypeHandler

## Interface Hierarchy

- org.apache.commons.cli.CommandLineParser

Skip navigation links


- Package
- Class
- Use
- Tree
- Deprecated
- Index
- Help

- Prev
- Next

- Frames
- No Frames

- All Classes

Copyright © 2002–2015 The Apache Software Foundation. All rights reserved.
