# Supplementary material for: ACBM: An Integrated Agent and Constraint Based Modeling Framework for Simulation of Microbial Communities
Source: Sci Rep. 2020 May 26;10:8695. doi: 10.1038/s41598-020-65659-w (PMC7250870; doi:10.1038/s41598-020-65659-w)
Supplement: Supplementary file 2 [file 41598_2020_65659_MOESM2_ESM.zip › ACBM1.4/lib/commons-cli-1.3/apidocs/serialized-form.html]

Serialized Form (Apache Commons CLI 1.3 API)


JavaScript is disabled on your browser.


Skip navigation links


- Package
- Class
- Use
- Tree
- Deprecated
- Index
- Help

- Prev
- Next

- Frames
- No Frames

- All Classes

# Serialized Form

- ## Package org.apache.commons.cli

  - ### Class org.apache.commons.cli.AlreadySelectedException extends ParseException implements Serializable

    serialVersionUID:
    :   3674381532418544760L

    - ### Serialized Fields

      - #### group

        ```
        OptionGroup group
        ```

        The option group selected.
      - #### option

        ```
        Option option
        ```

        The option that triggered the exception.
  - ### Class org.apache.commons.cli.AmbiguousOptionException extends UnrecognizedOptionException implements Serializable

    serialVersionUID:
    :   5829816121277947229L

    - ### Serialized Fields

      - #### matchingOptions

        ```
        Collection<E> matchingOptions
        ```

        The list of options matching the partial name specified
  - ### Class org.apache.commons.cli.CommandLine extends Object implements Serializable

    serialVersionUID:
    :   1L

    - ### Serialized Fields

      - #### args

        ```
        List<E> args
        ```

        the unrecognised options/arguments
      - #### options

        ```
        List<E> options
        ```

        the processed options
  - ### Class org.apache.commons.cli.MissingArgumentException extends ParseException implements Serializable

    serialVersionUID:
    :   -7098538588704965017L

    - ### Serialized Fields

      - #### option

        ```
        Option option
        ```

        The option requiring additional arguments
  - ### Class org.apache.commons.cli.MissingOptionException extends ParseException implements Serializable

    serialVersionUID:
    :   8161889051578563249L

    - ### Serialized Fields

      - #### missingOptions

        ```
        List<E> missingOptions
        ```

        The list of missing options and groups
  - ### Class org.apache.commons.cli.Option extends Object implements Serializable

    serialVersionUID:
    :   1L

    - ### Serialized Fields

      - #### opt

        ```
        String opt
        ```

        the name of the option
      - #### longOpt

        ```
        String longOpt
        ```

        the long representation of the option
      - #### argName

        ```
        String argName
        ```

        the name of the argument for this option
      - #### description

        ```
        String description
        ```

        description of the option
      - #### required

        ```
        boolean required
        ```

        specifies whether this option is required to be present
      - #### optionalArg

        ```
        boolean optionalArg
        ```

        specifies whether the argument value of this Option is optional
      - #### numberOfArgs

        ```
        int numberOfArgs
        ```

        the number of argument values this option can have
      - #### type

        ```
        Class<T> type
        ```

        the type of this Option
      - #### values

        ```
        List<E> values
        ```

        the list of argument values
      - #### valuesep

        ```
        char valuesep
        ```

        the character that is the value separator
  - ### Class org.apache.commons.cli.OptionGroup extends Object implements Serializable

    serialVersionUID:
    :   1L

    - ### Serialized Fields

      - #### optionMap

        ```
        Map<K,V> optionMap
        ```

        hold the options
      - #### selected

        ```
        String selected
        ```

        the name of the selected option
      - #### required

        ```
        boolean required
        ```

        specified whether this group is required
  - ### Class org.apache.commons.cli.Options extends Object implements Serializable

    serialVersionUID:
    :   1L

    - ### Serialized Fields

      - #### shortOpts

        ```
        Map<K,V> shortOpts
        ```

        a map of the options with the character key
      - #### longOpts

        ```
        Map<K,V> longOpts
        ```

        a map of the options with the long key
      - #### requiredOpts

        ```
        List<E> requiredOpts
        ```

        a map of the required options
      - #### optionGroups

        ```
        Map<K,V> optionGroups
        ```

        a map of the option groups
  - ### Class org.apache.commons.cli.ParseException extends Exception implements Serializable

    serialVersionUID:
    :   9112808380089253192L
  - ### Class org.apache.commons.cli.UnrecognizedOptionException extends ParseException implements Serializable

    serialVersionUID:
    :   -252504690284625623L

    - ### Serialized Fields

      - #### option

        ```
        String option
        ```

        The unrecognized option

Skip navigation links


- Package
- Class
- Use
- Tree
- Deprecated
- Index
- Help

- Prev
- Next

- Frames
- No Frames

- All Classes

Copyright © 2002–2015 The Apache Software Foundation. All rights reserved.
