# Supplementary material for: ACBM: An Integrated Agent and Constraint Based Modeling Framework for Simulation of Microbial Communities
Source: Sci Rep. 2020 May 26;10:8695. doi: 10.1038/s41598-020-65659-w (PMC7250870; doi:10.1038/s41598-020-65659-w)
Supplement: Supplementary file 3 [file 41598_2020_65659_MOESM3_ESM.pdf]

ACBM: An Integrated Agent and Constraint Based Modeling Framework for Simulation of Microbial Communities

Emadoddin Karimian and Ehsan Motamedian

Supplementary file 3

Table S1. The specific properties for some microorganisms.

**Name**	*E. coli*	*B. subtilis*	*S. cerevisiae*	*S. stipitis*	*B. adolescentis*	*F. prausnitzii*	*C. beijerinckii*
**Scale**	500	500	500	500	500	500	500
**R (μm)**	0.6	0.76	2.92	2.4	0.9[1]	0.65[2]	0.75
**L (μm)**	1[3]	1.25[4]	3.1[5]	2.4[5]	4.75[4]	5.5[2]	1.7
**Mass (pg)**	1.29[6]	2.5	93[5]	48.4[5]	13.3	8.03	3.4
**Eat radius (Bacterial length×)**	4	4	4	4	4	4	4
**Mat file Name**	iJO1366TRFBA	iYO844	iMM904	iBB814	iBif452	iFap484	iJL432
**Speed (μm/s)**	8000	8000	8000	8000	8000	8000	8000
**Search radius (μm)** [7]	3500	3500	3500	3500	3500	3500	3500
**Survival time (min)**	360	360	360	360	360	360	360

Table S2. Comparison of average reaction fluxes predicted by ACBM and measured specific activity of 22 enzymes of *E. coli* central metabolic pathways at exponential (6 h) and early stationary (8 h) phases of growth

**Pathway**	**Enzyme**	**Predicted fluxes by ACBM (mmol/gDCW/h)**		**The specific activity of enzymes measured by Rahman *et al.*[8] (μmol/mg protein/min)**	
		**Exponential phase**	**Early stationary phase**	**Exponential phase**	**Early stationary phase**
Glycolytic pathway	PFK	11.24	5.04	0.93	0.09
	PGI	19.49	10.44	2.36	1.59
	GAPDH	40.51	21.06	0.11	0.016
	Pyk	8.15	3.11	0.51	0.16
	Ppc	1.92	1	0.12	0.054
Pentose phosphate pathway	G-6PDH	0	0	0.33	0.104
	6-PGDH	0	0	0.28	0.12
TCA cycle	CS	5.49	3.22	0.02	0.25
	ACONTa	5.49	3.22	0.16	0.31
	ICDH	5.49	3.21	0.97	0.59
	SDH	4.99	2.95	0.09	0.14
	FUM	5.44	3.19	0.011	0.021
	MDH	0.9	0.4	0.006	0.319
Glyoxylate pathway	ICL	0	0.01	0.05	0.33
	MS	0.0006	0.01	0.043	0.29
Acetate metabolism	Ack	10.61	6.77	0.81	0.57
	Acs	0	0	0.06	0.1
Gluconeogenesis	Fbp	0	0	0.041	0.138
	Pck	0	0	0.015	0.15
	MEZ (NAD+-dependent)	0	0	0.02	0.026
	MEZ (NADP+-dependent)	0	0	0.018	0.075
Lactate dehydrogenase	LDH	9.42	4.83	0.04	0.002

Pgi: phosphoglucoisomerase, Pfk: phosphofructokinase, GAPDH: glyceraldehyde-3P dehydrogenase, Pyk: pyruvate kinase, Ppc: phosphoenolpyruvate carboxylase, G6PDH: glucose-6P dehydrogenase, 6PGDH: 6-phosphogluconate dehydrogenase, CS: citrate synthase, Aco: aconitase, ICDH: isocitrate dehydrogenase, SDH: succinate dehydrogenase, Fum: fumarase, MDH: malate dehydrogenase, Icl: isocitrate lyase, Ms: malate synthase, Ack: acetate kinase, Acs: acetyl-CoA synthetase, Fbp: fructose bis-phosphatase, Pck: phosphoenolpyruvate kinase, MEZ: NAD^+-dependent malic enzyme (encoded by sfcA), MEZ: $NADP^+$-dependent malic enzyme (encoded by maeB), LDH: lactate dehydrogenase

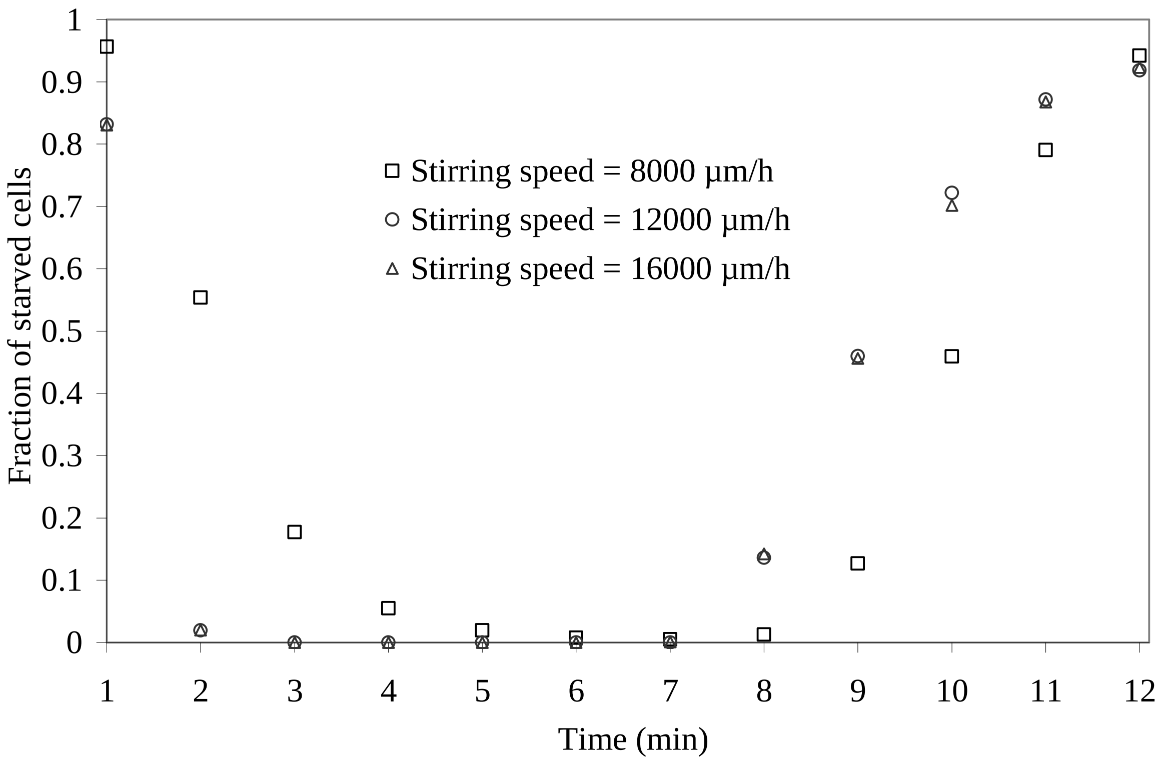

Figure S1. Effect of stirring speed on the percentage of starved cells over time under heterogeneous feeding of 10 g/l glucose in a microbioreactor with a volume of 0.16 µl including 60 g/l biomass of *E. coli* from coordinate x=500, y=0, and z=0.

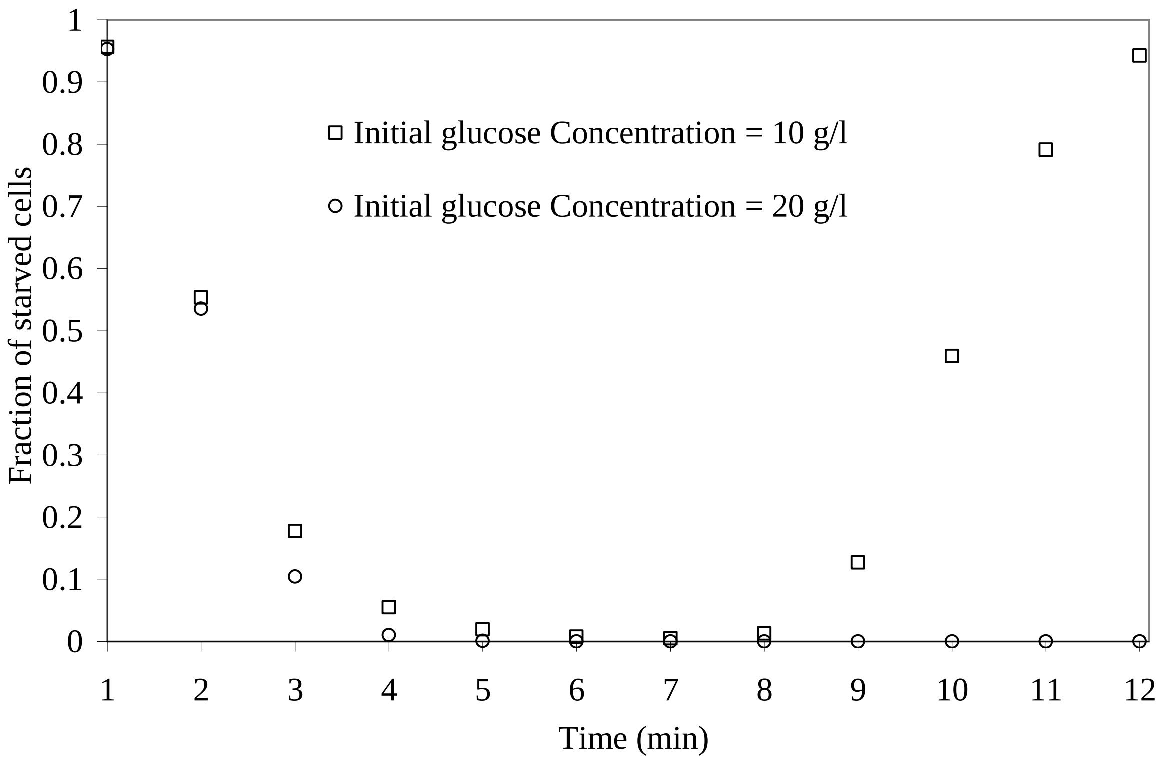

Figure S2. Effect of initial glucose concentration on the percentage of starved cells over time under heterogeneous feeding in a microbioreactor with a volume of 0.16 µl and a stirring speed of 8000 µm/h including 60 g/l biomass of *E. coli* from coordinate x=500, y=0, and z=0.

References

1 *Hardy Diagnostics*, <https://catalog.hardydiagnostics.com/cp_prod/Content/hugo/Bifidobacterium.htm> (

2 Duncan, S. H. Growth requirements and fermentation products of Fusobacterium prausnitzii, and a proposal to reclassify it as Faecalibacterium prausnitzii gen. nov., comb. nov. *International Journal of Systematic and Evolutionary Microbiology* **52**, 2141-2146, doi:10.1099/ijs.0.02241-0 (2002).

3 Neidhardt, F. C. & Curtiss, R. *Escherichia coli and Salmonella : cellular and molecular biology*. (ASM Press, 1996).

4 Milo, R., Jorgensen, P., Moran, U., Weber, G. & Springer, M. BioNumbers—the database of key numbers in molecular and cell biology. *Nucleic Acids Research* **38**, D750-D753, doi:10.1093/nar/gkp889 (2010).

5 Klis, F. M., de Koster, C. G. & Brul, S. Cell wall-related bionumbers and bioestimates of Saccharomyces cerevisiae and Candida albicans. *Eukaryot Cell* **13**, 2-9, doi:10.1128/EC.00250-13 (2014).

6 Bremer, H. & Dennis, P. P. Modulation of Chemical Composition and Other Parameters of the Cell at Different Exponential Growth Rates. *EcoSal Plus* **3**, doi:10.1128/ecosal.5.2.3 (2008).

7 Shashkova, T. *et al.* Agent Based Modeling of Human Gut Microbiome Interactions and Perturbations. *PLoS One* **11**, e0148386, doi:10.1371/journal.pone.0148386 (2016).

8 Rahman, M., Hasan, M. R. & Shimizu, K. Growth phase-dependent changes in the expression of global regulatory genes and associated metabolic pathways in *Escherichia coli*. *Biotechnology letters* **30**, 853-860 (2008).
